# Integrating the Rabinowitz rarity framework with a National Plant Inventory in South Korea

**DOI:** 10.1002/ece3.4851

**Published:** 2019-01-13

**Authors:** Hyeyeong Choe, James H. Thorne, Robert Hijmans, Changwan Seo

**Affiliations:** ^1^ Department of Ecological Landscape Architecture Design Kangwon National University Chuncheon South Korea; ^2^ Department of Environmental Science and Policy UC Davis Davis California; ^3^ Division of Ecological Assessment National Institute of Ecology Seocheon South Korea

**Keywords:** geographic range size, habitat specificity, integrating data and theory, local abundance, National Species Survey, priority conservation areas, Rabinowitz rarity classes, rare plant species

## Abstract

Increasingly large presence‐only survey datasets are becoming available for use in conservation assessments. Potentially, these records could be used to determine spatial patterns of plant species rarity and endemism. We test the integration of a large South Korean species record database with Rabinowitz rarity classes. Rabinowitz proposed seven classes of species rarity using three variables: geographic range, habitat specificity, and local population size. We estimated the range size and local abundance of 2,215 plant species from species occurrence records and habitat specificity as the number of landcover types each species’ records were found in. We classified each species into a rarity class or as common, compared species composition by class to national lists, and mapped the spatial pattern of species richness for each rarity class. Species were classed to narrow or wide geographic ranges using 315 km, the average from a range size index of all species (*D*_max_), based on maximum distance between observations. There were four classes each within the narrow and wide range groups, sorted using cutoffs of local abundance and habitat specificity. Nationally listed endangered species only appeared in the narrow‐range classes, while nationally listed endemic species appeared in almost all classes. Species richness in most rarity classes was high in northeastern South Korea especially for species with narrow ranges. *Policy implications*. Large presence‐only surveys may be able to estimate some classes of rarity better than others, but modification to include estimates of local abundance and habitat types, could greatly increase their utility. Application of the Rabinowitz rarity framework to such surveys can extend their utility beyond species distribution models and can identify areas that need further surveys and for conservation priority. Future studies should be aware of the subjectivity of the rarity classification and that regional scale implementations of the framework may differ.

## 1 INTRODUCTION

Biological rarity in plants has been described in several ways, including edaphic endemism (Kruckeberg & Rabinowitz, 1985), having low population sizes (Flather & Sieg, 2013), or being phylogenetically unique (McKinney, 1997), and is essentially a function of a specie's attributes—geographic range, habitat specificity, and local population size (Rabinowitz, 1981). Large presence‐only survey datasets are increasingly available for use in conservation assessments. For example, the Global Biodiversity Information Facility (GBIF; http://www.gbif.org/) and many digitized herbaria collections such as the Australasian Virtual Herbarium (https://avh.chah.org.au/) and California's Jepson Herbaria (http://ucjeps.berkeley.edu/) contain species presence data. Such records have been used extensively for species distribution modeling (SDM) (Ancillotto, Strubbe, Menchetti, & Mori, 2016; Wright, Hijmans, Schwartz, & Shaffer, 2015), which can portray potential range. However, SDMs rely on assumptions that species are in equilibrium with current environmental conditions, and the distribution or abundance represents environmental tolerances and resource requirements (Franklin & Miller, 2009). Uncertainties in SDM results arise from the quantity and quality of observation data, from predictor variables used, and from errors in the modeling (Elith & Leathwick, 2009). Model results are influenced significantly by the sample size (Hernandez, Graham, Master, & Albert, 2006), and in many cases, the number of records available to fit a reliable model is limited, particularly for many rare species and regions (Choe, Thorne, & Seo, 2016; Wisz et al., 2008). Furthermore, the testing of the model performance is not sufficient in many SDM studies to convince ecologists (Vaughan & Ormerod, 2005).

On the other hand, species presence records could potentially be used in other ways, such as to determine plant rarity or endemism, which could offer a new tool for regional conservation efforts (Fleishman, Noss, & Noon, 2006). This approach is little‐tested, and modification of traditional definitions of rarity may be needed to transition from local to regional definitions of rarity (Sætersdal, 1994) and to integrate with large observation datasets. We asked how well a large survey of plant species observation records could identify different types of rarity and how well could it portray the resulting spatial patterns. To test the suitability of survey data for this type of analysis, we used a classification of species’ rarity types developed by Rabinowitz (1981) and applied it to a recently completed national‐scale species presence‐only survey for South Korean plants to see how well the survey could be used to define species’ rarity classes. We compared the resulting classification with nationally listed plant species lists and the IUCN Red List of Threatened Species, and examined the ability of the resulting spatial outputs to identify priority conservation areas and areas that need further species locality surveys in South Korea.

Rabinowitz (1981) proposed different types of species rarity using the following three variables: geographic range, habitat specificity, and local population size. These three variables can be combined and allow species to be classified into eight categories. Among them, seven types represent the different forms of rarity. These classes of rarity can be useful for conservation assessments, by using the basic characteristics of plant species from various literature sources and expert knowledge (Espeland & Emam, 2011; Silcock, Fensham, & Martin, 2011). For example, Broennimann, Vittoz, Moser, and Guisan (2005) classified Swiss conservation priority plant species into the Rabinowitz's rarity classes and found that species with the most restricted distribution are under higher risk of extinction by comparing rarity species and IUCN extinction risks. Caiafa and Martins (2010) used the forms of rarity from Rabinowitz's classification to divide southern Brazilian Atlantic rainforest species into rarity classes and found that 11% were represented in the most restricted rarity type. Anacker, Gogol‐Prokurat, Leidholm, and Schoenig (2013) classified California plant species into the eight types of rarity to create a list of focal species for conservation planning purposes.

We asked if using only the spatial patterns of vascular plant species’ observation records from a large inventory could render a suitable Rabinowitz classification. We used plant locality data from the South Korean “National Ecosystem Survey” (NES) which was conducted from 2006 to 2013 by teams of scientists who recorded plant observations, geographic information, and site conditions. The plant survey data consist of 149,048 observations for 2,215 plant species (Ministry of Environment & National Institute of Environmental Research, 2006).

We classified the plant species to respective rarity types and mapped plant species richness overall and plant species richness for each rarity class at various spatial resolutions to test the effect of spatial scale on the results. We used the subsequent spatial patterns to examine the congruence of the spatial patterns for the different rarity classes and use the patterns to identify potential conservation priority regions in South Korea and areas that may need further surveys. We show the strengths and limitations of using large systematic survey data to identify rarity classes for use in plant conservation. In addition, our approach provides a set of conservation plant lists for South Korea, and suggestions to complement ongoing national survey projects collecting natural ecosystem information by testing the accuracy and effectiveness of surveys.

## 2 METHODS

We classified each species into one of Rabinowitz's rarity classes by using occurrence points to estimate the three rarity descriptors. We estimated the geographic range size of each plant species by calculating the distance among its surveyed occurrence points. We calculated the average of minimum distances between occurrence points of the same species as a proxy for local abundance, and we calculated habitat specificity by examining how many types of land cover the observations for each species was found in, using a detailed landcover map.

### 2.1 Survey data

**Figure 1:**
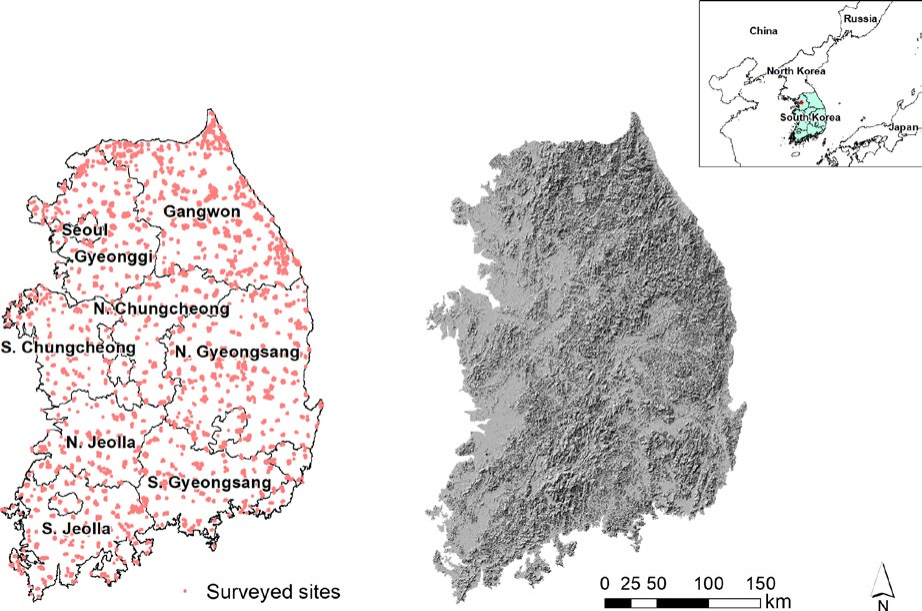
Locations of surveyed areas (left) and shaded topography (right) of South Korea

This study is confined to the mainland of South Korea which has an area of 95,219 km^2^_._ About 70% of the land area is mountainous (Figure 1). The South Korean Ministry of Environment has conducted three consecutive “National Ecosystem Surveys (NES)” since 1986. We used the most recent survey data for plants which were surveyed from 2006 to 2013, which is the largest single effort to document the distribution of vascular plants (Pteridophyta, Gymnosperm, and angiosperm) in South Korea to date (MOE & NIER, 2006). The data are available from the EcoBank (website: http://ecobank.nie.re.kr).

The NES survey design divides the country into a grid of 7,425 cells of 17.3 km^2^. A representative mountain area in each grid cell was selected to survey for plant species, and other sites representing additional habitats were added to seek species that might not occur in the habitats on the mountain surveyed. The representative mountains generally include the highest elevations in each survey unit, and they tend to hold high plant biodiversity. A linear path was walked to collect plant data and was selected to find as many plant species as possible, considering geographic status, diversity of habitats, vegetation naturalness, accessibility, and distribution of special plants (e.g., see Supporting Information Figure S1). These transects included various topographic features including ridge, slope, valley, and habitats, as well as streams and wetlands with aquatic plants. Surveys were carried out for all plant species encountered along transects which have different length at each survey and were apart to collect diverse species as much as possible in the mountain. Two people surveyed each transect one to three times in one year from February to November, mainly during spring and summer when plant species can be most easily determined. The survey data comprise a species name, date of survey, and the geographic location of each species (latitude and longitude).

First, we conducted an online review of the plants’ scientific names listed in Korea Biodiversity Information System website (http://www.nature.go.kr/ekbi/plant/clss/KBI_2001_010100.do) and compared to the survey data to confirm correct species names and to remove typos and synonyms. We used species with two or more occurrence records in this study. Thus, the original survey data consisting of 2,588 terrestrial vascular plant species with 149,421 records were reduced to 2,215 species with 149,048 occurrence records. These species represent 53.6% of the 4,130 plant species known to exist in South Korea (see Supporting Information Table S1; Choe et al., 2017). The South Korean Wildlife Protection and Management Act of 2004 (amended in 2014) designates 77 vascular plant species as endangered species, of which 11 were identified by 47 points in the national survey data we used. In addition, 114 out of total 434 nationally listed endemic species, defined as only inhabiting South Korea, were identified by 3,888 survey points in these survey data. The IUCN Red List of Threatened Species classifies 224 vascular plant species into threatened categories (28 species in critically endangered, 86 species in endangered, and 110 species in vulnerable species) in South Korea (National Institute of Biological Resources of Korean Ministry of Environment, 2012). In the survey data, 28 of these species were recorded (1 critically endangered, 6 endangered, and 21 vulnerable species).

### 2.2 Rarity measures

Rabinowitz proposed categories to classify species’ rarity types using geographic range (wide vs. narrow), habitat specificity (broad vs. restricted), and local population size (large vs. small). The three attributes combine for eight (2 × 2 × 2) classes. One of these (wide range, broadly adapted, and large local population) is not rare, leaving seven types of rarity.

We adapted Rabinowitz's classification of rarity and abundance to classify the South Korean plant species (Table 1). We used a range size statistic, maximum distance (*D*_max_) to estimate each species’ geographic range from the occurrence records (Hijmans & Spooner, 2001). *D*_max_ is the largest distance (in kilometers) between any pair of occurrence points of each species and was considered to represent range size. Range size can be calculated as the area using formal methods such as a convex polygon (Anacker et al., 2013; Gaston and Fuller, 2009). In a sensitivity analysis, we compared our range size metric with the convex polygon metric for species with three or more observations and the agreement rate was 85% (Table S1). Therefore, we used the *D*_max_ metric, in order to evaluate as many species as possible which includes 234 species (10.6% of analyzed species) with only two observation records.

**Table 1:** Species classification into eight categories using Rabinowitz's (1981) species rarity and the number of species in each class

Range	Specificity	Abundance	Code	All species	Endangered species	Endemic species	IUCN red lists
Narrow	Restricted	Small	N/R/S	384 (17.3%)	7	36	18
		Large	N/R/L	11 (0.5%)	∙	∙	∙
	Broad	Small	N/B/S	237 (10.7%)	3	11	4
		Large	N/B/L	283 (12.8%)	1	17	3
Wide	Restricted	Small	W/R/S	540 (24.4%)	∙	32	2
		Large	W/R/L	68 (3.1%)	∙	3	∙
	Broad	Small	W/B/S	193 (8.7%)	∙	3	1
		Large	Common (W/B/L)	499 (22.5%)	∙	11	∙

Note

In the table, the code is made up of the first letters of the binary classification of each index. We estimated the geographic range size of each plant species by calculating the distance among its surveyed occurrence points. We calculated the average of minimum distances between occurrence points of the same species as a proxy for local abundance, and we calculated habitat specificity by examining how many types of land cover the observations for each species was found in. We classified each species into the rarity framework using the average value of all species for each index.

In addition, we calculated the minimum distance to estimate each species’ local abundance. Minimum distance is the shortest distance (in kilometers) between occurrence points for each point of the same species. We assumed that the shorter distances between occurrence points meaning the higher local prevalence, and could be a proxy for abundance. We averaged the minimum distances of all occurrence points of each species and named this value as *D*_min_. We divided *D*_max_ by *D*_min_ and used this value as a proxy for local abundance. This proxy value increases as *D*_max_ is larger and *D*_min_ is smaller. Despite range size, if the distance between the observation records is short, the local abundance was considered to be large. All of the geographic calculations were conducted in R (version 3.5.1) by writing loop codes to iterate the calculations for each species.

$$D_{\max}=\text{The largest distance between any pair of occurrence points of each species}$$

$$D_{\min}=\text{Average of minimum distances between occurrence points for each point}$$

$$\text{Local abundance}=\frac{D_{\max}}{D_{\min}}$$

For habitat specificity, we used a landcover map of South Korea to examine the habitat specific of each species. The 2007 landcover map we used was derived from SPOT 5 and KOMPSAT 2, and has 23 categories including urbanized area, agricultural uses, forest, grass, wetlands, barren land, and water (website: http://egis.me.go.kr/map/map.do?type=land). We counted the number of landcover types overlapping with each species’ surveyed points as a proxy for habitat specificity.

Then, we classified each species into the rarity framework using the average value of all species for each index: *D*_max_, *D*_max_/*D*_min_, and habitat specificity. We classified all species into the eight categories and compared them to the national registration of endangered and endemic species and to the IUCN Red List species in Korea (Table 1) to assess how well the large dataset performed in identifying different classes of plants.

### 2.3 Species richness

We calculated overall species richness using grids with three spatial resolutions (10, 25, 50 km) because grid size can induce different spatial patterns (Seo, Thorne, Hannah, & Thuiller, 2009). Using smaller grid cells produces helpful richness patterns for the conservation in a local scale but also produces many empty areas due to the absence of survey data. On the other hand, using larger grids produces general richness patterns for a national scale, but may generalize the diversity patterns excessively (Orozco‐Ramírez, Perales, & Hijmans, 2016). We calculated species richness for the seven rarity classes, to find the spatial patterns of each species group using the 10 km resolution, because the rarity types were measurable at this finer resolution. To find conservation priority areas and areas needing additional surveys, we took the top 20% of richness areas from each of the eight species groups and combined them to see which areas contain many types of rarity.

## 3 RESULTS

### 3.1 National level species distributions

The number of occurrence records for each species varies from 2 to 762 (Table S1), with 24 species observed more than 500 times (9% of the survey data). The most frequently observed species were *Lindera obtusiloba* (762 observations), followed by *Zanthoxylum schinifolium* (701), *Stephanandra incise* (649), *Aster scaber* (595), and *Rhododendron mucronulatum* (592). In contrast, 234 species were observed only twice and 175 species were observed only three times. An additional 1,885 species known to exist in South Korea (Ministry of Environment, 2010) were not recorded, meaning the survey documents 54% of known plant species. All 11 listed endangered species were observed rarely: *Aconitum coreanum* (11 observations), *Aconitum austrokoreense* (9), *Cypripedium macranthos* (5), *Eleutherococcus senticosus* (5), *Echinosophora koreensis* (3), *Menyanthes trifoliata* (3), *Paeonia obovata* (3), *Astilboides tabularis* (2), *Drosera peltata* var. nipponica (2), *Thalictrum coreanum* (2), *Trientalis europaea* var. arctica (2).

The maximum distance between two observations of the same species (*D*_max_) ranges from 0.01 to 496 km. For geographic range size, the average of *D*_max_ index is 315 km. The average of minimum distances of all occurrence points of each species (*D*_min_) ranges from 0.01 to 439 km. For local abundance, the average of *D*_min_ index for all species is 42 km.

**Figure 2:**
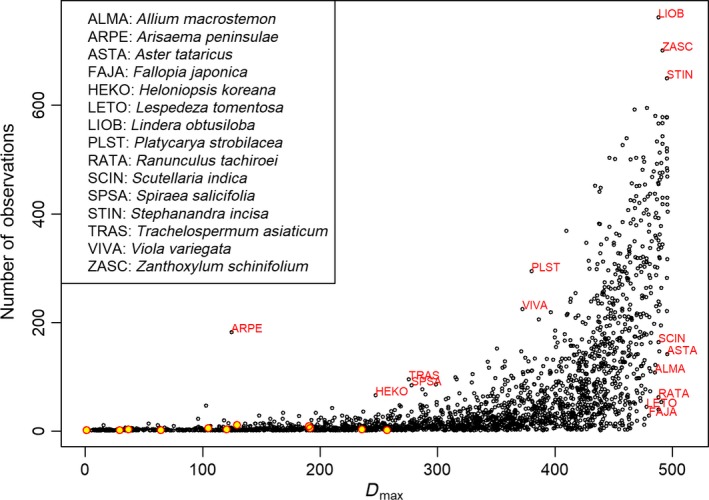
Relationships between the number of observations and the largest distance between two observations of a species. Red‐outlined yellow dots in the lower left of the image represent listed endangered species observed in the survey. Species named in Figure 2 have distinctive distribution patterns that are detailed in the Section 3

The number of observations of each species was positively correlated with *D*_max_ (Figure 2). *Lindera obtusiloba*,* Zanthoxylum schinifolium*, and *Stephanandra incisa* are widely distributed across the country with high frequency (upper right of Figure 2). We are interested in the species that have atypical relationships between the number of observations and *D*_max_. Species such as *Scutellaria indica, Aster tataricus, Allium macrostemon, Ranunculus tachiroei, Lespedeza tomentosa, and Fallopia japonica* have large *D*_max_ values and small numbers of observations, implying that they are widely distributed rare species (lower right of Figure 2). On the contrary, species such as *Trachelospermum asiaticum* (mainly in the southern coastal areas)*, Spiraea salicifolia* (in northern South Korea)*,* and *Heloniopsis koreana* (in the northeastern mountain areas) have relatively small *D*_max_ values and many observations, suggesting that they are abundant in relatively small areas (lower mid of Figure 2).

### 3.2 Species classification

**Figure 3:**
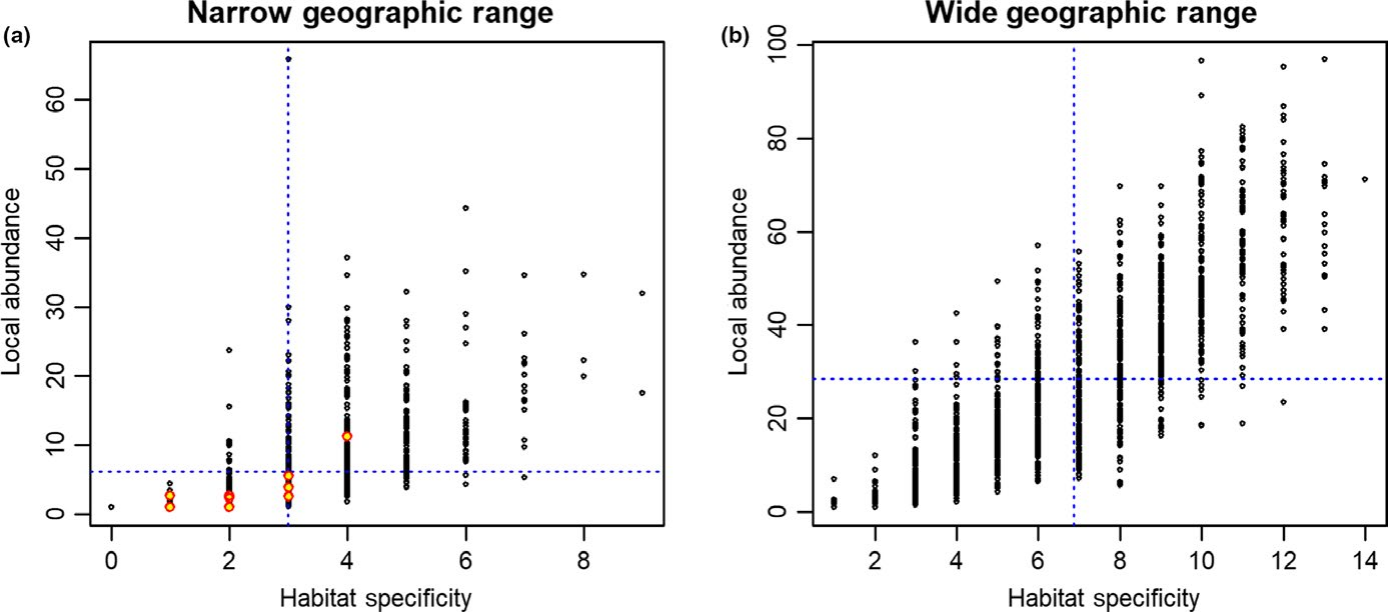
Species classification into eight categories using Rabinowitz's (1981) species rarity. Three variables were used to classify the species: Geographic range—Narrow (N) versus Wide (W), Habitat specificity—Restricted (R) versus Broad (B), and Local Abundance—Large (L) versus Small (S). Blue lines in both images represent the average value of each metric within the classification. Red‐outlined yellow dots in left image represent listed endangered species observed in the survey

Using the average *D*_max_ of all species as the cutoff, the number of species with a narrow geographic ranges was 915 and the number of species with wide geographic ranges was 1,300 (Table 1 and Figure 3). The local abundance index ranges from 1 to 96.8, and the average of local abundance index of all species was 19.3. Species’ observations occurred only on 16 of 23 landcover categories (86% of records were in coniferous forests, broadleaf forests, and mixed forests). The habitat specificity index ranges from 1 to 14, and the average of habitat specificity of all species was 5.3. There are four classes within both the narrow and the wide geographic range groups, divided by the average value of each local abundance and habitat specificity metric within the narrow and wide range groups.

Within the narrow geographic range species, the average habitat specificity was 3.0 landcover types/species and the average of local abundance was 6.1 (Figure 3a). The number of species in each rarity class is in Table 1. Species with narrow geographic range and restricted habitat specificity (N/R/S and N/R/L) are often endangered or threatened species (Rabinowitz, 1981; Turchetto et al., 2016). Our results identified 7 out of 11 listed endangered species, 36 out of 114 known South Korean endemic species, and 18 out of 28 IUCN Red List species as belonging to this restricted group. The other species we identify in these groups could be considered when updating lists of the national endangered species. Four of the listed endangered species in our data occupied the groups with narrow geographic range but broad habitat specificity.

Within the four groups with wide geographic range, species with restricted habitat specificity and small local abundance are generally predictable and are at risk of habitat destruction (Franklin & Miller, 2009). There are 193 species with wide ranges and broad habitat specificity but small abundance, that we termed widespread sparse species (W/B/S; the lower right of Figure 3b).

In this study, nationally listed endangered species only appear in the narrow geographic range classes, while the nationally listed South Korean endemic species appear in all groups except N/R/L (Table 1). The number of endemic species belonging to narrow‐range groups was 1.3 times higher compared to that of endemic species in wide range groups (Table 1).

### 3.3 Species richness

**Figure 4:**
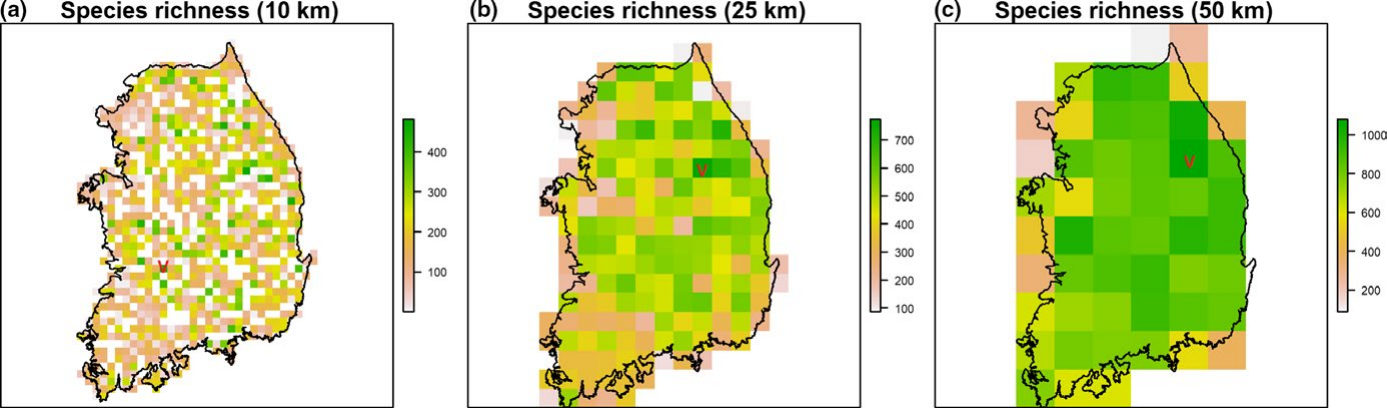
Species richness of all plant species on three grid resolutions (10, 25, 50 km). “V” represents the grids with the highest species richness values in each resolution grid

Areas (grid cells) with high overall species richness values are relatively evenly distributed across the country except in the southwestern parts of South Korea (Jeollanam province) at the lower resolution (10 km) (Figure 4). However, for the larger resolutions (25 and 50 km), the mountainous northeastern parts of the study area tend to contain the highest species richness areas. The cells with the highest species richness values in each resolution grid have 481 species in 10 km resolution, 773 in 25 km, and 1,078 in 50 km.

**Figure 5:**
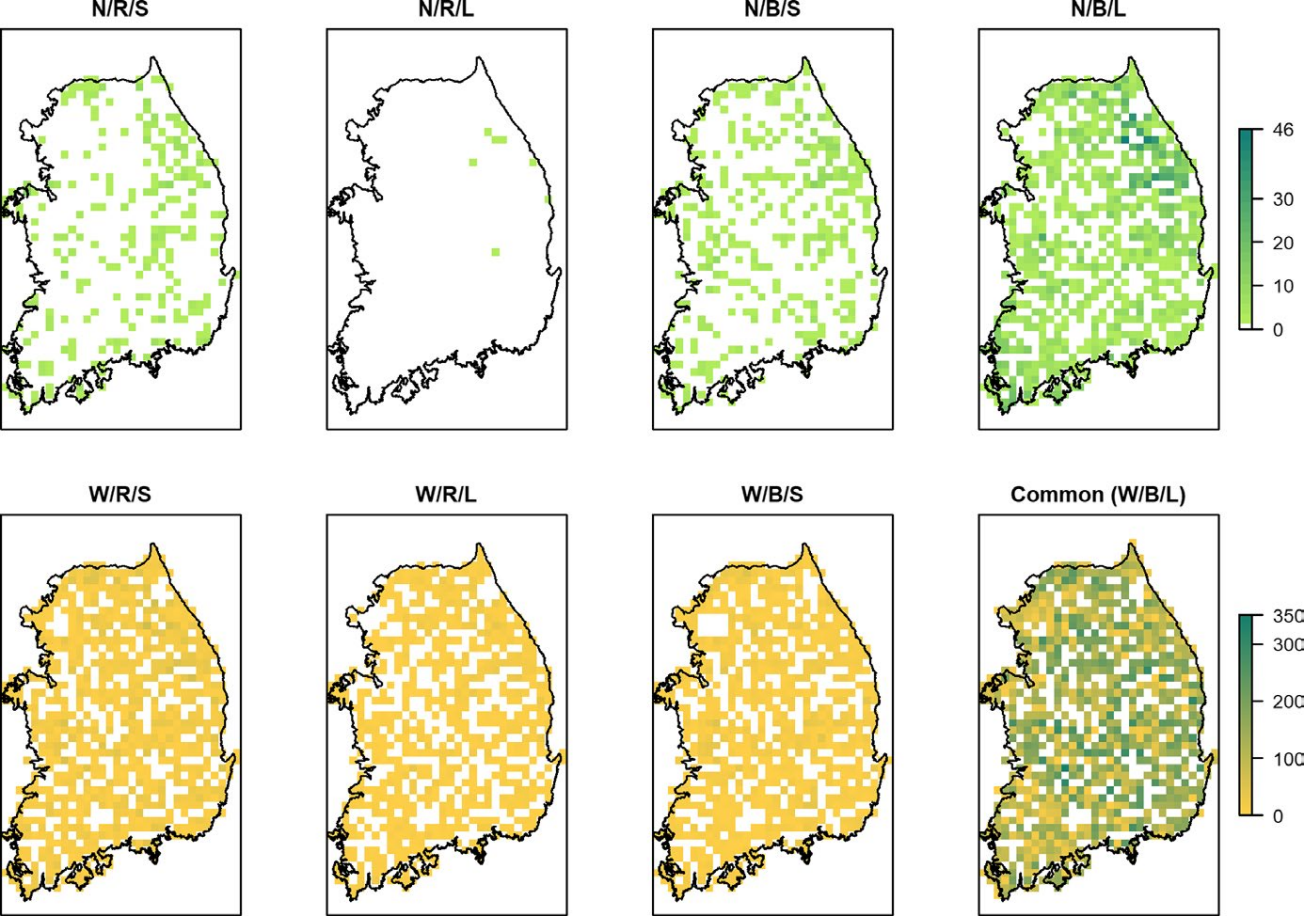
Species richness of the Rabinowitz rarity classes. Three variables were used to classify the species: Geographic range—Narrow (N) versus Wide (W), Habitat specificity ‐ Restricted (R) versus Broad (B), Local Abundance—Large (L) versus Small (S).

Species richness maps for each of the eight categories at 10 km resolution (Figure 5) show different spatial patterns from the overall species richness map (Figure 4). The species in rare on all counts group (narrow geographic range, low abundance, and restricted habitat specificity; N/R/S) are generally located in the central mountain ranges and the western lowland areas of the study area. The abundant species with narrow geographic range and restricted habitat specificity (N/R/L) are mostly in the northeastern parts of the country. Similarly, species with narrow geographic range but wide habitat specificity (N/B/S and N/B/L) are also largely distributed in the northeastern parts of South Korea. Species that have a wide range and restricted habitat specificity (W/R/S, Figure5) are broadly distributed. Widespread sparse species with broad habitat specificity but low abundance (W/B/S, Figure 5) spread also nationwide.

**Figure 6:**
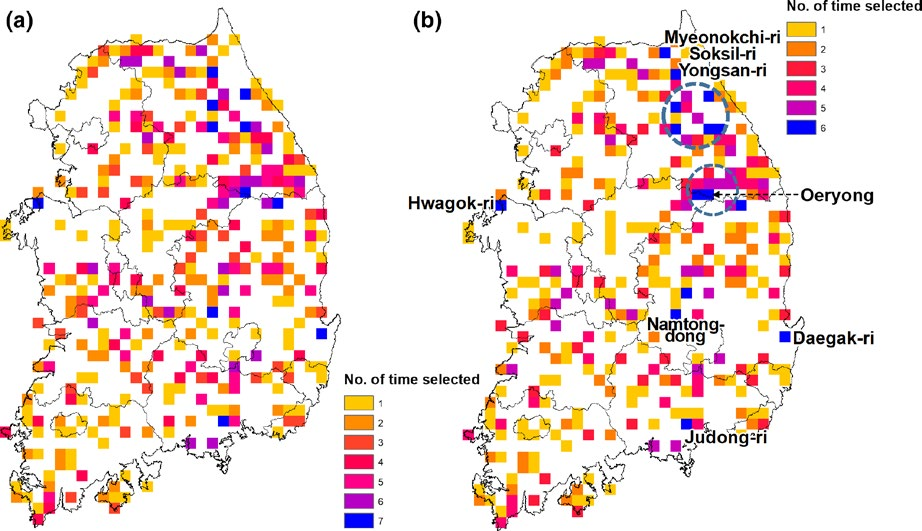
Spatial congruence of high species richness areas for the eight species classes (a) and for seven rarity classes, excluding common species group (b). We took the top 20% of species rich areas from each of the eight species classes and combined them to see how many types of rarity were found in each grid cell. We marked areas with the highest species richness for multiple rarirty classes with blue circles in Figure 6b

We combined the top 20% of richness areas from each of the eight species groups (Figure 6). Among the selected 34,600 km^2^ areas, 13,000 km^2^ (38%) are high richness areas from only one rarity type, while 1,100 km^2^ (3%) are high richness areas for the seven rarity groups, and 3,600 km^2^ (10%) are high richness areas for six rarity groups. High richness areas for five or more species groups cover 7,100 km^2^ (21%), and they are distributed mainly in the eastern parts of South Korea (Figure 6a). Especially, we note the area that has the highest species richness from the rarity classes, 10 × 10 km grid cells containing Myeonokchi, Soksil, Yongsan, and Oeryong villages in Gangwon Province, Namtong, Daegak, and Judong villages in Gyeongsang Province, and Hwagok in South Chungcheong Province (Figure 6b).

## 4 DISCUSSION

We combined a large presence‐only dataset of 2,215 plant species with the well‐known Rabinowitz rarity classification (1981) using only spatial data to classify the rarity status of multiple species and identify high species richness areas for each rarity class. The IUCN Red List Categories and Criteria are used to classify species at high risk of extinction into critically endangered, endangered, and vulnerable using all the available occupancy information (IUCN, 2012). Our approach can be easily used in other areas to identify each species’ rarity where there is insufficient information on species’ distributions and abundance. We found some rarity definitions worked better than others with implications for the application of this theoretical rarity classification to our national‐scale dataset. Here, we discuss our approach and provide suggestions for further national survey protocols and for plant conservation in South Korea.

### 4.1 Implications of the rarity framework for plant conservation in South Korea

When using the common species class or all species, high species richness areas are evenly distributed across the country using smaller grid cells (Figures 4 and 5). However, for species richness in the rarity categories, we found that Gangwon province (northeastern South Korea) contains the highest species richness, especially for species with narrow geographic ranges. The primary mountain ranges of South Korea, including the highly biodiverse Baekdudaegan Mountains (Choe et al., 2017, 2016; Ministry of Environment, 2012), are mostly located in Gangwon province. Our study also found high species richness of rarity classes and of nationally listed endangered and endemic species in each rarity class in these mountains (Figure 5 and Figure S2), which indicates our simple approach can identify high biodiversity areas for rare species. We suggest that further field surveys in these areas could help identify high priority plant conservation areas, that biological monitoring focused on tracking populations of narrow geographic range plant species be implemented in the northeastern parts of South Korea, and that regional urban development and land use plans should consider the preservation of the high biodiversity in this area (Choe & Thorne, 2017).

### 4.2 Scaling the Rabinowitz framework to a national scale

Rabinowitz considers species’ geographic range size an important attribute for classifying rarity. In our study, nationally listed South Korean endangered species appeared only in the narrow geographic range classes, and the large survey data were fairly successful in identifying narrow‐range species.

We assumed that the species observation data we used accurately represent the range distributions of plants in South Korea, but the application of Rabinowitz allowed us to think about the limits of the national survey data. Our dataset contained 435 species with two or three observations, including seven nationally listed endangered species and 35 endemic species. We included these species in order to retain the largest species list possible. However, these species comprise 86% of the N/R/S class, and we suspect this rarity class is inflated. We also acknowledge that unlisted species in this class may not be rare. However, the species we list should be further investigated, as they may be candidates for the nationally rare species list. Species with narrow geographic range but broad habitat specificity (N/B/L and N/B/S), are considered theoretically unlikely by Rabinowitz (Franklin & Miller, 2009; Rabinowitz, 1981), but they made up a large portion of species listed in the survey. These results could be due to incomplete surveys or local extinction meaning the species were actually more broadly distributed historically, or possibly due to error introduced by our measure of habitat breadth by intersecting the landcover map with the survey presence points. Potential error from the landcover map could be due to faulty landcover classification or conversion of landcover at a given observation point since the time (2007) of the mapping. It is also possible that this rarity definition does not scale as well to regional data‐driven analyses.

Rabinowitz defined endemic species as confined to specific habitat types within a small area but with large abundance, so species in our N/R/L classification are endemic species according to Rabinowitz. However, the nationally listed South Korean endemic species occur in almost all groups except N/R/L. The nationally listed endemic species had relatively large range sizes according to our measures, with a mean *D*_max_ of 252 km, compared to 315 km for all species. We checked attributes of the 11 species that we classed into the N/R/L group (Table 1). Among them, three are distributed nationwide but they were classified as narrow range because they lack records in the survey. These errors could be solved by modifying the survey protocols. The rest in our N/R/L group appear only in limited geographic areas with specific habitat types and were well classified using our approach. For nationally listed endemic species, species in N/R/S were a better fit, in which 36 of the 114 nationally listed endemics were classed. Therefore, the definition of endemism can have substantial consequences in the classification of large numbers of species using spatial data, which are likely context‐specific.

### 4.3 Spatially defined rarity indices representing Rabinowitz’ rarity classes

We devised the three rarity metrics using only spatial data that can be calculated in a rapid manner by writing loop codes. This approach provides useful alternatives when there is insufficient other information for species, but there are also limitations. For example, we classified each group using the average value of all species for each index, but it is also necessary to consider more meaningful cutoffs.

This section discusses the strengths and limitations of the three spatially defined rarity metrics we devised to classify species. For range size, we used maximum distance (*D*_max_) to estimate each species’ geographic range. However, 10.6% of the species we studied had only two observation records, and it is uncertain how well a distance metric can represent species’ range size. Therefore, we used a convex hull polygon approach to estimate range size (Gaston and Fuller, 2009) for species in our dataset with three or more observations, to test for agreement in the wide or narrow‐range size classification. Agreement between the approaches was 85% (Table S1), so we decided to use the *D*_max_ to evaluate as many species as possible for our study. This approach permitted us to estimate the range size of all species, but estimates of range size for species with low observation numbers and/or restricted habitats is uncertain.

For local abundance, we assumed shorter distances between occurrence points meant higher local abundance and divided *D*_max_ by *D*_min_ to calculate local abundance of each species. Many studies have found that species with wide range tend to have large abundance (Gaston & Lawton, 1990), so we devised the distance metric to calculate the local abundance using the maximum distance we used for the range size. This metric could be greatly improved with abundance records or potentially by plant functional type definitions (Soudzilovskaia et al., 2013), but we were limited to presence‐only records. However, by using this metric, we were able to estimate an abundance value for all species which may be correct, at least with regard to the values among species.

Habitat specificity is linked to the niche breadth of species, which can be related to measures such as soil type, moisture level, or successional state (Li, Nicotra, Xu, & Du, 2015). Rabinowitz describes habitat specificity as preferred habitat types of species, such as thickets or forest edges. This was the only metric we developed that used data external to the species occurrence records. We used a landcover map to count the number of different landcover types plant species occupied. Although this method does not require extensive literature review to identify each species’ characteristics, it has a drawback that it is influenced by both the quality of survey data and landcover map accuracy and could be improved with more detailed landcover maps and habitat description records.

### 4.4 Survey methods

Integrating the observation data with the rarity framework identified potential improvements for the South Korean national ecosystem survey protocols, which may extend to species record datasets generally. First, many species had few observation records but large *D*_max_ index values (Figure 2). Some of these species are widely distributed beyond South Korea. For example, *L. tomentosa* is broadly distributed in East Asia. However, based on the national survey, it is classified as widely distributed but rare, which suggests potential incomplete surveying, if indeed it is not rare in South Korea. On the other hand, *A. tataricus*,* R. tachiroei*,* F. japonica*, and *C. globosus* are distributed nationwide, but in wet conditions. This indicates a need for targeted inventories based on habitat types such as wetland areas. Such surveys could increase records for some species, and potentially increase the overall number of plant species represented in the survey, which is relatively low (64% of the flora) despite the seven years over which the survey was conducted.

Similarly, surveys should be strengthened in low‐elevation areas. The difference of 97 m elevation between areas with or without observation records (average elevation of white grids is 270 m, while average elevation of observations is 367 m.) indicates that more survey efforts are needed in smaller survey units and in low‐elevation areas. In addition, Choe, Thorne, Huber, Lee, and Quinn (2018) identified the importance of low‐elevation areas in conservation planning for South Korea. The un‐sampled grids from Figure 4a could be used to identify areas for additional surveys. Finally, surveyors walked extensive linear transects to collect plant species data. But, they recorded only a single occurrence for each species and did not record any metric of species’ abundance and habitat types. Future surveys could supplement this information by adopting the relevé sampling method, which is commonly used in plot surveys. Relevé methods record all species inside of a stand or plot and describe their abundance as well as occasionally habitat and soil types. This method can be efficient and useful to quickly classify the range of diversity for large‐scale projects (CNPS Vegetation Committee, 2007; Fidelibus & MacAller, 1993) and can provide long‐term reference data.

## 5 CONCLUSIONS

Many national‐scale surveys and biodiversity portals such as GBIF (Global Biodiversity Information Facility; http://www.gbif.org/), iNaturalist (http://www.inaturalist.org/), and MOL (Map Of Life; https://www.mol.org/) contain species presence records. While many of these datasets also contain measures of species abundance, our simple approach using presence‐only data can be used to extract more information from records that are already available and is therefore applicable to other regional or larger studies. In addition, although this study only considered species’ occurrences, application of species’ phylogenetic and functional types could provide useful information.

Plant species are one component of biodiversity, and areas we identified may not represent South Korea's overall biodiversity and comparison with species richness patterns from other taxonomic groups could improve the conservation target portfolio for South Korea (Dobson, Rodriguez, Roberts, & Wilcove, 1997). We found that presence‐only data have potential to be combined with rarity classifications with caveats about the definitions inherent in the rarity classification and potential disconnects when scaling up from local to regional implementations (Franklin, Wejnert, Hathaway, Rochester, & Fisher, 2009).

## CONFLICT OF INTEREST

None declared.

## AUTHORS’ CONTRIBUTIONS

H.C., J.T., and R.H. conceived the ideas and designed methodology; C.S. collected the data; H.C. and R.H. analyzed the data; and H.C. and J.T. led the writing of the manuscript. All authors contributed critically to the drafts and gave final approval for publication.

## Data Availability

Plant survey data: The national ecosystem survey was conducted by the Korean Ministry of Environment. The Korean National Institute of Ecology has uploaded these species data onto the EcoBank website: http://ecobank.nie.re.kr/eclgySpceInfo/eclgySpceInfo.do. To access the plant data, (a) Click “레이어선택”, which is the third button to the left from the top‐right corner of the linked site; (b) Click “전국자연환경조사(3차)”, the fourth item down; (c) Click the gray box to the left of the fourth item, “식물상”, then click “ok”; (d) Click the top‐right corner button; and (e) click the fourth button “속성정보(점)” (this is the left‐most button of the 2nd row of blue buttons); then click on one of the orange‐colored squares (observation points) on the screen. To download the species’ information, when the species list comes out, scroll all the way to the bottom of the list and click the blue button labeled “엑셀출력”. You can download species from this location in an Excel format. This download approach can be used for site‐by‐site data acquisition. For more information on these data, or to request a complete data set, contact Dr. Namsin Kim (geotop@nie.re.kr) at the Korean National Institute of Ecology. 2007 landcover map: The environmental data service provides landcover maps for various time periods on their website: http://egis.me.go.kr/map/map.do?type=land. To access the 2007 landcover map used in this study, (a) click “토지피복지도”, which is the first button from the top left corner of the linked site; (b) click “중분류”, the second item; (c) click the white box to the left of the second item, “[2007] 전국”, and browse the map of your interested area. To download the landcover map, you can define map name or administrative boundary, or select area, then, click “검색”, which is the blue oval button. You can download the maps in a PDF format.
