# Supplementary figures and images for: Integrating the Rabinowitz rarity framework with a National Plant Inventory in South Korea
Source: Ecol Evol. 2019 Jan 13;9(3):1353–63. doi: 10.1002/ece3.4851 (PMC6374650; doi:10.1002/ece3.4851)

**Figure S1. Example map of South Korean National Ecosystem Survey.**

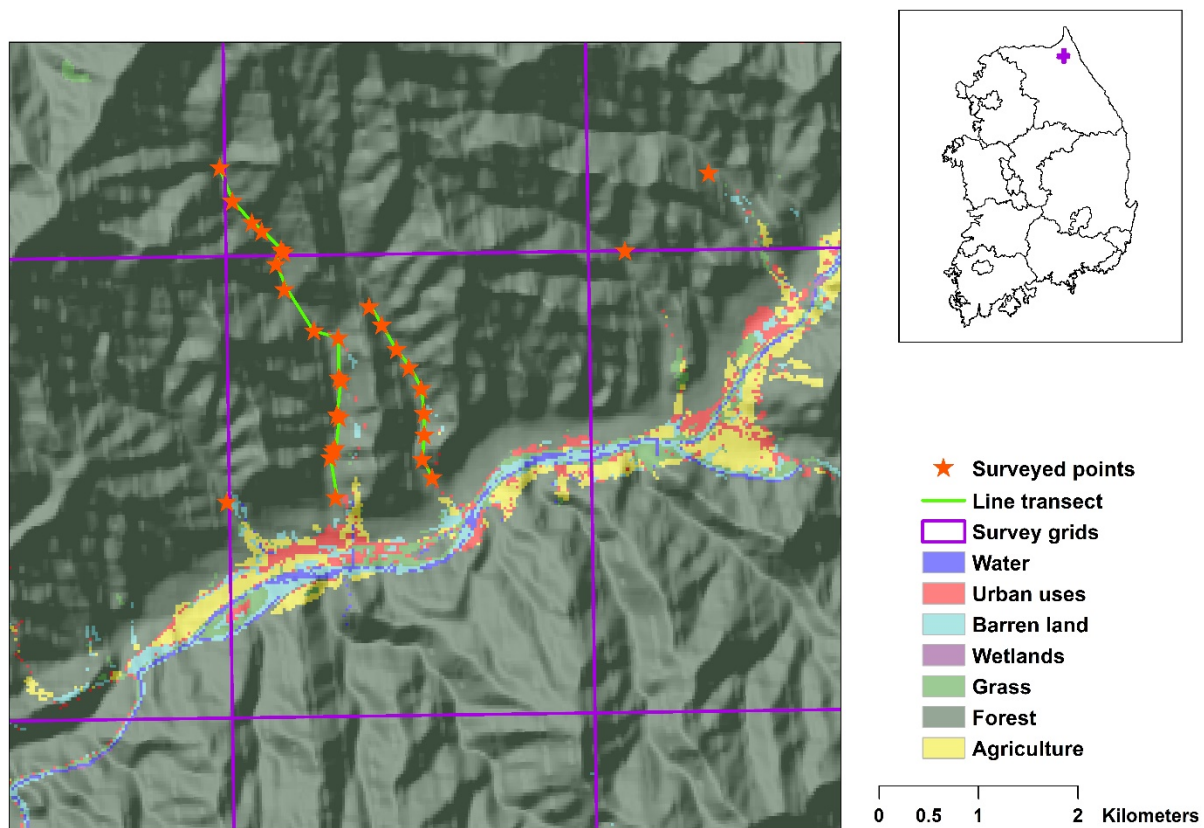

Supplement: Supplementary file 1 [file ECE3-9-1353-s001.pdf]
