# Supplementary material for: Integrating the Rabinowitz rarity framework with a National Plant Inventory in South Korea
Source: Ecol Evol. 2019 Jan 13;9(3):1353–63. doi: 10.1002/ece3.4851 (PMC6374650; doi:10.1002/ece3.4851)
Supplement: Supplementary file 2 [file ECE3-9-1353-s002.pdf]

Figure S2. The distributions of endangered species and endemic species.

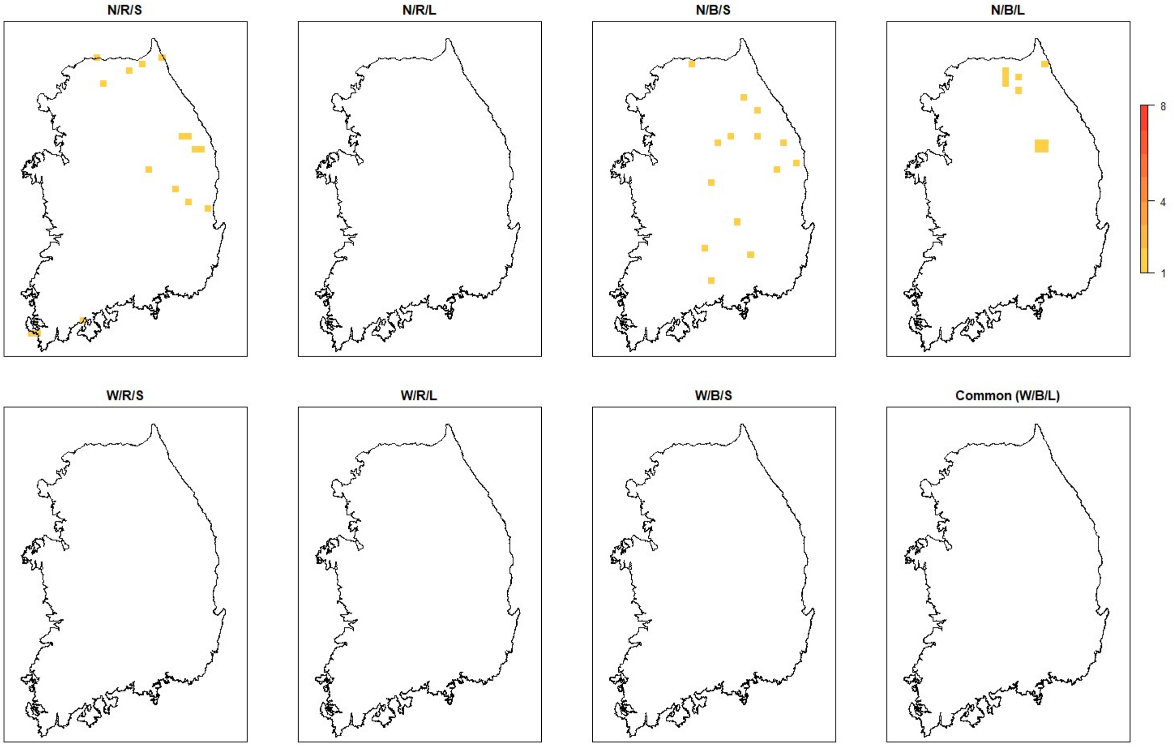

<Species Richness of Endangered Species using the Rabinowitz Rarity Classes>

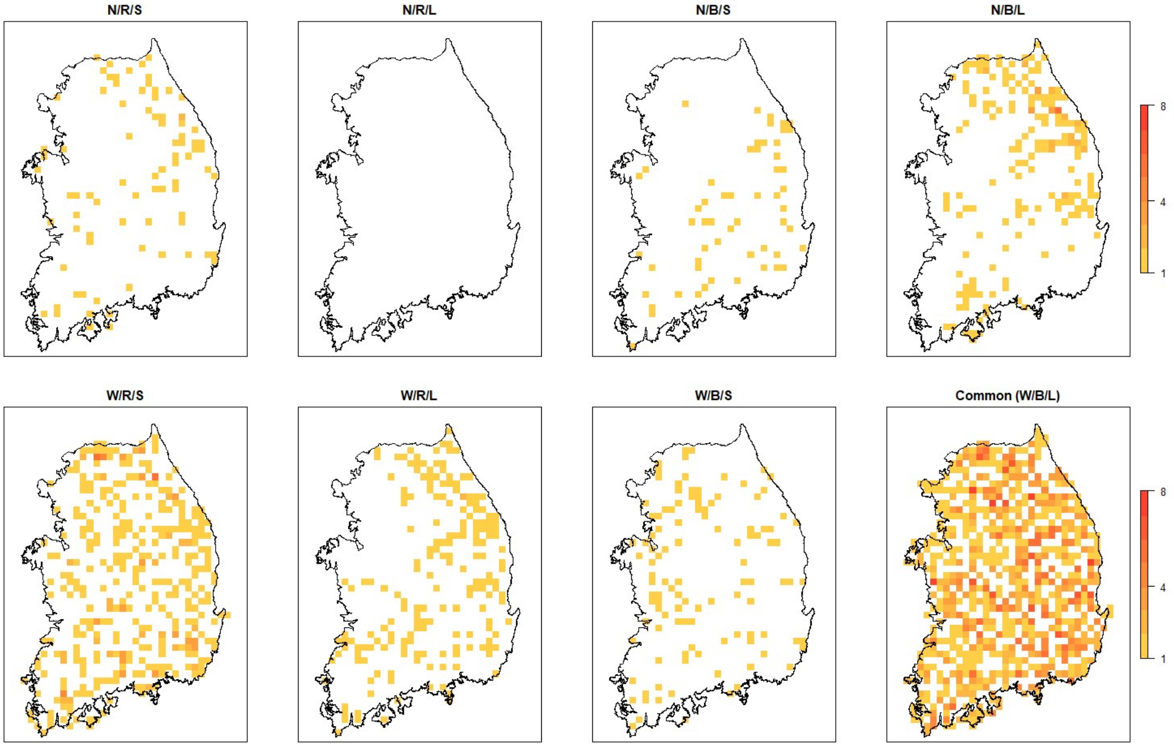

<Species Richness of Endemic Species using the Rabinowitz Rarity Classes>
