# Supplementary material for: Integrating the Rabinowitz rarity framework with a National Plant Inventory in South Korea
Source: Ecol Evol. 2019 Jan 13;9(3):1353–63. doi: 10.1002/ece3.4851 (PMC6374650; doi:10.1002/ece3.4851)
Supplement: Supplementary file 3 [file ECE3-9-1353-s003.docx]

| Species | No. of  Records | Endangered  Species | Endemic  Species | IUCN  Species | Dmax  (km) | Convex  Area  (km^2^) | Dmin  (km) | Local  Abundance | Habitat  Specificity | Group |
| --- | --- | --- | --- | --- | --- | --- | --- | --- | --- | --- |
| Abelia mosanensis T.H.Chung ex Nakai | 3 |  | V |  | 345.9 | 1311.0 | 156.1 | 2.2 | 3 | W/R/S |
| Abies holophylla Maxim. | 30 |  |  |  | 290.3 | 31313.8 | 17.4 | 16.7 | 5 | N/B/L |
| Abies koreana Wilson | 2 |  | V | EN | 215.1 | NA | 215.1 | 1.0 | 1 | N/R/S |
| Abies nephrolepis (Trautv.) Maxim. | 9 |  |  |  | 188.6 | 6977.4 | 27.3 | 6.9 | 3 | N/B/L |
| Abutilon theophrasti Medicus | 21 |  |  |  | 395.5 | 66532.8 | 37.7 | 10.5 | 6 | W/R/S |
| Acalypha australis L. | 233 |  |  |  | 463.9 | 98566.7 | 10.2 | 45.5 | 12 | W/B/L |
| Acer barbinerve Maxim. | 21 |  |  |  | 203.8 | 23346.4 | 18.5 | 11.0 | 4 | N/B/L |
| Acer buergerianum Miq. | 19 |  |  |  | 289.5 | 51866.9 | 40.4 | 7.2 | 5 | N/B/L |
| Acer komarovii Pojark. | 26 |  |  |  | 265.6 | 19360.2 | 17.0 | 15.6 | 3 | N/B/L |
| Acer mandshuricum Maxim. | 19 |  |  |  | 210.3 | 11873.7 | 15.6 | 13.5 | 4 | N/B/L |
| Acer micro-sieboldianum Nakai | 3 |  |  |  | 15.4 | 4.4 | 7.4 | 2.1 | 2 | N/R/S |
| Acer mono var. savatieri (Pax) Nakai | 3 |  |  |  | 333.1 | 4670.6 | 129.8 | 2.6 | 1 | W/R/S |
| Acer negundo L. | 2 |  |  |  | 255.6 | NA | 255.6 | 1.0 | 1 | N/R/S |
| Acer palmatum Thunb. ex Murray | 121 |  |  |  | 433.3 | 84505.4 | 12.7 | 34.2 | 8 | W/B/L |
| Acer palmatum var. pilosum Nakai | 2 |  |  |  | 306.6 | NA | 306.6 | 1.0 | 2 | N/R/S |
| Acer pictum subsp. mono (Maxim.) Ohashi | 245 |  |  |  | 448.7 | 88680.4 | 8.6 | 51.9 | 9 | W/B/L |
| Acer pictum var. pictum Thunb. ex Murray | 4 |  |  |  | 206.3 | 3430.3 | 64.7 | 3.2 | 3 | N/B/S |
| Acer pictum var. truncatum (Bunge) C.S.Chang | 36 |  |  |  | 422.0 | 68681.0 | 18.9 | 22.3 | 5 | W/R/S |
| Acer pseudo-sieboldianum (Pax) Kom. | 388 |  |  |  | 473.1 | 97435.9 | 6.6 | 71.6 | 10 | W/B/L |
| Acer pseudo-sieboldianum var. ishidoyanum Uyeki | 2 |  |  |  | 124.5 | NA | 124.5 | 1.0 | 2 | N/R/S |
| Acer pseudo-sieboldianum var. languinosum Nakai | 3 |  |  |  | 140.4 | 843.7 | 53.9 | 2.6 | 2 | N/R/S |
| Acer saccharinum L. | 2 |  |  |  | 161.9 | NA | 161.9 | 1.0 | 2 | N/R/S |
| Acer tataricum subsp. ginnala (Maxim.) Wesm. | 448 |  |  |  | 438.7 | 90112.9 | 6.2 | 70.7 | 13 | W/B/L |
| Acer tegmentosum Maxim. | 21 |  |  |  | 230.5 | 21777.1 | 13.5 | 17.0 | 4 | N/B/L |
| Acer triflorum Kom. | 66 |  |  |  | 436.0 | 64225.9 | 15.5 | 28.1 | 6 | W/R/S |
| Acer ukurunduense Trautv. & C.A.Mey. | 12 |  |  |  | 157.9 | 7542.9 | 15.3 | 10.3 | 2 | N/R/L |
| Achillea alpina L. | 44 |  |  |  | 335.4 | 44977.4 | 16.3 | 20.6 | 5 | W/R/S |
| Achillea alpina var. discoidea (Regel) Kitam. | 2 |  |  |  | 5.8 | NA | 5.8 | 1.0 | 1 | N/R/S |
| Achillea millefolium L. | 13 |  |  |  | 401.2 | 46575.6 | 47.1 | 8.5 | 5 | W/R/S |
| Achyranthes japonica (Miq.) Nakai | 264 |  |  |  | 454.1 | 100820.0 | 9.6 | 47.2 | 9 | W/B/L |
| Aconitum austrokoreense Koidz. | 9 | V | V | VU | 190.3 | 11104.7 | 34.4 | 5.5 | 3 | N/B/S |
| Aconitum ciliare DC. | 15 |  |  |  | 301.4 | 25791.8 | 36.5 | 8.3 | 5 | N/B/L |
| Aconitum coreanum (H. Lev.) Rapaics | 11 | V |  | VU | 129.3 | 4802.6 | 11.5 | 11.3 | 4 | N/B/L |
| Aconitum jaluense Kom. | 169 |  |  |  | 422.8 | 84896.6 | 10.0 | 42.2 | 10 | W/B/L |
| Aconitum kusnezoffii Rchb. | 12 |  |  |  | 304.0 | 21835.7 | 15.3 | 19.9 | 3 | N/B/L |
| Aconitum longecassidatum Nakai | 40 |  |  |  | 325.2 | 50613.6 | 16.8 | 19.4 | 5 | W/R/S |
| Aconitum macrohynchum Turcz. | 4 |  |  |  | 14.8 | 66.5 | 8.2 | 1.8 | 4 | N/B/S |
| Aconitum pseudolaeve Nakai | 47 |  |  |  | 102.9 | 64765.6 | 38.3 | 2.7 | 3 | N/B/S |
| Aconitum pseudo-proliferum Nakai | 4 |  | V |  | 351.7 | 2251.5 | 19.3 | 18.2 | 5 | W/R/S |
| Aconitum sibiricum Poir. | 2 |  |  | VU | 29.5 | NA | 29.5 | 1.0 | 2 | N/R/S |
| Aconitum triphyllum Nakai | 3 |  |  |  | 225.1 | 5781.2 | 121.0 | 1.9 | 2 | N/R/S |
| Aconogonon alpinum (All.) Schur | 61 |  |  |  | 472.5 | 66989.6 | 13.5 | 34.9 | 8 | W/B/L |
| Aconogonon divaricatum (L.) Nakai ex T.Mori | 3 |  |  |  | 309.8 | 10934.7 | 153.2 | 2.0 | 2 | N/R/S |
| Aconogonon microcarpum (Kitag.) H.Hara | 2 |  | V |  | 84.2 | NA | 84.2 | 1.0 | 1 | N/R/S |
| Acorus calamus L. | 13 |  |  | LC | 332.5 | 44348.5 | 42.2 | 7.9 | 4 | W/R/S |
| Acorus gramineus Sol. | 3 |  |  |  | 129.9 | 504.7 | 64.1 | 2.0 | 3 | N/B/S |
| Actaea asiatica H.Hara | 86 |  |  |  | 357.3 | 60039.9 | 11.9 | 30.1 | 3 | W/R/L |
| Actinidia arguta var. arguta (Siebold & Zucc.) Planch. ex Miq. | 212 |  |  |  | 459.3 | 88212.9 | 8.6 | 53.2 | 9 | W/B/L |
| Actinidia kolomikta (Maxim. & Rupr.) Maxim. | 63 |  |  |  | 434.0 | 71682.2 | 15.0 | 29.0 | 7 | W/B/L |
| Actinidia polygama (Siebold & Zucc.) Planch. ex Maxim. | 159 |  |  |  | 434.4 | 83590.7 | 10.1 | 43.2 | 7 | W/B/L |
| Actinostemma lobatum Maxim. | 10 |  |  |  | 351.2 | 45058.8 | 45.3 | 7.8 | 7 | W/B/S |
| Adenocaulon himalaicum Edgew. | 88 |  |  |  | 373.9 | 58014.5 | 11.6 | 32.2 | 6 | W/R/L |
| Adenophora curvidens Nakai | 3 |  |  |  | 282.1 | 3404.3 | 111.3 | 2.5 | 3 | N/B/S |
| Adenophora divaricata Franch. & Sav. | 56 |  |  |  | 459.7 | 68114.4 | 15.3 | 30.0 | 7 | W/B/L |
| Adenophora grandiflora Nakai | 5 |  |  |  | 319.7 | 36547.3 | 129.9 | 2.5 | 3 | W/R/S |
| Adenophora lamarckii Fisch. | 2 |  |  |  | 238.5 | NA | 238.5 | 1.0 | 1 | N/R/S |
| Adenophora palustris Kom. | 2 |  |  | EN | 239.5 | NA | 239.5 | 1.0 | 1 | N/R/S |
| Adenophora polyantha Nakai | 13 |  |  |  | 344.3 | 52108.6 | 32.9 | 10.5 | 5 | W/R/S |
| Adenophora remotiflora (Siebold & Zucc.) Miq. | 79 |  |  |  | 460.2 | 75499.0 | 13.0 | 35.3 | 6 | W/R/L |
| Adenophora stricta Miq. | 59 |  |  |  | 424.4 | 80186.5 | 15.1 | 28.1 | 6 | W/R/S |
| Adenophora triphylla var. japonica (Regel) H.Hara | 214 |  |  |  | 469.3 | 96804.3 | 9.9 | 47.4 | 10 | W/B/L |
| Adenophora verticillata var. hirsuta F.Schmidt | 9 |  |  |  | 257.5 | 33527.3 | 68.6 | 3.8 | 4 | N/B/S |
| Adenophora verticillata var. verticillata (Pall.) Fisch. | 50 |  |  |  | 435.7 | 79342.2 | 19.9 | 21.9 | 7 | W/B/S |
| Adenostemma lavenia (L.) Kuntze | 3 |  |  |  | 15.0 | 56.0 | 10.1 | 1.5 | 2 | N/R/S |
| Adonis amurensis Regel & Radde | 34 |  |  |  | 368.3 | 62859.1 | 20.0 | 18.4 | 6 | W/R/S |
| Adoxa moschatellina L. | 56 |  |  |  | 387.0 | 62306.5 | 19.7 | 19.7 | 6 | W/R/S |
| Aegopodium alpestre Ledeb. | 5 |  |  |  | 355.3 | 1551.0 | 69.6 | 5.1 | 3 | W/R/S |
| Aeschynomene indica L. | 79 |  |  |  | 422.4 | 69692.1 | 14.6 | 28.9 | 8 | W/B/L |
| Agastache rugosa (Fisch. & Mey.) Kuntze | 209 |  |  |  | 464.8 | 89331.1 | 10.4 | 44.8 | 8 | W/B/L |
| Agrimonia coreana Nakai | 56 |  |  |  | 350.1 | 62805.1 | 18.1 | 19.4 | 5 | W/R/S |
| Agrimonia pilosa Ledeb. | 567 |  |  |  | 491.4 | 105158.3 | 6.2 | 79.9 | 11 | W/B/L |
| Agrimonia pilosa var. nipponica (Koidz.) Kitamura | 4 |  |  |  | 214.5 | 3124.3 | 59.0 | 3.6 | 4 | N/B/S |
| Agropyron chinensis (Trin. ex Bunge) Ohwi | 5 |  |  |  | 190.0 | 1456.4 | 41.5 | 4.6 | 2 | N/R/S |
| Agropyron ciliare (Trin.) Franch. | 87 |  |  |  | 432.9 | 78000.8 | 16.1 | 26.9 | 8 | W/B/S |
| Agropyron ciliare for. hackelianum (Ohwi) Y.N.Lee | 2 |  |  |  | 24.8 | NA | 24.8 | 1.0 | 1 | N/R/S |
| Agropyron gmelini (Griseb.) Scribn. & Sm. | 4 |  |  |  | 298.9 | 12610.4 | 102.7 | 2.9 | 2 | N/R/S |
| Agropyron repens (L.) P.Beauv. | 4 |  |  |  | 322.3 | 10387.8 | 109.3 | 2.9 | 3 | W/R/S |
| Agropyron tsukushiense var. transiens (Hack.) Ohwi | 143 |  |  |  | 431.5 | 92226.8 | 12.2 | 35.4 | 10 | W/B/L |
| Agropyron yezoense var. yezoense Honda | 9 |  |  |  | 295.9 | 34227.2 | 43.9 | 6.7 | 3 | N/B/L |
| Agrostis alba L. | 7 |  |  |  | 408.9 | 46773.9 | 88.8 | 4.6 | 3 | W/R/S |
| Agrostis clavata var. clavata Trin. | 26 |  |  |  | 337.9 | 45655.0 | 24.2 | 13.9 | 5 | W/R/S |
| Agrostis clavata var. nukabo Ohwi | 63 |  |  |  | 451.4 | 82242.9 | 16.8 | 26.8 | 6 | W/R/S |
| Agrostis scabra Willd. | 6 |  |  |  | 295.8 | 24161.1 | 53.3 | 5.6 | 4 | N/B/S |
| Ailanthus altissima for. altissima (Mill.) Swingle | 108 |  |  |  | 396.4 | 77213.0 | 13.2 | 30.0 | 7 | W/B/L |
| Ainsliaea acerifolia Sch.Bip. | 293 |  |  |  | 473.6 | 93575.4 | 7.8 | 61.1 | 10 | W/B/L |
| Ajuga decumbens Thunb. | 90 |  |  |  | 328.9 | 52917.0 | 10.0 | 32.8 | 8 | W/B/L |
| Ajuga multiflora Bunge | 76 |  |  |  | 404.3 | 78122.0 | 15.0 | 27.0 | 10 | W/B/S |
| Ajuga spectabilis Nakai | 20 |  | V |  | 321.7 | 43568.4 | 31.4 | 10.2 | 3 | W/R/S |
| Akebia quinata (Thunb.) Decne. | 369 |  |  |  | 409.5 | 87430.5 | 6.8 | 60.2 | 11 | W/B/L |
| Alangium platanifolium (Siebold & Zucc.) Harms | 2 |  |  |  | 145.5 | NA | 145.5 | 1.0 | 2 | N/R/S |
| Alangium platanifolium var. trilobum (Miq.) Ohwi | 240 |  |  |  | 453.2 | 91487.3 | 9.1 | 50.0 | 10 | W/B/L |
| Albizia julibrissin Durazz. | 299 |  |  |  | 454.3 | 97818.0 | 8.1 | 55.7 | 10 | W/B/L |
| Albizia kalkora Prain | 3 |  |  | VU | 17.1 | 20.4 | 8.2 | 2.1 | 2 | N/R/S |
| Aletris spicata (Thunb.) Franch. | 2 |  |  |  | 13.8 | NA | 13.8 | 1.0 | 1 | N/R/S |
| Alisma canaliculatum A.Br. & Bouche | 2 |  |  |  | 272.7 | NA | 272.7 | 1.0 | 2 | N/R/S |
| Alisma orientale (Sam.) Juz. | 6 |  |  |  | 248.7 | 14911.7 | 43.2 | 5.8 | 3 | N/B/S |
| Allium macrostemon Bunge | 108 |  |  |  | 485.0 | 89597.1 | 13.2 | 36.6 | 8 | W/B/L |
| Allium monanthum Maxim. | 50 |  |  |  | 433.7 | 81651.2 | 18.6 | 23.3 | 5 | W/R/S |
| Allium sacculiferum Maxim. | 125 |  |  |  | 475.1 | 91595.6 | 11.2 | 42.6 | 9 | W/B/L |
| Allium senescens var. senescens L. | 2 |  |  |  | 171.8 | NA | 171.8 | 1.0 | 2 | N/R/S |
| Allium thunbergii G.Don | 127 |  |  |  | 457.6 | 96169.0 | 14.0 | 32.7 | 9 | W/B/L |
| Allium tuberosum Rottler ex Spreng. | 29 |  |  |  | 442.3 | 68243.2 | 32.6 | 13.6 | 5 | W/R/S |
| Alnus firma Siebold & Zucc. | 239 |  |  |  | 487.9 | 93459.4 | 6.9 | 70.9 | 10 | W/B/L |
| Alnus hirsuta Turcz. ex Rupr. | 311 |  |  |  | 477.7 | 101486.8 | 8.4 | 57.0 | 11 | W/B/L |
| Alnus japonica (Thunb.) Steud. | 53 |  |  |  | 467.2 | 79232.4 | 17.3 | 27.0 | 8 | W/B/S |
| Alnus mayrii Callier | 5 |  |  |  | 258.3 | 28897.9 | 108.9 | 2.4 | 3 | N/B/S |
| Alnus pendula Matsum. | 6 |  |  |  | 360.5 | 29976.8 | 66.9 | 5.4 | 4 | W/R/S |
| Alopecurus aequalis Sobol. | 182 |  |  |  | 432.6 | 93144.2 | 12.0 | 36.0 | 11 | W/B/L |
| Alopecurus pratensis L. | 2 |  |  |  | 227.2 | NA | 227.2 | 1.0 | 1 | N/R/S |
| Althaea rosea Cav. | 3 |  |  |  | 148.7 | 279.4 | 52.6 | 2.8 | 2 | N/R/S |
| Amaranthus deflexus L. | 5 |  |  |  | 216.0 | 7356.7 | 55.1 | 3.9 | 3 | N/B/S |
| Amaranthus lividus L. | 31 |  |  |  | 412.6 | 66184.9 | 29.3 | 14.1 | 5 | W/R/S |
| Amaranthus mangostanus L. | 51 |  |  |  | 390.4 | 72184.1 | 19.8 | 19.7 | 9 | W/B/S |
| Amaranthus patulus Bertol. | 32 |  |  |  | 331.2 | 45676.7 | 18.4 | 18.0 | 7 | W/B/S |
| Amaranthus retroflexus L. | 60 |  |  |  | 376.9 | 72960.2 | 15.5 | 24.2 | 9 | W/B/S |
| Amaranthus spinosus L. | 7 |  |  |  | 211.3 | 15795.6 | 31.7 | 6.7 | 3 | N/B/L |
| Amaranthus viridis L. | 5 |  |  |  | 238.7 | 3748.7 | 52.3 | 4.6 | 4 | N/B/S |
| Ambrosia artemisiifolia L. | 302 |  |  |  | 459.2 | 98346.6 | 8.9 | 51.4 | 11 | W/B/L |
| Ambrosia trifida var. trifida L. | 57 |  |  |  | 373.7 | 58133.8 | 13.0 | 28.7 | 7 | W/B/L |
| Amethystea caerulea L. | 30 |  |  |  | 261.6 | 28232.6 | 20.7 | 12.6 | 4 | N/B/L |
| Amitostigma gracilis (Blume) Schltr. | 54 |  |  |  | 390.4 | 69637.5 | 18.2 | 21.5 | 5 | W/R/S |
| Amorpha fruticosa L. | 301 |  |  |  | 486.9 | 104702.1 | 8.9 | 55.0 | 12 | W/B/L |
| Ampelopsis brevipedunculata (Maxim.) Trautv. | 394 |  |  |  | 485.4 | 103106.1 | 7.4 | 65.3 | 11 | W/B/L |
| Ampelopsis brevipedunculata for. ciliata (Nakai) T.B.Lee | 54 |  |  |  | 406.0 | 73594.6 | 14.8 | 27.4 | 8 | W/B/S |
| Ampelopsis brevipedunculata var. heterophylla for. citrulloides Rehder | 42 |  |  |  | 377.2 | 56089.8 | 17.2 | 21.9 | 6 | W/R/S |
| Amphicarpaea bracteata subsp. edgeworthii (Benth.) H.Ohashi | 235 |  |  |  | 439.8 | 85065.1 | 9.6 | 45.9 | 9 | W/B/L |
| Amsinckia lycopsoides Lehm. | 2 |  |  |  | 84.0 | NA | 84.0 | 1.0 | 2 | N/R/S |
| Anagallis arvensis for. coerulea (Schreb.) Naumgarten | 5 |  |  |  | 174.7 | 7831.9 | 56.7 | 3.1 | 3 | N/B/S |
| Anaphalis sinica Hance | 13 |  |  |  | 152.4 | 5577.4 | 12.3 | 12.4 | 4 | N/B/L |
| Andropogon ischaemum L. | 5 |  |  |  | 245.7 | 13336.5 | 67.7 | 3.6 | 4 | N/B/S |
| Androsace filiformis Retz. | 18 |  |  |  | 321.8 | 32152.3 | 28.2 | 11.4 | 8 | W/B/S |
| Androsace umbellata (Lour.) Merr. | 186 |  |  |  | 438.0 | 86631.5 | 10.8 | 40.4 | 11 | W/B/L |
| Aneilema keisak Hassk. | 42 |  |  |  | 442.2 | 60158.2 | 21.4 | 20.6 | 7 | W/B/S |
| Anemarrhena asphodeloides Bunge | 2 |  |  |  | 224.4 | NA | 224.4 | 1.0 | 2 | N/R/S |
| Anemone amurensis (Korsh.) Kom. | 6 |  |  |  | 152.0 | 4699.5 | 30.7 | 5.0 | 2 | N/R/S |
| Anemone koraiensis Nakai | 21 |  | V |  | 198.8 | 12980.2 | 14.9 | 13.3 | 4 | N/B/L |
| Anemone raddeana Regel | 57 |  |  |  | 392.8 | 82072.0 | 19.0 | 20.6 | 6 | W/R/S |
| Anemone reflexa Steph. & Willd. | 68 |  |  |  | 289.5 | 25961.6 | 10.0 | 29.0 | 6 | N/B/L |
| Anemone umbrosa C.A.Mey. | 3 |  |  | NT | 8.5 | 1.2 | 3.0 | 2.8 | 1 | N/R/S |
| Angelica acutiloba (Siebold & Zucc.) Kitag. | 9 |  |  |  | 306.0 | 22830.0 | 36.1 | 8.5 | 3 | N/B/L |
| Angelica anomala Ave-Lall. | 19 |  |  |  | 389.0 | 49735.8 | 32.9 | 11.8 | 5 | W/R/S |
| Angelica cartilagino-marginata (Makino) Nakai | 48 |  |  |  | 403.8 | 71936.7 | 21.4 | 18.9 | 11 | W/B/S |
| Angelica cartilagino-marginata var. distans (Nakai) Kitag. | 5 |  | V |  | 155.5 | 8678.8 | 61.2 | 2.5 | 3 | N/B/S |
| Angelica czernaevia (Fisch. & C.A.Mey.) Kitag. | 6 |  |  |  | 191.0 | 10403.5 | 53.6 | 3.6 | 3 | N/B/S |
| Angelica dahurica (Fisch. ex Hoffm.) Benth. & Hook.f. ex Franch. & S | 54 |  |  |  | 455.2 | 78929.7 | 18.1 | 25.1 | 8 | W/B/S |
| Angelica decursiva (Miq.) Franch. & Sav. | 185 |  |  |  | 463.1 | 92723.6 | 11.6 | 39.8 | 8 | W/B/L |
| Angelica genuflexa Nutt. ex Torr. & A.Gray | 6 |  |  |  | 330.0 | 2155.7 | 58.2 | 5.7 | 4 | W/R/S |
| Angelica gigas Nakai | 44 |  |  |  | 348.6 | 54308.7 | 16.6 | 21.0 | 4 | W/R/S |
| Angelica grosseserrata Maxim. | 163 |  |  |  | 464.2 | 85548.2 | 10.0 | 46.4 | 11 | W/B/L |
| Angelica koreana L. | 33 |  |  |  | 326.3 | 43945.2 | 16.9 | 19.3 | 7 | W/B/S |
| Angelica miqueliana Maxim. | 64 |  |  |  | 347.7 | 51499.8 | 15.4 | 22.6 | 6 | W/R/S |
| Angelica polymorpha Maxim. | 98 |  |  |  | 471.8 | 84314.0 | 14.4 | 32.7 | 8 | W/B/L |
| Angelica tenuissima Nakai | 13 |  |  |  | 366.1 | 30417.3 | 26.6 | 13.8 | 4 | W/R/S |
| Anthoxanthum odoratum var. odoratum L. | 2 |  |  |  | 165.9 | NA | 165.9 | 1.0 | 2 | N/R/S |
| Anthriscus sylvestris (L.) Hoffm. | 42 |  |  |  | 445.8 | 84837.5 | 24.2 | 18.4 | 6 | W/R/S |
| Aphananthe aspera (Thunb.) Planch. | 9 |  |  |  | 244.5 | 8823.8 | 20.5 | 11.9 | 4 | N/B/L |
| Aquilegia buergeriana var. oxysepala (Traut. & Meyer) Kitamura | 4 |  |  |  | 65.2 | 204.2 | 19.8 | 3.3 | 3 | N/B/S |
| Aquilegia japonica Nakai & H.Hara | 4 |  |  |  | 259.7 | 2876.0 | 69.9 | 3.7 | 2 | N/R/S |
| Aquilegia oxysepala Trautv. & C.A.Mey. | 25 |  |  |  | 212.2 | 18623.6 | 17.5 | 12.1 | 6 | N/B/L |
| Arabidopsis thaliana (L.) Heynh. | 3 |  |  |  | 213.1 | 1000.9 | 76.6 | 2.8 | 2 | N/R/S |
| Arabis columnaris Nakai | 4 |  | V |  | 229.5 | 2003.8 | 43.7 | 5.3 | 2 | N/R/S |
| Arabis gemmifera (Matsum.) Makino | 14 |  |  |  | 156.6 | 6399.6 | 12.5 | 12.5 | 3 | N/B/L |
| Arabis glabra Bernh. | 214 |  |  |  | 477.9 | 89933.8 | 10.7 | 44.8 | 9 | W/B/L |
| Arabis hirsuta (L.) Scop. | 43 |  |  |  | 428.6 | 65800.2 | 16.1 | 26.6 | 3 | W/R/S |
| Arabis lyrata L. | 6 |  |  |  | 192.5 | 10157.2 | 40.4 | 4.8 | 3 | N/B/S |
| Arabis pendula L. | 20 |  |  |  | 263.8 | 30821.8 | 22.8 | 11.6 | 5 | N/B/L |
| Arachniodes borealis Seriz. | 60 |  |  |  | 408.8 | 68927.0 | 15.5 | 26.5 | 6 | W/R/S |
| Arachniodes mutica (Franch. & Sav.) Ohwi | 3 |  |  |  | 30.4 | 1.9 | 10.2 | 3.0 | 2 | N/R/S |
| Aralia cordata Thunb. | 6 |  |  |  | 190.2 | 9056.1 | 42.1 | 4.5 | 3 | N/B/S |
| Aralia cordata var. continentalis (Kitag.) Y.C.Chu | 122 |  |  |  | 426.0 | 83415.5 | 12.3 | 34.7 | 7 | W/B/L |
| Aralia elata (Miq.) Seem. | 256 |  |  |  | 440.6 | 95332.3 | 8.7 | 50.9 | 11 | W/B/L |
| Aralia elata for. rotundata (Nakai) W.T.Lee | 5 |  | V |  | 93.5 | 741.1 | 7.7 | 12.1 | 3 | N/B/L |
| Arctium lappa L. | 4 |  |  |  | 214.5 | 5451.0 | 78.8 | 2.7 | 3 | N/B/S |
| Ardisia crenata Sims | 3 |  |  | LC | 7.0 | 0.3 | 2.4 | 2.9 | 2 | N/R/S |
| Ardisia japonica (Thunb.) Blume | 10 |  |  |  | 207.1 | 9602.2 | 28.8 | 7.2 | 4 | N/B/L |
| Arenaria serpyllifolia L. | 197 |  |  |  | 459.5 | 94306.4 | 11.9 | 38.5 | 11 | W/B/L |
| Argusia sibirica (L.) Dandy | 4 |  |  |  | 325.8 | 19755.1 | 110.2 | 3.0 | 3 | W/R/S |
| Arisaema amurense for. serratum (Nakai) Kitag. | 172 |  |  |  | 455.5 | 90863.7 | 9.1 | 49.9 | 8 | W/B/L |
| Arisaema amurense Maxim. | 70 |  |  |  | 410.7 | 80408.1 | 14.1 | 29.2 | 6 | W/R/L |
| Arisaema heterophyllum Blume | 12 |  |  |  | 370.3 | 25170.4 | 41.4 | 9.0 | 4 | W/R/S |
| Arisaema peninsulae for. convolutum (Nakai) Y.S.Kim & S.T.Ko | 15 |  | V |  | 455.2 | 7481.4 | 9.6 | 47.2 | 7 | W/B/L |
| Arisaema peninsulae Nakai | 182 |  |  |  | 124.6 | 86583.9 | 9.2 | 13.5 | 3 | N/B/L |
| Arisaema ringens (Thunb.) Schott | 34 |  |  |  | 385.3 | 61258.8 | 18.5 | 20.8 | 5 | W/R/S |
| Arisaema robustum (Engl.) Nakai | 74 |  |  |  | 426.4 | 76838.1 | 13.1 | 32.5 | 6 | W/R/L |
| Arisaema robustum var. purpureum Nakai | 4 |  |  |  | 272.8 | 5441.4 | 75.8 | 3.6 | 3 | N/B/S |
| Arisaema thunbergii Blume | 2 |  |  |  | 182.5 | NA | 182.5 | 1.0 | 1 | N/R/S |
| Aristolochia contorta Bunge | 64 |  |  |  | 338.4 | 51634.0 | 14.8 | 22.9 | 7 | W/B/S |
| Aristolochia manshuriensis Kom. | 37 |  |  |  | 302.8 | 33291.8 | 10.8 | 28.0 | 3 | N/B/L |
| Arrhenatherum elatius (L.) P.Beauv. ex J.Presl & C.Presl | 20 |  |  |  | 418.0 | 43813.1 | 25.8 | 16.2 | 5 | W/R/S |
| Artemisia argyi H.Lev. & Vaniot | 4 |  |  |  | 414.8 | 38487.4 | 169.3 | 2.4 | 3 | W/R/S |
| Artemisia capillaris Thunb. | 144 |  |  |  | 423.8 | 87219.0 | 12.5 | 33.9 | 11 | W/B/L |
| Artemisia dubia Wall. | 26 |  |  |  | 324.7 | 45825.6 | 24.9 | 13.1 | 7 | W/B/S |
| Artemisia feddei H.Lev. & Vaniot | 51 |  |  |  | 404.6 | 77905.9 | 20.5 | 19.8 | 7 | W/B/S |
| Artemisia gmelini Weber ex Stechm. | 126 |  |  |  | 439.9 | 64209.4 | 10.8 | 40.7 | 10 | W/B/L |
| Artemisia japonica Thunb. | 198 |  |  |  | 484.3 | 91534.3 | 11.2 | 43.2 | 10 | W/B/L |
| Artemisia japonica var. angustissima (Nakai) Kitam. | 10 |  | V |  | 167.4 | 11099.4 | 27.9 | 6.0 | 4 | N/B/S |
| Artemisia keiskeana Miq. | 456 |  |  |  | 465.4 | 102126.6 | 6.9 | 67.7 | 10 | W/B/L |
| Artemisia koidzumii Nakai | 29 |  |  |  | 422.6 | 57876.5 | 27.5 | 15.4 | 6 | W/R/S |
| Artemisia montana (Nakai) Pamp. | 77 |  |  |  | 397.2 | 67200.6 | 15.7 | 25.3 | 8 | W/B/S |
| Artemisia princeps Pamp. | 379 |  |  |  | 469.2 | 102248.0 | 7.3 | 64.4 | 11 | W/B/L |
| Artemisia rubripes Nakai | 30 |  |  |  | 363.7 | 60671.0 | 23.9 | 15.2 | 5 | W/R/S |
| Artemisia scoparia Waldst. & Kit. | 22 |  |  |  | 361.6 | 61885.4 | 18.7 | 19.3 | 6 | W/R/S |
| Artemisia selengensis Turcz. ex Besser | 15 |  |  |  | 204.0 | 17562.9 | 19.6 | 10.4 | 6 | N/B/L |
| Artemisia stolonifera for. stolonifera (Maxim.) Kom. | 240 |  |  |  | 436.3 | 86443.8 | 9.3 | 47.0 | 9 | W/B/L |
| Artemisia sylvatica Maxim. | 23 |  |  |  | 392.7 | 68618.4 | 32.9 | 11.9 | 5 | W/R/S |
| Artemisia viridissima (Kom.) Pamp. | 2 |  |  |  | 17.2 | NA | 17.2 | 1.0 | 2 | N/R/S |
| Arthraxon hispidus (Thunb.) Makino | 100 |  |  |  | 461.3 | 88649.8 | 15.8 | 29.2 | 7 | W/B/L |
| Aruncus dioicus var. kamtschaticus (Maxim.) H.Hara | 22 |  |  |  | 298.5 | 30849.6 | 18.9 | 15.8 | 6 | N/B/L |
| Arundinaria simonii (Carriere) A. & C.Riviere | 5 |  |  |  | 163.2 | 3856.2 | 38.7 | 4.2 | 3 | N/B/S |
| Arundinella hirta (Thunb.) Koidz. | 294 |  |  |  | 457.5 | 94821.9 | 7.8 | 58.6 | 9 | W/B/L |
| Arundinella hirta var. ciliata Koidz. | 42 |  |  |  | 391.3 | 61930.5 | 14.3 | 27.3 | 8 | W/B/S |
| Asarum heterotropoides F.Schmidt | 22 |  |  |  | 302.6 | 13543.1 | 13.4 | 22.6 | 7 | N/B/L |
| Asarum koreanum (J.Kim & C.Yook) B.U.Oh & J.K.Kim | 3 |  |  |  | 26.8 | 9.8 | 9.4 | 2.9 | 2 | N/R/S |
| Asarum maculatum Nakai | 15 |  | V |  | 372.1 | 51380.8 | 13.2 | 28.2 | 3 | W/R/S |
| Asarum misandrum B.U.Oh & J.K.Kim | 2 |  | V |  | 21.1 | NA | 21.1 | 1.0 | 1 | N/R/S |
| Asarum patens (K.Yamaki) B.U.Oh | 18 |  |  |  | 288.2 | 35337.3 | 24.4 | 11.8 | 5 | N/B/L |
| Asarum sieboldii Miq. | 383 |  |  |  | 464.2 | 99742.1 | 7.1 | 65.6 | 9 | W/B/L |
| Asarum versicolor (K.Yamaki) Y.N.Lee | 5 |  |  |  | 312.9 | 10920.2 | 79.5 | 3.9 | 3 | N/B/S |
| Asparagus cochinchinensis (Lour.) Merr. | 13 |  |  |  | 303.3 | 21503.2 | 19.0 | 15.9 | 3 | N/B/L |
| Asparagus davuricus Fisch. ex Link | 2 |  |  |  | 0.1 | NA | 0.1 | 1.0 | 2 | N/R/S |
| Asparagus officinalis | 2 |  |  |  | 3.1 | NA | 3.1 | 1.0 | 1 | N/R/S |
| Asparagus oligoclonos Maxim. | 70 |  |  |  | 422.6 | 84589.1 | 16.7 | 25.3 | 6 | W/R/S |
| Asparagus schoberioides Kunth | 252 |  |  |  | 440.3 | 93355.7 | 9.2 | 47.6 | 9 | W/B/L |
| Asperula lasiantha Nakai | 27 |  | V |  | 333.0 | 50182.7 | 18.2 | 18.3 | 5 | W/R/S |
| Asperula maximowiczii Kom. | 164 |  |  |  | 448.9 | 77041.4 | 9.0 | 50.0 | 9 | W/B/L |
| Asperula odorata L. | 19 |  |  |  | 277.8 | 33356.7 | 21.6 | 12.9 | 4 | N/B/L |
| Asperula platygalium Maxim. | 2 |  |  |  | 198.8 | NA | 198.8 | 1.0 | 2 | N/R/S |
| Asplenium incisum Thunb. | 318 |  |  |  | 477.7 | 97934.1 | 7.1 | 67.6 | 11 | W/B/L |
| Asplenium ruprechtii Sa.Kurata | 105 |  |  |  | 329.7 | 61001.4 | 10.7 | 30.7 | 9 | W/B/L |
| Asplenium rutamurarium L. | 2 |  |  | LC | 5.3 | NA | 5.3 | 1.0 | 2 | N/R/S |
| Asplenium sarelii Hook. | 5 |  |  |  | 229.7 | 17206.0 | 55.7 | 4.1 | 3 | N/B/S |
| Asplenium trichomanes L. | 2 |  |  | NT | 108.2 | NA | 108.2 | 1.0 | 2 | N/R/S |
| Asplenium varians Wall. ex Hook. & Grev. | 6 |  |  |  | 241.2 | 16871.5 | 56.2 | 4.3 | 2 | N/R/S |
| Aster ageratoides subsp. amplexifolius | 3 |  |  |  | 247.0 | 14918.7 | 165.9 | 1.5 | 3 | N/B/S |
| Aster ageratoides var. ageratoides Turcz. | 340 |  |  |  | 474.4 | 99900.8 | 7.8 | 60.6 | 10 | W/B/L |
| Aster fastigiatus Fisch. | 10 |  |  |  | 320.8 | 24946.0 | 36.9 | 8.7 | 4 | W/R/S |
| Aster maackii Regel | 8 |  |  |  | 225.5 | 17410.5 | 23.2 | 9.7 | 3 | N/B/L |
| Aster pilosus Willd. | 207 |  |  |  | 465.1 | 97243.1 | 10.3 | 45.2 | 10 | W/B/L |
| Aster scaber Thunb. | 595 |  |  |  | 478.1 | 106052.7 | 5.8 | 82.4 | 11 | W/B/L |
| Aster spathulifolius Maxim. | 7 |  |  |  | 463.1 | 59294.3 | 80.9 | 5.7 | 4 | W/R/S |
| Aster subulatus Michx. | 20 |  |  |  | 429.6 | 73711.3 | 25.9 | 16.6 | 6 | W/R/S |
| Aster subulatus var. sandwicensis A.G.Jones | 6 |  |  |  | 360.5 | 45011.8 | 90.8 | 4.0 | 4 | W/R/S |
| Aster tataricus L.f. | 142 |  |  |  | 495.4 | 79532.0 | 11.9 | 41.7 | 11 | W/B/L |
| Aster tripolium L. | 3 |  |  |  | 288.0 | 1093.8 | 100.6 | 2.9 | 2 | N/R/S |
| Astilbe koreana (Kom.) Nakai | 30 |  |  |  | 351.7 | 52516.3 | 19.5 | 18.1 | 5 | W/R/S |
| Astilbe rubra var. rubra Hook.f. & Thomson | 390 |  |  |  | 466.6 | 93983.5 | 7.1 | 65.7 | 10 | W/B/L |
| Astilboides tabularis (Hemsl.) Engl. | 2 | V |  | VU | 29.3 | NA | 29.3 | 1.0 | 1 | N/R/S |
| Astragalus koraiensis Y.N.Lee | 5 |  | V |  | 43.8 | 593.0 | 13.9 | 3.1 | 3 | N/B/S |
| Astragalus membranaceus var. membranaceus Bunge | 8 |  |  |  | 304.0 | 19514.2 | 56.7 | 5.4 | 3 | N/B/S |
| Astragalus sinicus L. | 46 |  |  |  | 290.7 | 39603.2 | 18.2 | 15.9 | 6 | N/B/L |
| Asyneuma japonicum (Miq.) Briq. | 133 |  |  |  | 408.9 | 74599.0 | 10.7 | 38.1 | 9 | W/B/L |
| Athyrium acutipinnulum Kodama ex Nakai | 2 |  | V |  | 197.9 | NA | 197.9 | 1.0 | 2 | N/R/S |
| Athyrium brevifrons Nakai ex Kitag. | 65 |  |  |  | 344.8 | 55819.8 | 13.5 | 25.6 | 6 | W/R/S |
| Athyrium concinnum Nakai | 3 |  |  |  | 184.4 | 5008.3 | 106.2 | 1.7 | 3 | N/B/S |
| Athyrium iseanum Rosenst. | 50 |  |  |  | 442.7 | 72423.8 | 20.8 | 21.3 | 6 | W/R/S |
| Athyrium koryoense Tagawa | 6 |  |  |  | 320.0 | 35130.2 | 81.9 | 3.9 | 4 | W/R/S |
| Athyrium nikkoense Makino | 6 |  |  |  | 263.5 | 26323.4 | 79.6 | 3.3 | 3 | N/B/S |
| Athyrium niponicum (Mett.) Hance | 207 |  |  |  | 447.3 | 83863.0 | 8.5 | 52.5 | 12 | W/B/L |
| Athyrium otophorum (Miq.) Koidz. | 4 |  |  |  | 106.3 | 1569.1 | 26.7 | 4.0 | 3 | N/B/S |
| Athyrium vidalii (Franch. & Sav.) Nakai | 53 |  |  |  | 360.7 | 55656.8 | 14.7 | 24.6 | 6 | W/R/S |
| Athyrium yokoscense (Franch. & Sav.) H.Christ | 320 |  |  |  | 468.5 | 97051.0 | 7.5 | 62.6 | 10 | W/B/L |
| Atractylodes ovata (Thunb.) DC. | 402 |  |  |  | 486.2 | 100414.8 | 7.0 | 69.6 | 8 | W/B/L |
| Atriplex gmelinii C.A.Mey. | 2 |  |  |  | 204.2 | NA | 204.2 | 1.0 | 1 | N/R/S |
| Atriplex subcordata Kitag. | 2 |  |  |  | 23.0 | NA | 23.0 | 1.0 | 2 | N/R/S |
| Aucuba japonica Thunb. | 4 |  |  |  | 228.3 | 55.1 | 57.1 | 4.0 | 2 | N/R/S |
| Avena fatua L. | 15 |  |  |  | 396.7 | 70864.6 | 52.2 | 7.6 | 6 | W/R/S |
| Avena sativa L. | 2 |  |  |  | 18.8 | NA | 18.8 | 1.0 | 2 | N/R/S |
| Barbarea orthoceras Ledeb. | 98 |  |  |  | 364.2 | 69091.7 | 12.9 | 28.2 | 10 | W/B/S |
| Barbarea vulgaris R.Br. | 16 |  |  |  | 271.3 | 30254.2 | 25.1 | 10.8 | 5 | N/B/L |
| Beckmannia syzigachne (Steud.) Fernald | 49 |  |  |  | 427.8 | 80689.6 | 21.9 | 19.5 | 9 | W/B/S |
| Belamcanda chinensis (L.) DC. | 10 |  |  | LC | 356.2 | 39325.2 | 62.9 | 5.7 | 8 | W/B/S |
| Berberis amurensis var. amurensis Rupr. | 14 |  |  |  | 245.9 | 25141.8 | 22.3 | 11.0 | 5 | N/B/L |
| Berberis koreana Palib. | 34 |  | V |  | 196.2 | 7322.3 | 4.4 | 44.3 | 6 | N/B/L |
| Berchemia berchemiifolia (Makino) Koidz. | 3 |  |  | LC | 96.4 | 828.4 | 45.7 | 2.1 | 2 | N/R/S |
| Berteroella maximowiczii (Palib.) O.E.Schulz | 72 |  |  |  | 378.7 | 73901.1 | 15.0 | 25.3 | 9 | W/B/S |
| Betula chinensis Maxim. | 33 |  |  |  | 342.9 | 42408.0 | 18.4 | 18.7 | 5 | W/R/S |
| Betula costata Trautv. | 19 |  |  |  | 335.1 | 21835.0 | 25.2 | 13.3 | 3 | W/R/S |
| Betula dahurica Pall. | 71 |  |  |  | 346.3 | 50937.5 | 13.7 | 25.3 | 8 | W/B/S |
| Betula ermanii Cham. | 12 |  |  |  | 279.9 | 29531.2 | 18.2 | 15.4 | 3 | N/B/L |
| Betula platyphylla var. japonica (Miq.) Hara | 63 |  |  |  | 462.1 | 73415.8 | 18.7 | 24.8 | 6 | W/R/S |
| Betula schmidtii Regel | 54 |  |  |  | 340.9 | 56895.8 | 18.1 | 18.8 | 6 | W/R/S |
| Bidens bipinnata L. | 215 |  |  |  | 472.4 | 99318.0 | 10.4 | 45.4 | 11 | W/B/L |
| Bidens biternata (Lour.) Merr. & Sherff ex Sherff | 37 |  |  |  | 360.2 | 61493.0 | 20.4 | 17.7 | 5 | W/R/S |
| Bidens cernua L. | 2 |  |  |  | 125.0 | NA | 125.0 | 1.0 | 1 | N/R/S |
| Bidens frondosa L. | 245 |  |  |  | 474.0 | 94574.4 | 10.1 | 46.8 | 9 | W/B/L |
| Bidens parviflora Willd. | 14 |  |  |  | 302.0 | 33673.8 | 22.7 | 13.3 | 4 | N/B/L |
| Bidens pilosa var. pilosa L. | 10 |  |  |  | 350.9 | 47935.2 | 74.3 | 4.7 | 4 | W/R/S |
| Bidens radiata var. pinnatifida (Turcz. ex DC.) Kitam. | 6 |  |  |  | 343.7 | 19690.4 | 41.5 | 8.3 | 4 | W/R/S |
| Bidens tripartita L. | 110 |  |  |  | 481.4 | 95099.9 | 13.3 | 36.1 | 8 | W/B/L |
| Bistorta pacifica (Petrov ex Kom.) Kom. | 2 |  |  |  | 203.3 | NA | 203.3 | 1.0 | 2 | N/R/S |
| Bletilla striata (Thunb. ex Murray) Rchb.f. | 3 |  |  | LC | 22.4 | 31.2 | 9.3 | 2.4 | 2 | N/R/S |
| Blyxa japonica (Miq.) Maxim. ex Asch. & Gurk. | 2 |  |  | NT | 0.0 | NA | 0.0 | 1.0 | 1 | N/R/S |
| Boehmeria longispica Steud. | 110 |  |  |  | 431.6 | 78152.6 | 12.5 | 34.5 | 7 | W/B/L |
| Boehmeria nivea (L.) Gaudich. | 45 |  |  |  | 448.9 | 65697.0 | 21.1 | 21.3 | 8 | W/B/S |
| Boehmeria pannosa Nakai & Satake | 7 |  |  |  | 227.8 | 16029.5 | 36.4 | 6.3 | 4 | N/B/L |
| Boehmeria platanifolia Franch. & Sav. | 131 |  |  |  | 420.7 | 79986.0 | 11.8 | 35.7 | 9 | W/B/L |
| Boehmeria sieboldiana Blume | 10 |  |  |  | 311.3 | 48591.5 | 44.5 | 7.0 | 4 | N/B/L |
| Boehmeria spicata (Thunb.) Thunb. | 494 |  |  |  | 490.4 | 97763.7 | 6.4 | 76.5 | 12 | W/B/L |
| Boehmeria tricuspis (Hance) Makino | 122 |  |  |  | 485.3 | 81784.5 | 10.2 | 47.6 | 6 | W/R/L |
| Boehmeria tricuspis var. unicuspis Makino | 66 |  |  |  | 427.1 | 78121.0 | 11.1 | 38.5 | 7 | W/B/L |
| Bothriochloa parviflora (R.Br.) Ohwi | 3 |  |  |  | 89.3 | 639.8 | 46.3 | 1.9 | 3 | N/B/S |
| Bothriospermum secundum Maxim. | 3 |  |  |  | 100.5 | 94.8 | 38.9 | 2.6 | 1 | N/R/S |
| Bothriospermum tenellum (Hornem.) Fisch. & C.A.Mey. | 43 |  |  |  | 397.2 | 71600.8 | 19.9 | 20.0 | 6 | W/R/S |
| Botrychium japonicum (Prantl) Underw. | 5 |  |  |  | 103.9 | 2942.1 | 34.1 | 3.1 | 3 | N/B/S |
| Botrychium strictum Underw. | 5 |  |  | NT | 218.6 | 15948.1 | 75.6 | 2.9 | 3 | N/B/S |
| Botrychium ternatum (Thunb.) Sw. | 96 |  |  |  | 428.6 | 84821.2 | 16.5 | 25.9 | 8 | W/B/S |
| Botrychium virginianum (L.) Sw. | 4 |  |  |  | 377.2 | 16150.3 | 108.6 | 3.5 | 2 | W/R/S |
| Brachybotrys paridiformis Maxim. ex Oliv. | 47 |  |  |  | 287.0 | 30361.7 | 13.5 | 21.3 | 4 | N/B/L |
| Brachyelytrum erectum var. japonicum Hack. | 9 |  |  |  | 249.1 | 18492.8 | 33.3 | 7.5 | 5 | N/B/L |
| Brachypodium sylvaticum (Huds.) P.Beauv. | 7 |  |  |  | 374.1 | 32021.3 | 65.0 | 5.8 | 4 | W/R/S |
| Brachypodium sylvaticum var. miserum (Thunb. ex Murray) Koidz. | 3 |  |  |  | 34.7 | 47.2 | 14.6 | 2.4 | 2 | N/R/S |
| Brassica juncea var. juncea (L.) Czern. | 39 |  |  |  | 358.1 | 59110.2 | 18.4 | 19.5 | 8 | W/B/S |
| Brassica napus L. | 33 |  |  |  | 391.2 | 51985.2 | 23.2 | 16.9 | 7 | W/B/S |
| Breea segeta for. segeta (Willd.) Kitam. | 90 |  |  |  | 422.6 | 88043.1 | 14.7 | 28.7 | 6 | W/R/L |
| Briza minor L. | 3 |  |  |  | 82.8 | 23.6 | 29.2 | 2.8 | 3 | N/B/S |
| Bromus canadensis subsp. yezoensis (Ohwi) V.N.Voroshilov | 7 |  |  |  | 360.9 | 44069.3 | 96.4 | 3.7 | 3 | W/R/S |
| Bromus catharticus Vahl | 8 |  |  |  | 272.8 | 19689.1 | 29.9 | 9.1 | 4 | N/B/L |
| Bromus inermis Leyss. | 4 |  |  |  | 226.8 | 11308.9 | 81.2 | 2.8 | 3 | N/B/S |
| Bromus japonicus Thunb. ex Murray | 138 |  |  |  | 462.3 | 88350.2 | 13.5 | 34.3 | 10 | W/B/L |
| Bromus mollis L. | 4 |  |  |  | 329.4 | 14653.3 | 96.6 | 3.4 | 3 | W/R/S |
| Bromus pauciflorus (Thunb.) Hack. | 21 |  |  |  | 364.0 | 39837.2 | 25.0 | 14.6 | 5 | W/R/S |
| Bromus rigidus Roth | 3 |  |  |  | 166.3 | 445.5 | 68.3 | 2.4 | 1 | N/R/S |
| Bromus secalinus L. | 2 |  |  |  | 173.2 | NA | 173.2 | 1.0 | 2 | N/R/S |
| Bromus sterilis L. | 2 |  |  |  | 213.5 | NA | 213.5 | 1.0 | 2 | N/R/S |
| Bromus tectorum var. tectorum L. | 22 |  |  |  | 462.9 | 65622.5 | 37.7 | 12.3 | 6 | W/R/S |
| Broussonetia kazinoki Siebold | 76 |  |  |  | 388.3 | 62252.0 | 13.7 | 28.4 | 7 | W/B/S |
| Broussonetia papyrifera L. L Her. ex Vent. | 9 |  |  |  | 316.5 | 44431.5 | 48.7 | 6.5 | 4 | W/R/S |
| Bulbostylis barbata (Rottb.) Kunth | 9 |  |  |  | 358.2 | 34592.1 | 57.8 | 6.2 | 4 | W/R/S |
| Bulbostylis densa (Wall.) Hand.-Mazz. | 12 |  |  |  | 285.6 | 30633.9 | 37.1 | 7.7 | 3 | N/B/L |
| Bupleurum falcatum L. | 48 |  |  |  | 436.3 | 66538.9 | 18.8 | 23.2 | 7 | W/B/S |
| Bupleurum longeradiatum Turcz. | 42 |  |  |  | 442.6 | 59544.3 | 16.4 | 27.0 | 3 | W/R/S |
| Buxus koreana Nakai ex Chung & al. | 61 |  |  |  | 430.0 | 73882.5 | 15.3 | 28.2 | 9 | W/B/S |
| Caesalpinia decapetala (Roth) Alston | 4 |  |  |  | 134.7 | 1738.8 | 37.4 | 3.6 | 2 | N/R/S |
| Calamagrostis arundinacea (L.) Roth | 264 |  |  |  | 477.7 | 99847.9 | 9.2 | 52.2 | 10 | W/B/L |
| Calamagrostis epigeios (L.) Roth | 67 |  |  |  | 414.1 | 86057.1 | 18.2 | 22.8 | 7 | W/B/S |
| Calamagrostis langsdorfii (Link) Trin. | 22 |  |  |  | 342.5 | 58260.4 | 28.8 | 11.9 | 3 | W/R/S |
| Calamagrostis pseudophragmites (Haller f.) Koeler | 6 |  |  |  | 389.6 | 24502.8 | 76.6 | 5.1 | 4 | W/R/S |
| Calendula officinalis L. | 2 |  |  |  | 160.6 | NA | 160.6 | 1.0 | 2 | N/R/S |
| Callicarpa dichotoma (Lour.) K.Koch | 43 |  |  |  | 382.7 | 69333.2 | 21.8 | 17.6 | 4 | W/R/S |
| Callicarpa japonica Thunb. | 430 |  |  |  | 491.4 | 103600.1 | 6.9 | 71.4 | 10 | W/B/L |
| Callicarpa japonica var. glabra Nakai | 2 |  |  |  | 10.6 | NA | 10.6 | 1.0 | 1 | N/R/S |
| Callicarpa mollis Siebold & Zucc. | 18 |  |  |  | 160.9 | 12384.4 | 8.3 | 19.3 | 4 | N/B/L |
| Callistephus chinensis (L.) Nees | 2 |  |  |  | 145.9 | NA | 145.9 | 1.0 | 1 | N/R/S |
| Callitriche palustris L. | 33 |  |  |  | 365.6 | 54993.4 | 20.5 | 17.8 | 4 | W/R/S |
| Calystegia dahurica (Herb.) Choisy | 4 |  |  |  | 31.9 | 318.4 | 15.5 | 2.1 | 2 | N/R/S |
| Calystegia hederacea Wall. | 51 |  |  |  | 463.7 | 63592.2 | 18.1 | 25.7 | 8 | W/B/S |
| Calystegia sepium for. sepium (L.) R.Br. | 16 |  |  |  | 335.2 | 51726.8 | 25.4 | 13.2 | 6 | W/R/S |
| Calystegia sepium var. japonicum (Choisy) Makino | 174 |  |  |  | 473.4 | 100410.0 | 12.2 | 38.7 | 10 | W/B/L |
| Calystegia soldanella (L.) Roem. & Schultb. | 19 |  |  |  | 469.6 | 71847.9 | 31.8 | 14.8 | 8 | W/B/S |
| Camellia japonica for. albipetala H.D.Chang | 3 |  | V |  | 20.6 | 45.6 | 9.6 | 2.1 | 2 | N/R/S |
| Camellia japonica L. | 40 |  |  |  | 440.3 | 61235.8 | 18.3 | 24.0 | 7 | W/B/S |
| Camellia sinensis L. | 8 |  |  |  | 140.3 | 8292.2 | 24.6 | 5.7 | 5 | N/B/S |
| Campanula glomerata var. dahurica Fisch. ex KerGawl. | 10 |  |  |  | 223.7 | 22453.1 | 48.5 | 4.6 | 3 | N/B/S |
| Campanula punctata Lam. | 128 |  |  |  | 342.7 | 64967.5 | 11.2 | 30.5 | 8 | W/B/L |
| Campanula takesimana Nakai | 2 |  | V |  | 19.8 | NA | 19.8 | 1.0 | 1 | N/R/S |
| Campsis grandifolia (Thunb.) K.Schum. | 8 |  |  |  | 312.2 | 29482.5 | 54.6 | 5.7 | 4 | N/B/S |
| Campylotropis macrocarpa (Bunge) Rehder | 9 |  |  |  | 219.2 | 24358.5 | 51.2 | 4.3 | 4 | N/B/S |
| Capsella burapastoris (L.) L.W.Medicus | 408 |  |  |  | 477.9 | 102978.1 | 8.2 | 58.2 | 12 | W/B/L |
| Capsicum annuum L. | 3 |  |  |  | 13.6 | 39.3 | 8.4 | 1.6 | 3 | N/B/S |
| Caragana microphylla Lam. | 2 |  |  |  | 195.2 | NA | 195.2 | 1.0 | 1 | N/R/S |
| Caragana sinica (Buc hoz) Rehder | 7 |  |  |  | 239.1 | 15232.1 | 53.6 | 4.5 | 4 | N/B/S |
| Cardamine amaraeformis Nakai | 4 |  |  |  | 277.3 | 19583.8 | 101.3 | 2.7 | 4 | N/B/S |
| Cardamine fallax L. | 135 |  |  |  | 422.2 | 87687.8 | 12.7 | 33.3 | 8 | W/B/L |
| Cardamine flexuosa With. | 335 |  |  |  | 488.0 | 104305.0 | 8.5 | 57.1 | 8 | W/B/L |
| Cardamine impatiens L. | 110 |  |  |  | 376.7 | 69627.3 | 12.0 | 31.4 | 9 | W/B/L |
| Cardamine komarovii var. komarovii Nakai | 34 |  |  |  | 168.3 | 12271.2 | 7.3 | 23.0 | 3 | N/B/L |
| Cardamine leucantha var. leucantha (Tausch) O.E.Schulz | 299 |  |  |  | 429.3 | 90079.1 | 8.0 | 53.9 | 11 | W/B/L |
| Cardamine lyrata Bunge | 19 |  |  |  | 331.3 | 41563.3 | 19.0 | 17.4 | 5 | W/R/S |
| Cardamine scutata Thunb. | 19 |  |  |  | 393.9 | 45212.0 | 32.6 | 12.1 | 5 | W/R/S |
| Carduus crispus L. | 167 |  |  |  | 454.6 | 77950.7 | 10.1 | 44.9 | 9 | W/B/L |
| Carex alterniflora Franch. | 16 |  |  |  | 363.4 | 58178.6 | 41.8 | 8.7 | 5 | W/R/S |
| Carex aphanolepis Franch. & Sav. | 11 |  |  |  | 320.1 | 37624.3 | 45.5 | 7.0 | 6 | W/R/S |
| Carex arenicola F.Schmidt | 3 |  |  |  | 34.6 | 19.7 | 14.7 | 2.3 | 2 | N/R/S |
| Carex biwensis Franch. | 7 |  |  |  | 334.1 | 32159.4 | 56.8 | 5.9 | 3 | W/R/S |
| Carex blepharicarpa var. stenocarpa Ohwi | 6 |  | V |  | 317.0 | 12821.8 | 67.9 | 4.7 | 3 | W/R/S |
| Carex bostrychostigma Maxim. | 93 |  |  |  | 415.4 | 85710.8 | 15.0 | 27.7 | 9 | W/B/S |
| Carex breviculmis R.Br. | 138 |  |  |  | 429.5 | 85889.2 | 11.3 | 38.2 | 11 | W/B/L |
| Carex brownii Tuck. | 2 |  |  |  | 23.4 | NA | 23.4 | 1.0 | 2 | N/R/S |
| Carex capillacea Boott | 3 |  |  |  | 365.7 | 1799.4 | 136.4 | 2.7 | 2 | W/R/S |
| Carex ciliatomarginata Nakai | 61 |  |  |  | 420.6 | 66016.5 | 16.4 | 25.6 | 6 | W/R/S |
| Carex dickinsii Franch. & Sav. | 14 |  |  |  | 438.8 | 49207.5 | 35.9 | 12.2 | 4 | W/R/S |
| Carex dimorpholepis Steud. | 31 |  |  |  | 436.4 | 61690.9 | 27.3 | 16.0 | 5 | W/R/S |
| Carex dispalata var. dispalata Boott | 46 |  |  |  | 424.4 | 70543.8 | 20.2 | 21.0 | 8 | W/B/S |
| Carex doniana Spreng. | 18 |  |  |  | 389.0 | 52571.8 | 33.8 | 11.5 | 4 | W/R/S |
| Carex fernaldiana H.Lev. & Vaniot | 25 |  |  |  | 230.0 | 21792.2 | 9.6 | 24.0 | 4 | N/B/L |
| Carex forficula var. forficula Franch. & Sav. | 44 |  |  |  | 404.2 | 65645.9 | 19.4 | 20.8 | 7 | W/B/S |
| Carex gibba Wahlenb. | 15 |  |  |  | 282.1 | 32169.3 | 37.1 | 7.6 | 6 | N/B/L |
| Carex gifuensis Franch. | 18 |  |  |  | 239.7 | 32218.8 | 22.5 | 10.6 | 5 | N/B/L |
| Carex glabrescens Ohwi | 13 |  |  |  | 362.1 | 22751.1 | 37.7 | 9.6 | 5 | W/R/S |
| Carex hakonensis Franch. & Sav. | 3 |  |  |  | 261.2 | 15679.7 | 170.3 | 1.5 | 2 | N/R/S |
| Carex heterolepis Bunge | 26 |  |  |  | 395.1 | 58838.5 | 26.8 | 14.7 | 5 | W/R/S |
| Carex hondoensis Ohwi | 2 |  |  |  | 159.9 | NA | 159.9 | 1.0 | 2 | N/R/S |
| Carex humilis var. nana (H.Lev. & Vaniot) Ohwi | 111 |  |  |  | 432.6 | 78198.8 | 12.3 | 35.3 | 9 | W/B/L |
| Carex idzuroei Franch. & Sav. | 4 |  |  |  | 105.8 | 2432.0 | 39.4 | 2.7 | 4 | N/B/S |
| Carex jaluensis Kom. | 8 |  |  |  | 275.7 | 21066.2 | 60.3 | 4.6 | 3 | N/B/S |
| Carex japonica Thunb. | 134 |  |  |  | 424.2 | 77073.7 | 12.8 | 33.2 | 11 | W/B/L |
| Carex kobomugi Ohwi | 3 |  |  |  | 296.1 | 13565.4 | 160.8 | 1.8 | 3 | N/B/S |
| Carex laevissima Nakai | 29 |  |  |  | 407.1 | 65972.7 | 28.3 | 14.4 | 5 | W/R/S |
| Carex lanceolata Boott | 233 |  |  |  | 449.6 | 93816.5 | 9.0 | 49.9 | 10 | W/B/L |
| Carex lasiolepis Franch. | 4 |  |  |  | 359.7 | 23041.9 | 103.0 | 3.5 | 3 | W/R/S |
| Carex leiorhyncha C.A.Mey. | 163 |  |  |  | 402.1 | 84918.4 | 13.1 | 30.7 | 11 | W/B/L |
| Carex lenta D.Don | 11 |  |  |  | 371.1 | 26710.7 | 48.0 | 7.7 | 4 | W/R/S |
| Carex leucochlora Bunge | 4 |  |  |  | 188.4 | 1665.4 | 54.5 | 3.5 | 3 | N/B/S |
| Carex maackii Maxim. | 10 |  |  |  | 359.6 | 18345.9 | 41.4 | 8.7 | 4 | W/R/S |
| Carex maximowiczii var. maximowiczii Miq. | 40 |  |  |  | 390.4 | 61075.9 | 20.3 | 19.2 | 6 | W/R/S |
| Carex mira Kuk. | 2 |  |  |  | 12.5 | NA | 12.5 | 1.0 | 2 | N/R/S |
| Carex mitrata var. mitrata Franch. | 8 |  |  |  | 117.4 | 3654.7 | 19.2 | 6.1 | 3 | N/B/S |
| Carex mollicula Boott | 5 |  |  |  | 268.2 | 7443.4 | 59.1 | 4.5 | 3 | N/B/S |
| Carex nervata Franch. & Sav. | 4 |  |  |  | 136.0 | 6934.9 | 61.7 | 2.2 | 3 | N/B/S |
| Carex neurocarpa Maxim. | 117 |  |  |  | 456.8 | 83927.4 | 12.6 | 36.3 | 9 | W/B/L |
| Carex norvegica Retz. | 5 |  |  |  | 241.3 | 13618.9 | 52.4 | 4.6 | 4 | N/B/S |
| Carex okamotoi Ohwi | 29 |  | V |  | 372.9 | 55756.8 | 17.2 | 21.6 | 3 | W/R/S |
| Carex onoei Franch. & Sav. | 15 |  |  |  | 313.9 | 42272.4 | 40.0 | 7.8 | 5 | N/B/L |
| Carex oxyandra Kudo | 4 |  |  |  | 203.8 | 11224.3 | 78.2 | 2.6 | 3 | N/B/S |
| Carex papulosa Boott | 3 |  |  |  | 122.7 | 2696.0 | 70.5 | 1.7 | 2 | N/R/S |
| Carex parciflora var. macroglossa (Franch. & Sav.) Ohwi | 3 |  |  |  | 250.8 | 1.7 | 84.0 | 3.0 | 2 | N/R/S |
| Carex pediformis C.A.Mey. | 13 |  |  |  | 233.3 | 18958.5 | 27.7 | 8.4 | 5 | N/B/L |
| Carex peiktusani Kom. | 2 |  |  |  | 0.2 | NA | 0.2 | 1.0 | 2 | N/R/S |
| Carex phacota Spreng. | 8 |  |  |  | 341.7 | 27491.2 | 45.0 | 7.6 | 4 | W/R/S |
| Carex planiculmis Kom. | 8 |  |  |  | 175.8 | 5966.7 | 20.7 | 8.5 | 4 | N/B/L |
| Carex poculisquama Kuk | 2 |  |  |  | 9.2 | NA | 9.2 | 1.0 | 2 | N/R/S |
| Carex polyschoena H.Lev. & Vaniot | 66 |  |  |  | 400.9 | 62971.9 | 16.2 | 24.7 | 5 | W/R/S |
| Carex pseudochinensis H.Lev. & Vaniot | 2 |  | V | LC | 269.3 | NA | 269.3 | 1.0 | 2 | N/R/S |
| Carex pumila Thunb. | 7 |  |  |  | 359.4 | 16610.9 | 28.5 | 12.6 | 3 | W/R/S |
| Carex sabynensis Less. | 50 |  |  |  | 341.1 | 56294.3 | 20.2 | 16.8 | 5 | W/R/S |
| Carex schmidtii Meinsh. | 3 |  |  |  | 209.8 | 196.0 | 73.1 | 2.9 | 3 | N/B/S |
| Carex shimidzensis Franch. | 7 |  |  |  | 176.8 | 10741.9 | 39.3 | 4.5 | 5 | N/B/S |
| Carex siderosticta Hance | 312 |  |  |  | 440.8 | 95568.0 | 8.0 | 55.1 | 11 | W/B/L |
| Carex thunbergii Steud. | 40 |  |  |  | 382.3 | 78517.3 | 21.0 | 18.2 | 8 | W/B/S |
| Carex transversa Boott | 10 |  |  |  | 332.9 | 26954.9 | 41.2 | 8.1 | 4 | W/R/S |
| Carex tristachya var. tristachya Thunb. | 12 |  |  |  | 264.5 | 14068.7 | 29.0 | 9.1 | 5 | N/B/L |
| Carex ussuriensis Kom. | 2 |  |  |  | 133.6 | NA | 133.6 | 1.0 | 1 | N/R/S |
| Carex vesicaria L. | 3 |  |  |  | 370.3 | 14505.0 | 171.8 | 2.2 | 1 | W/R/S |
| Carpesium abrotanoides L. | 88 |  |  |  | 433.7 | 84516.5 | 12.2 | 35.7 | 6 | W/R/L |
| Carpesium cernuum L. | 52 |  |  |  | 433.8 | 75318.3 | 18.8 | 23.1 | 5 | W/R/S |
| Carpesium divaricatum Siebold & Zucc. | 111 |  |  |  | 380.0 | 79162.1 | 12.4 | 30.7 | 8 | W/B/L |
| Carpesium glossophyllum Maxim. | 8 |  |  |  | 314.7 | 16000.8 | 41.6 | 7.6 | 3 | N/B/L |
| Carpesium macrocephalum Franch. & Sav. | 17 |  |  |  | 308.7 | 50359.2 | 38.7 | 8.0 | 5 | N/B/L |
| Carpesium triste Maxim. | 36 |  |  |  | 314.7 | 32741.6 | 15.2 | 20.7 | 5 | N/B/L |
| Carpinus cordata Blume | 120 |  |  |  | 440.0 | 78762.4 | 11.1 | 39.8 | 8 | W/B/L |
| Carpinus laxiflora var. laxiflora (Siebold & Zucc.) Blume | 126 |  |  |  | 442.8 | 89436.4 | 10.5 | 42.3 | 8 | W/B/L |
| Carpinus tschonoskii var. tschonoskii Maxim. | 39 |  |  |  | 346.2 | 48134.9 | 18.7 | 18.5 | 5 | W/R/S |
| Carpinus turczaninovii Hance | 62 |  |  |  | 370.3 | 72040.2 | 9.0 | 41.1 | 6 | W/R/L |
| Carpinus turczaninovii var. arguta Uyeki | 4 |  |  |  | 14.7 | 45.6 | 5.5 | 2.7 | 3 | N/B/S |
| Caryopteris divaricata (Siebold & Zucc.) Maxim. | 42 |  |  |  | 463.9 | 73104.8 | 21.1 | 22.0 | 8 | W/B/S |
| Caryopteris incana (Thunb.) Miq. | 49 |  |  |  | 442.9 | 59880.6 | 16.4 | 27.0 | 7 | W/B/S |
| Cassia tora L. | 7 |  |  |  | 212.4 | 17478.8 | 38.7 | 5.5 | 3 | N/B/S |
| Castanea bungeana Blume | 9 |  |  |  | 308.3 | 15017.7 | 31.5 | 9.8 | 4 | N/B/L |
| Castanea crenata Siebold & Zucc. | 401 |  |  |  | 475.5 | 101890.8 | 7.4 | 63.9 | 10 | W/B/L |
| Castanopsis sieboldii (Makino) Hatus. | 2 |  |  |  | 136.5 | NA | 136.5 | 1.0 | 2 | N/R/S |
| Catalpa ovata G.Don | 8 |  |  |  | 203.1 | 19294.9 | 36.8 | 5.5 | 5 | N/B/S |
| Caulophyllum robustum Maxim. | 48 |  |  |  | 357.0 | 57342.2 | 15.4 | 23.2 | 3 | W/R/S |
| Cayratia japonica (Thunb.) Gagnep. | 23 |  |  |  | 359.1 | 59525.3 | 32.7 | 11.0 | 5 | W/R/S |
| Cedrela sinensis Juss. | 5 |  |  |  | 257.1 | 22710.0 | 99.4 | 2.6 | 3 | N/B/S |
| Cedrus deodara (Roxb.) Loudon | 3 |  |  |  | 207.8 | 245.0 | 73.8 | 2.8 | 3 | N/B/S |
| Celastrus flagellaris Rupr. | 54 |  |  |  | 387.8 | 61538.0 | 16.0 | 24.3 | 6 | W/R/S |
| Celastrus orbiculatus Thunb. | 392 |  |  |  | 487.9 | 103966.6 | 7.1 | 68.5 | 12 | W/B/L |
| Celastrus stephanotifolius Makino | 10 |  |  |  | 281.3 | 22098.5 | 32.7 | 8.6 | 2 | N/R/L |
| Celosia argentea L. | 2 |  |  |  | 96.3 | NA | 96.3 | 1.0 | 1 | N/R/S |
| Celosia cristata L. | 2 |  |  |  | 62.6 | NA | 62.6 | 1.0 | 1 | N/R/S |
| Celtis aurantiaca Nakai | 21 |  |  |  | 249.4 | 17217.7 | 18.5 | 13.5 | 4 | N/B/L |
| Celtis biondii var. heterophylla (H.Lev.) Schneid | 29 |  |  |  | 147.8 | 10061.6 | 8.2 | 17.9 | 4 | N/B/L |
| Celtis bungeana Blume | 2 |  |  |  | 178.3 | NA | 178.3 | 1.0 | 1 | N/R/S |
| Celtis choseniana Nakai | 4 |  | V |  | 236.4 | 25416.8 | 141.7 | 1.7 | 3 | N/B/S |
| Celtis jessoensis Koidz. | 9 |  |  |  | 322.6 | 31718.0 | 48.9 | 6.6 | 3 | W/R/S |
| Celtis koraiensis Nakai | 14 |  |  |  | 149.1 | 7346.5 | 9.9 | 15.0 | 6 | N/B/L |
| Celtis sinensis Pers. | 137 |  |  |  | 430.9 | 81668.2 | 11.8 | 36.7 | 10 | W/B/L |
| Centaurea cyanus L. | 4 |  |  |  | 0.8 | 0.0 | 0.2 | 3.7 | 2 | N/R/S |
| Centella asiatica (L.) Urb. | 4 |  |  |  | 318.9 | 2582.1 | 88.9 | 3.6 | 3 | W/R/S |
| Centipeda minima (L.) A.Br. & Asch. | 93 |  |  |  | 375.2 | 71631.4 | 14.2 | 26.3 | 9 | W/B/S |
| Cephalanthera erecta for. subaphylla Hiroe | 2 |  |  | VU | 239.9 | NA | 239.9 | 1.0 | 1 | N/R/S |
| Cephalanthera ereta (Thunb. ex Murray) Blume | 80 |  |  |  | 418.7 | 85822.0 | 14.8 | 28.2 | 5 | W/R/S |
| Cephalanthera falcata (Thunb. ex A.Murray) Blume | 25 |  |  |  | 297.7 | 27278.3 | 16.9 | 17.6 | 4 | N/B/L |
| Cephalanthera longibracteata Blume | 305 |  |  |  | 454.2 | 90661.4 | 7.7 | 59.0 | 10 | W/B/L |
| Cephalotaxus harringtonia (Knight) K.Koch | 47 |  |  |  | 369.8 | 40759.0 | 12.1 | 30.7 | 6 | W/R/L |
| Cerastium fischerianum Ser. | 10 |  |  |  | 288.2 | 30330.5 | 14.0 | 20.6 | 3 | N/B/L |
| Cerastium glomeratum Thuill. | 21 |  |  |  | 425.7 | 59164.4 | 29.2 | 14.6 | 6 | W/R/S |
| Cerastium holosteoides var. hallaisanense (Nakai) Mizush. | 296 |  |  |  | 445.4 | 96601.7 | 8.9 | 49.9 | 12 | W/B/L |
| Ceratophyllum demersum var. demersum L. | 10 |  |  |  | 256.4 | 21548.7 | 27.8 | 9.2 | 5 | N/B/L |
| Cercis chinensis Bunge | 10 |  |  |  | 322.4 | 25705.0 | 43.2 | 7.5 | 4 | W/R/S |
| Chaenomeles sinensis (Thouin) Koehne | 4 |  |  |  | 221.2 | 11556.3 | 78.2 | 2.8 | 3 | N/B/S |
| Chaenomeles speciosa (Sweet) Nakai | 8 |  |  |  | 321.6 | 28884.6 | 62.6 | 5.1 | 3 | W/R/S |
| Chamaecrista nomame (Siebold) H.Ohashi | 270 |  |  |  | 457.0 | 96811.3 | 9.5 | 48.2 | 10 | W/B/L |
| Chamaecyparis obtusa (Siebold & Zucc.) Endl. | 48 |  |  |  | 267.7 | 31320.4 | 13.3 | 20.1 | 7 | N/B/L |
| Chamaecyparis pisifera (Siebold & Zucc.) Endl. | 7 |  |  |  | 196.9 | 4741.1 | 23.7 | 8.3 | 3 | N/B/L |
| Chamerion angustifolium (L.) Holub | 4 |  |  | VU | 143.6 | 737.4 | 10.3 | 14.0 | 3 | N/B/L |
| Cheilanthes argentea (Gmel.) G.Kunze | 11 |  |  |  | 255.8 | 17092.2 | 29.1 | 8.8 | 4 | N/B/L |
| Chelidonium majus var. asiaticum (Hara) Ohwi | 437 |  |  |  | 480.9 | 98176.4 | 7.7 | 62.5 | 12 | W/B/L |
| Chenopodium acuminatum Willd. | 4 |  |  |  | 438.0 | 29396.5 | 150.9 | 2.9 | 3 | W/R/S |
| Chenopodium album var. album L. | 23 |  |  |  | 360.8 | 50431.9 | 26.4 | 13.7 | 6 | W/R/S |
| Chenopodium album var. centrorubrum Makino | 224 |  |  |  | 463.2 | 97143.1 | 10.1 | 46.1 | 11 | W/B/L |
| Chenopodium ambrosioides L. | 6 |  |  |  | 416.4 | 43546.8 | 98.5 | 4.2 | 3 | W/R/S |
| Chenopodium bryoniaefolium Bunge | 6 |  |  |  | 349.5 | 41230.4 | 86.4 | 4.0 | 2 | W/R/S |
| Chenopodium ficifolium Smith | 95 |  |  |  | 441.9 | 91069.4 | 15.7 | 28.2 | 7 | W/B/S |
| Chenopodium glaucum L. | 25 |  |  |  | 378.5 | 75645.4 | 35.3 | 10.7 | 8 | W/B/S |
| Chenopodium koraiense Nakai | 3 |  |  |  | 341.1 | 19684.9 | 205.8 | 1.7 | 3 | W/R/S |
| Chenopodium virgatum Thunb. | 2 |  |  |  | 162.8 | NA | 162.8 | 1.0 | 2 | N/R/S |
| Chimaphila japonica Miq. | 112 |  |  |  | 447.4 | 76165.4 | 11.9 | 37.7 | 6 | W/R/L |
| Chionanthus retusus Lindl. & Paxton | 17 |  |  | LC | 396.9 | 58525.7 | 42.0 | 9.4 | 5 | W/R/S |
| Chloranthus fortunei (A.Gray) Solms | 22 |  |  |  | 279.9 | 31379.8 | 13.2 | 21.2 | 5 | N/B/L |
| Chloranthus japonicus Siebold | 272 |  |  |  | 464.2 | 97642.9 | 8.1 | 57.0 | 9 | W/B/L |
| Chloris virgata Sw. | 7 |  |  |  | 300.8 | 37154.3 | 50.7 | 5.9 | 4 | N/B/S |
| Chorispora tenella DC. | 3 |  |  |  | 212.3 | 1377.3 | 85.3 | 2.5 | 2 | N/R/S |
| Chrysanthemum coronarium L. | 4 |  |  |  | 178.1 | 3264.3 | 62.9 | 2.8 | 3 | N/B/S |
| Chrysanthemum leucanthemum L. | 2 |  |  |  | 49.7 | NA | 49.7 | 1.0 | 2 | N/R/S |
| Chrysanthemum zawadskii subsp. naktongense (Nakai) Y.N.Lee | 4 |  |  |  | 227.7 | 13438.1 | 97.5 | 2.3 | 2 | N/R/S |
| Chrysosplenium barbatum Nakai | 22 |  | V |  | 349.6 | 51969.0 | 23.1 | 15.2 | 4 | W/R/S |
| Chrysosplenium flagelliferum F.Schmidt | 79 |  |  |  | 354.8 | 56637.1 | 12.7 | 27.9 | 8 | W/B/S |
| Chrysosplenium flaviflorum Ohwi | 3 |  | V |  | 106.2 | 6.6 | 35.5 | 3.0 | 2 | N/R/S |
| Chrysosplenium grayanum Maxim. | 65 |  |  |  | 393.1 | 69047.9 | 15.7 | 25.0 | 7 | W/B/S |
| Chrysosplenium japonicum (Maxim.) Makino | 27 |  |  |  | 267.1 | 38204.6 | 17.8 | 15.0 | 5 | N/B/L |
| Chrysosplenium macrostemon Maxim. ex Franch. & Sav. | 9 |  |  |  | 334.6 | 27767.4 | 29.8 | 11.2 | 3 | W/R/S |
| Chrysosplenium pilosum Maxim. | 8 |  |  |  | 376.0 | 27656.9 | 53.7 | 7.0 | 1 | W/R/S |
| Chrysosplenium pilosum var. fulvum (N.Terracc.) H.Hara | 3 |  |  |  | 157.8 | 7284.1 | 119.3 | 1.3 | 1 | N/R/S |
| Chrysosplenium pilosum var. valdepiosum Ohwi | 12 |  |  |  | 290.5 | 30674.5 | 28.5 | 10.2 | 3 | N/B/L |
| Chrysosplenium pseudofauriei H.Lev. | 25 |  |  |  | 301.3 | 27224.5 | 21.2 | 14.2 | 4 | N/B/L |
| Chrysosplenium ramosum Maxim. | 8 |  |  |  | 96.2 | 2351.7 | 21.5 | 4.5 | 3 | N/B/S |
| Cimicifuga dahurica (Turcz. ex Fisch. & C.A.Mey.) Maxim. | 71 |  |  |  | 370.8 | 52200.5 | 11.8 | 31.5 | 5 | W/R/L |
| Cimicifuga foetida L. | 20 |  |  |  | 222.6 | 22375.9 | 14.5 | 15.4 | 3 | N/B/L |
| Cimicifuga heracleifolia var. bifida Nakai | 10 |  | V |  | 140.5 | 7225.8 | 19.2 | 7.3 | 5 | N/B/L |
| Cimicifuga heracleifolia var. heracleifolia Kom. | 44 |  |  |  | 332.4 | 48858.2 | 12.3 | 27.0 | 6 | W/R/S |
| Cimicifuga japonica (Thunb.) Spreng. | 3 |  |  |  | 290.0 | 1279.8 | 105.3 | 2.8 | 2 | N/R/S |
| Cimicifuga simplex (DC.) Turcz. | 27 |  |  |  | 348.5 | 41295.4 | 15.3 | 22.7 | 6 | W/R/S |
| Circaea alpina L. | 11 |  |  |  | 296.7 | 35261.7 | 44.8 | 6.6 | 3 | N/B/L |
| Circaea cordata Royle | 46 |  |  |  | 442.2 | 69765.5 | 21.6 | 20.5 | 6 | W/R/S |
| Circaea lutetiana subsp. Quadrisulcata L. (Maxim.) Asch. & Magnus | 20 |  |  |  | 315.0 | 47794.0 | 30.5 | 10.3 | 3 | N/B/L |
| Circaea mollis Slebold & Zucc. | 84 |  |  |  | 406.5 | 82021.0 | 15.0 | 27.0 | 7 | W/B/S |
| Cirsium chanroenicum (L.) Nakai | 25 |  |  |  | 260.5 | 30246.4 | 23.4 | 11.1 | 5 | N/B/L |
| Cirsium japonicum for. nakaianum (H.Lev. & Vaniot) W.T.Lee | 3 |  |  |  | 153.5 | 3166.0 | 77.1 | 2.0 | 3 | N/B/S |
| Cirsium japonicum var. maackii (Maxim.) Matsum. | 437 |  |  |  | 485.1 | 103602.6 | 6.9 | 70.1 | 10 | W/B/L |
| Cirsium japonicum var. spinossimum Kitam. | 2 |  |  |  | 117.4 | NA | 117.4 | 1.0 | 2 | N/R/S |
| Cirsium lineare (Thunb.) Sch.Bip. | 3 |  |  |  | 34.7 | 47.6 | 14.7 | 2.4 | 2 | N/R/S |
| Cirsium pendulum Fisch. ex DC. | 63 |  |  |  | 425.7 | 76700.5 | 17.3 | 24.7 | 5 | W/R/S |
| Cirsium schantarense Trautv. & Mey. | 17 |  |  |  | 379.5 | 62992.3 | 42.6 | 8.9 | 6 | W/R/S |
| Cirsium setidens (Dunn) Nakai | 127 |  | V |  | 458.8 | 81875.1 | 10.5 | 43.9 | 8 | W/B/L |
| Cirsium setidens for. alba T.B.Lee | 3 |  | V |  | 86.9 | 307.6 | 34.3 | 2.5 | 2 | N/R/S |
| Cirsium toraiense Nakai ex Kitam. | 3 |  |  |  | 203.8 | 9443.5 | 130.0 | 1.6 | 2 | N/R/S |
| Cirsium vlassovianum Fisch. ex DC. | 3 |  |  |  | 149.1 | 1804.7 | 64.5 | 2.3 | 2 | N/R/S |
| Clausia trichosepala (Turcz.) Dvorak | 3 |  |  |  | 324.1 | 21382.8 | 204.9 | 1.6 | 2 | W/R/S |
| Cleistogenes hackelii (Honda) Honda | 24 |  |  |  | 404.0 | 57170.3 | 23.2 | 17.4 | 5 | W/R/S |
| Clematis apiifolia DC. | 496 |  |  |  | 475.7 | 100779.1 | 7.0 | 67.7 | 12 | W/B/L |
| Clematis brachyura Maxim. | 55 |  | V |  | 425.0 | 73825.6 | 20.2 | 21.0 | 6 | W/R/S |
| Clematis brevicaudata DC. | 3 |  |  |  | 179.1 | 12147.5 | 161.7 | 1.1 | 3 | N/B/S |
| Clematis fusca Turcz. | 10 |  |  |  | 237.5 | 17600.1 | 33.8 | 7.0 | 3 | N/B/L |
| Clematis fusca var. coreana (H.Lev. & Vaniot) Nakai | 13 |  | V |  | 192.2 | 15066.9 | 28.7 | 6.7 | 4 | N/B/L |
| Clematis fusca var. violacea Maxim. | 39 |  |  |  | 326.1 | 39173.4 | 18.1 | 18.1 | 7 | W/B/S |
| Clematis heracleifolia DC. | 147 |  |  |  | 371.2 | 62870.7 | 8.4 | 44.2 | 10 | W/B/L |
| Clematis heracleifolia var. davidiana Hemsl. | 10 |  |  |  | 189.7 | 18538.7 | 36.0 | 5.3 | 4 | N/B/S |
| Clematis koreana Kom. | 23 |  |  |  | 234.1 | 22833.4 | 13.2 | 17.7 | 5 | N/B/L |
| Clematis patens C.Morren & Decne. | 134 |  |  |  | 405.7 | 91887.7 | 13.3 | 30.6 | 8 | W/B/L |
| Clematis serratifolia Rehder | 11 |  |  |  | 61.7 | 1554.4 | 12.1 | 5.1 | 4 | N/B/S |
| Clematis terniflora DC. | 59 |  |  |  | 448.4 | 90465.5 | 16.7 | 26.8 | 7 | W/B/S |
| Clematis terniflora var. mandshurica (Rupr.) Ohwi | 262 |  |  |  | 451.0 | 96422.0 | 8.7 | 51.8 | 10 | W/B/L |
| Clematis trichotoma Nakai | 174 |  | V |  | 467.7 | 90996.8 | 10.6 | 44.1 | 8 | W/B/L |
| Clerodendrum trichotomum Thunb. | 380 |  |  |  | 480.1 | 102066.8 | 7.8 | 61.2 | 10 | W/B/L |
| Clinopodium chinense var. grandiflora (Maxim.) Kitag. | 264 |  |  |  | 490.4 | 104154.0 | 9.6 | 51.1 | 11 | W/B/L |
| Clinopodium chinense var. shibetchense (H.Lev.) Koidz. | 8 |  |  |  | 291.2 | 31806.4 | 50.7 | 5.7 | 2 | N/R/S |
| Clinopodium gracile (Benth.) Kuntze | 23 |  |  |  | 350.2 | 59232.0 | 27.9 | 12.5 | 6 | W/R/S |
| Clinopodium gracile var. multicaule (Maxim.) Ohwi | 91 |  |  |  | 435.2 | 88797.1 | 12.7 | 34.2 | 9 | W/B/L |
| Clinopodium micranthum (Regel) Hara | 78 |  |  |  | 409.8 | 73352.4 | 12.6 | 32.5 | 7 | W/B/L |
| Clintonia udensis Trautv. & C.A.Mey. | 2 |  |  |  | 80.4 | NA | 80.4 | 1.0 | 2 | N/R/S |
| Cnidium japonicum Miq. | 2 |  |  |  | 336.6 | NA | 336.6 | 1.0 | 1 | W/R/S |
| Cnidium monnieri (L.) Cusson | 21 |  |  |  | 355.8 | 44194.5 | 24.6 | 14.4 | 8 | W/B/S |
| Cnidium officinale Makino | 4 |  |  |  | 384.3 | 23206.9 | 135.0 | 2.8 | 2 | W/R/S |
| Cocculus trilobus (Thunb.) DC. | 457 |  |  |  | 487.9 | 106175.4 | 6.6 | 74.3 | 13 | W/B/L |
| Codonopsis lanceolata (Siebold & Zucc.) Trautv. | 123 |  |  |  | 440.3 | 87524.6 | 12.3 | 35.8 | 7 | W/B/L |
| Codonopsis pilosula (Franch.) Nannf. | 5 |  |  |  | 272.0 | 6898.1 | 58.9 | 4.6 | 3 | N/B/S |
| Codonopsis ussuriensis (Rupr. & Maxim.) Hemsl. | 7 |  |  |  | 261.0 | 5693.4 | 41.0 | 6.4 | 5 | N/B/L |
| Coix lacrymajobi var. mayuen (Rom.Caill.) Stapf | 3 |  |  |  | 347.0 | 12882.4 | 163.2 | 2.1 | 2 | W/R/S |
| Commelina communis L. | 529 |  |  |  | 491.4 | 105239.7 | 6.9 | 71.6 | 13 | W/B/L |
| Commelina communis var. angustifolia Nakai | 50 |  |  |  | 421.6 | 66854.2 | 14.8 | 28.5 | 8 | W/B/S |
| Commelina mina Y.N.Lee & Y.Oh | 2 |  | V |  | 18.5 | NA | 18.5 | 1.0 | 2 | N/R/S |
| Coniogramme intermedia Hieron. | 20 |  |  |  | 289.1 | 33147.5 | 22.7 | 12.7 | 5 | N/B/L |
| Coniogramme japonica (Thunb.) Diels | 9 |  |  |  | 326.4 | 11875.0 | 43.6 | 7.5 | 3 | W/R/S |
| Convallaria keiskei Miq. | 238 |  |  |  | 413.1 | 79239.7 | 8.4 | 49.0 | 9 | W/B/L |
| Conyza bonariensis (L.) Cronquist | 75 |  |  |  | 469.2 | 86145.0 | 17.2 | 27.2 | 9 | W/B/S |
| Conyza canadensis (L.) Cronquist | 364 |  |  |  | 452.2 | 100663.9 | 7.9 | 57.2 | 11 | W/B/L |
| Conyza sumatrensis E.Walker | 14 |  |  |  | 372.8 | 50824.8 | 39.2 | 9.5 | 5 | W/R/S |
| Corchoropsis psilocarpa Harms & Loes. | 32 |  |  |  | 394.4 | 57875.8 | 21.8 | 18.1 | 5 | W/R/S |
| Corchoropsis tomentosa (Thunb.) Makino | 250 |  |  |  | 435.6 | 91790.6 | 9.0 | 48.4 | 9 | W/B/L |
| Coreanomecon hylomeconoides Nakai | 21 |  | V |  | 192.4 | 5753.4 | 11.1 | 17.3 | 5 | N/B/L |
| Coreopsis drumondii Torr. & Gray | 9 |  |  |  | 294.8 | 31245.5 | 58.3 | 5.1 | 5 | N/B/S |
| Coreopsis lanceolata L. | 44 |  |  |  | 420.7 | 68927.6 | 20.2 | 20.8 | 6 | W/R/S |
| Coreopsis tinctoria Nutt. | 13 |  |  |  | 286.4 | 38635.8 | 35.9 | 8.0 | 4 | N/B/L |
| Cornopteris crenulatoserrulata (Makino) Nakai | 13 |  |  |  | 318.7 | 43540.0 | 35.9 | 8.9 | 3 | W/R/S |
| Cornus controversa Hemsl. ex Prain | 304 |  |  |  | 472.7 | 84945.5 | 7.7 | 61.3 | 8 | W/B/L |
| Cornus kousa F.Buerger ex Miquel | 116 |  |  |  | 451.8 | 76001.7 | 10.8 | 42.0 | 9 | W/B/L |
| Cornus macrophylla Wall. | 35 |  |  |  | 406.9 | 74189.3 | 23.0 | 17.7 | 7 | W/B/S |
| Cornus officinalis Siebold & Zucc. | 56 |  |  |  | 380.2 | 77009.6 | 17.9 | 21.3 | 7 | W/B/S |
| Cornus walteri F.T.Wangerin | 61 |  |  |  | 404.6 | 77388.4 | 17.5 | 23.1 | 7 | W/B/S |
| Coronopus didymus (L.) Sm. | 2 |  |  |  | 196.3 | NA | 196.3 | 1.0 | 2 | N/R/S |
| Corydalis ambigua Cham. & Schleht. | 74 |  |  |  | 388.0 | 79214.9 | 15.6 | 24.8 | 8 | W/B/S |
| Corydalis grandicalyx B.U.Oh & Y.S.Kim | 7 |  | V |  | 124.2 | 3296.3 | 25.3 | 4.9 | 2 | N/R/S |
| Corydalis heterocarpa Siebold & Zucc. | 27 |  |  |  | 360.1 | 28864.3 | 12.5 | 28.8 | 6 | W/R/L |
| Corydalis incisa (Thunb.) Pers. | 41 |  |  |  | 375.0 | 33348.0 | 13.3 | 28.2 | 7 | W/B/S |
| Corydalis maculata B.U.Oh & Y.S.Kim | 20 |  | V |  | 298.4 | 33455.9 | 27.6 | 10.8 | 5 | N/B/L |
| Corydalis namdoensis B.U.oh & J.G.Kim | 5 |  |  |  | 177.3 | 1132.7 | 39.0 | 4.5 | 3 | N/B/S |
| Corydalis ochotensis Turcz. | 97 |  |  |  | 421.7 | 73843.0 | 12.1 | 34.7 | 9 | W/B/L |
| Corydalis pallida (Thunb.) Pers. | 7 |  |  |  | 420.9 | 23974.4 | 56.4 | 7.5 | 3 | W/R/S |
| Corydalis pauciovulata Ohwi | 95 |  |  |  | 357.3 | 57232.0 | 10.9 | 32.7 | 7 | W/B/L |
| Corydalis remota Fisch. ex Maxim. | 265 |  |  |  | 442.3 | 92276.7 | 9.0 | 49.3 | 7 | W/B/L |
| Corydalis speciosa Maxim. | 407 |  |  |  | 462.4 | 93632.4 | 7.2 | 63.9 | 12 | W/B/L |
| Corydalis ternata Nakai | 19 |  |  |  | 325.8 | 24578.3 | 15.8 | 20.6 | 5 | W/R/S |
| Corydalis turtschaninovii Besser | 15 |  |  |  | 294.5 | 23345.7 | 26.8 | 11.0 | 6 | N/B/L |
| Corydalis turtschaninovii for. fumariaefolia (Maxim.) T.B.Lee | 28 |  |  |  | 358.3 | 56050.3 | 25.0 | 14.3 | 5 | W/R/S |
| Corydalis turtschaninovii for. linearis (Regel) Nakai | 63 |  |  |  | 426.6 | 89604.9 | 17.3 | 24.7 | 10 | W/B/S |
| Corydalis turtschaninovii for. pectinata (Kom.) Y.H.Chou | 32 |  |  |  | 361.2 | 61616.5 | 22.9 | 15.8 | 7 | W/B/S |
| Corylopsis glabrescens var. gotoana Franch. & Sav. (Makino) T. Yamanaka | 27 |  |  |  | 333.7 | 10058.3 | 14.2 | 23.5 | 5 | W/R/S |
| Corylus heterophylla var. heterophylla Fisch. ex (Trautv.) | 385 |  |  |  | 457.6 | 96077.0 | 6.8 | 67.2 | 11 | W/B/L |
| Corylus sieboldiana var. mandshurica (Maxim. & Rupr.) C.K.Schneid. | 77 |  |  |  | 423.0 | 77395.8 | 16.7 | 25.4 | 7 | W/B/S |
| Corylus sieboldiana var. sieboldiana Blume | 146 |  |  |  | 451.1 | 83850.6 | 9.3 | 48.5 | 8 | W/B/L |
| Cosmos bipinnatus Cav. | 66 |  |  |  | 423.0 | 88588.6 | 17.5 | 24.1 | 6 | W/R/S |
| Cosmos sulphureus Cav. | 7 |  |  |  | 274.2 | 12685.1 | 48.4 | 5.7 | 3 | N/B/S |
| Cotinus coggygria Scop. | 3 |  |  |  | 87.8 | 1291.0 | 48.3 | 1.8 | 3 | N/B/S |
| Crassocephalum crepidioides (Benth.) S.Moore | 82 |  |  |  | 366.9 | 69810.5 | 11.6 | 31.5 | 6 | W/R/L |
| Crataegus pinnatifida for. pinnatifida Bunge | 125 |  |  |  | 439.0 | 79532.5 | 12.4 | 35.4 | 11 | W/B/L |
| Crataegus scabrida Sarg. | 5 |  |  |  | 231.1 | 6177.9 | 51.8 | 4.5 | 3 | N/B/S |
| Cremastra appendiculata (D.Don) Makino | 4 |  |  | NT | 180.2 | 2015.7 | 52.0 | 3.5 | 2 | N/R/S |
| Crepidiastrum chelidoniifolium (Makino) Pak & Kawano | 87 |  |  |  | 382.2 | 57497.5 | 11.5 | 33.3 | 5 | W/R/L |
| Crepidiastrum denticulatum (Houtt.) Pak & Kawano | 468 |  |  |  | 490.5 | 101873.3 | 7.2 | 68.5 | 10 | W/B/L |
| Crepidiastrum koidzumianum (Kitam.) Pak & Kawano | 7 |  | V |  | 336.7 | 13183.6 | 18.1 | 18.6 | 3 | W/R/S |
| Crepidiastrum sonchifolium (Bunge) Pak & Kawano | 343 |  |  |  | 488.9 | 98117.8 | 8.4 | 58.5 | 10 | W/B/L |
| Crotalaria sessiliflora L. | 19 |  |  |  | 313.0 | 44844.0 | 29.3 | 10.7 | 5 | N/B/L |
| Crypsinus hastatus (Thunb.) Copel. | 18 |  |  |  | 387.5 | 62466.5 | 26.1 | 14.9 | 7 | W/B/S |
| Cryptomeria japonica (L.f.) D.Don | 64 |  |  |  | 342.7 | 48114.4 | 10.6 | 32.3 | 7 | W/B/L |
| Cryptotaenia japonica Hassk. | 41 |  |  |  | 445.6 | 74273.4 | 21.1 | 21.2 | 6 | W/R/S |
| Cucubalus baccifer var. japonicus Miq. | 44 |  |  |  | 359.2 | 56394.9 | 20.1 | 17.8 | 9 | W/B/S |
| Cucumis melo var. makuwa Makino | 2 |  |  |  | 242.2 | NA | 242.2 | 1.0 | 1 | N/R/S |
| Cucurbita moschata Duchesne | 4 |  |  |  | 246.9 | 1963.7 | 10.4 | 23.7 | 2 | N/R/L |
| Cudrania tricuspidata (Carr.) Bureau ex Lavallee | 80 |  |  |  | 393.2 | 65125.1 | 11.7 | 33.6 | 8 | W/B/L |
| Cuscuta australis R.Br. | 92 |  |  |  | 450.6 | 84391.2 | 16.3 | 27.6 | 9 | W/B/S |
| Cuscuta japonica Choisy | 141 |  |  |  | 416.2 | 81478.3 | 10.5 | 39.7 | 9 | W/B/L |
| Cuscuta pentagona Engelm. | 51 |  |  |  | 450.0 | 77900.0 | 17.7 | 25.4 | 8 | W/B/S |
| Cyclosorus acuminatus (Houtt.) Nakai ex H.Ito | 5 |  |  |  | 220.9 | 13917.8 | 47.1 | 4.7 | 4 | N/B/S |
| Cymbidium goeringii (Rchb.f.) Rchb.f. | 70 |  |  | LC | 318.4 | 48843.8 | 13.2 | 24.1 | 6 | W/R/S |
| Cymbopogon tortilis var. goeringii (Steud.) Hand.-Mazz. | 178 |  |  |  | 477.7 | 101002.3 | 12.0 | 39.8 | 9 | W/B/L |
| Cymopterus melanotilingia (H.Boissieu) C.Y.Yoon | 78 |  |  |  | 423.9 | 65099.0 | 12.4 | 34.1 | 6 | W/R/L |
| Cynanchum ascyrifolium (Franch. & Sav.) Matsum. | 85 |  |  |  | 413.7 | 78687.2 | 15.0 | 27.6 | 7 | W/B/S |
| Cynanchum atratum Bunge | 14 |  |  |  | 312.1 | 43190.2 | 40.0 | 7.8 | 6 | N/B/L |
| Cynanchum inamoenum (Maxim.) Loes. | 13 |  |  | NT | 297.9 | 11528.9 | 27.5 | 10.9 | 3 | N/B/L |
| Cynanchum nipponicum Matsum. | 5 |  |  |  | 280.9 | 13737.0 | 71.5 | 3.9 | 2 | N/R/S |
| Cynanchum paniculatum (Bunge) Kitag. | 76 |  |  |  | 419.4 | 84163.5 | 14.0 | 29.9 | 5 | W/R/L |
| Cynanchum wilfordii (Maxim.) Hemsl. | 42 |  |  |  | 387.4 | 70957.7 | 22.3 | 17.3 | 4 | W/R/S |
| Cynodon dactylon (L.) Pers. | 3 |  |  |  | 313.1 | 4735.9 | 139.0 | 2.3 | 3 | N/B/S |
| Cyperus amuricus Maxim. | 262 |  |  |  | 447.2 | 98046.5 | 10.1 | 44.5 | 10 | W/B/L |
| Cyperus cyperoides (L.) Kuntze | 2 |  |  |  | 31.9 | NA | 31.9 | 1.0 | 1 | N/R/S |
| Cyperus difformis L. | 51 |  |  |  | 406.4 | 74534.3 | 21.4 | 19.0 | 6 | W/R/S |
| Cyperus exaltatus var. iwasakii T.Koyama | 6 |  |  |  | 375.9 | 37568.3 | 53.7 | 7.0 | 4 | W/R/S |
| Cyperus globosus All. | 12 |  |  |  | 475.9 | 59277.2 | 32.7 | 14.6 | 5 | W/R/S |
| Cyperus glomeratus L. | 7 |  |  |  | 211.4 | 16850.7 | 36.2 | 5.8 | 3 | N/B/S |
| Cyperus hakonensis Franch. & Sav. | 23 |  |  |  | 331.3 | 46245.7 | 24.9 | 13.3 | 5 | W/R/S |
| Cyperus haspan L. | 2 |  |  |  | 7.6 | NA | 7.6 | 1.0 | 2 | N/R/S |
| Cyperus iria L. | 88 |  |  |  | 451.5 | 87518.1 | 16.2 | 27.9 | 8 | W/B/S |
| Cyperus microiria Steud. | 140 |  |  |  | 463.1 | 92473.8 | 13.1 | 35.5 | 9 | W/B/L |
| Cyperus nipponicus Franch. & Sav. | 15 |  |  |  | 367.0 | 31418.6 | 36.3 | 10.1 | 6 | W/R/S |
| Cyperus orthostachyus Franch. & Sav. | 74 |  |  |  | 432.9 | 74966.3 | 17.3 | 25.0 | 7 | W/B/S |
| Cyperus rotundus L. | 2 |  |  |  | 199.6 | NA | 199.6 | 1.0 | 2 | N/R/S |
| Cyperus sanguinolentus Vahl | 23 |  |  |  | 476.1 | 71851.3 | 26.1 | 18.3 | 7 | W/B/S |
| Cyperus serotinus Rottb. | 3 |  |  |  | 351.9 | 19943.2 | 195.2 | 1.8 | 3 | W/R/S |
| Cyperus tenuispica Steud. | 3 |  |  |  | 220.0 | 12897.6 | 157.1 | 1.4 | 2 | N/R/S |
| Cypripedium macranthos Sw. | 5 | V |  | EN | 104.9 | 4305.1 | 39.4 | 2.7 | 1 | N/R/S |
| Cyrtomium caryotideum var. coreanum Nakai | 2 |  |  |  | 390.0 | NA | 390.0 | 1.0 | 1 | W/R/S |
| Cyrtomium falcatum (L.f.) C.Presl | 13 |  |  |  | 354.8 | 34216.2 | 19.6 | 18.1 | 3 | W/R/S |
| Cyrtomium fortunei J.Sm. | 61 |  |  |  | 413.5 | 66780.8 | 14.2 | 29.1 | 6 | W/R/L |
| Dactylis glomerata L. | 211 |  |  |  | 455.3 | 97232.4 | 10.3 | 44.4 | 10 | W/B/L |
| Daphne genkwa Siebold & Zucc. | 3 |  |  |  | 0.6 | 0.0 | 0.3 | 2.0 | 2 | N/R/S |
| Daphne pseudomezereum var. koreana A. Gray (Nakai) Hamaya | 2 |  |  | VU | 17.4 | NA | 17.4 | 1.0 | 1 | N/R/S |
| Daphniphyllum macropodum Miq. | 4 |  |  |  | 116.2 | 3044.2 | 38.8 | 3.0 | 2 | N/R/S |
| Datura stramonium var. chalybea Koch | 3 |  |  |  | 340.2 | 23364.1 | 204.6 | 1.7 | 3 | W/R/S |
| Datura stramonium var. stramonium L. | 4 |  |  |  | 366.5 | 1843.5 | 96.5 | 3.8 | 3 | W/R/S |
| Davallia mariesii T.Moore ex Baker | 107 |  |  |  | 453.5 | 81056.7 | 12.1 | 37.4 | 7 | W/B/L |
| Delphinium maackianum for. album (Nakai) W.T.Lee | 2 |  |  |  | 0.9 | NA | 0.9 | 1.0 | 1 | N/R/S |
| Delphinium maackianum Regel | 13 |  |  |  | 241.8 | 14365.6 | 24.4 | 9.9 | 4 | N/B/L |
| Dendranthema boreale (Makino) Ling ex Kitam. | 284 |  |  |  | 455.4 | 99017.8 | 9.8 | 46.6 | 10 | W/B/L |
| Dendranthema indicum (L.) DesMoul. | 25 |  |  |  | 387.0 | 66139.8 | 24.6 | 15.7 | 6 | W/R/S |
| Dendranthema sichotense Tzvelev | 8 |  |  |  | 194.2 | 6789.1 | 24.9 | 7.8 | 2 | N/R/L |
| Dendranthema zawadskii var. latilobum (Maxim.) Kitam. | 312 |  |  |  | 477.8 | 93453.0 | 8.0 | 59.7 | 8 | W/B/L |
| Dendranthema zawadskii var. tenuisectum Kitag. | 25 |  |  |  | 200.5 | 13424.6 | 10.1 | 19.9 | 8 | N/B/L |
| Dendranthema zawadskii var. zawadskii (Herb.) Tzvelev | 62 |  |  |  | 428.7 | 78849.5 | 13.6 | 31.6 | 7 | W/B/L |
| Dendropanax trifidus (Thunb.) Makino ex H. Hara | 7 |  |  |  | 34.1 | 325.4 | 4.3 | 8.0 | 3 | N/B/L |
| Dennstaedtia hirsuta (Sw.) Mett. ex Miq. | 123 |  |  |  | 463.8 | 87012.6 | 12.4 | 37.3 | 9 | W/B/L |
| Dennstaedtia wilfordii (T.Moore) H.Christ | 208 |  |  |  | 442.5 | 72336.2 | 8.3 | 53.1 | 7 | W/B/L |
| Deparia conilii (Franch. & Sav.) M.Kato | 75 |  |  |  | 427.3 | 82081.0 | 16.1 | 26.6 | 8 | W/B/S |
| Deparia coreana (H.Christ) M.Kato | 19 |  |  |  | 337.0 | 23161.1 | 20.3 | 16.6 | 4 | W/R/S |
| Deparia dimorphophylla (Koidz.) M.Kato | 4 |  |  |  | 240.1 | 10492.2 | 100.0 | 2.4 | 2 | N/R/S |
| Deparia japonica (Thunb.) M.Kato | 77 |  |  |  | 476.9 | 67872.6 | 12.4 | 38.5 | 7 | W/B/L |
| Deparia oldhamii (Hook. & Baker) Y.C.Oh & C.S.Lee | 2 |  |  |  | 0.4 | NA | 0.4 | 1.0 | 1 | N/R/S |
| Deparia pterorachis (H.Christ) M.Kato | 5 |  |  |  | 288.8 | 26850.2 | 97.7 | 3.0 | 3 | N/B/S |
| Deparia pycnosora (H.Christ) M.Kato | 40 |  |  |  | 450.9 | 55349.1 | 21.2 | 21.2 | 6 | W/R/S |
| Descurainia pinnata Britton | 4 |  |  |  | 104.5 | 1109.0 | 13.7 | 7.7 | 2 | N/R/L |
| Descurainia sophia (L.) Webb ex Prantl | 20 |  |  |  | 341.3 | 41287.4 | 17.8 | 19.2 | 5 | W/R/S |
| Desmodium oldhami Oliv. | 58 |  |  |  | 402.9 | 71019.1 | 19.8 | 20.4 | 7 | W/B/S |
| Desmodium podocarpum DC. | 51 |  |  |  | 365.3 | 46940.5 | 15.1 | 24.1 | 6 | W/R/S |
| Desmodium podocarpum var. mandshuricum Maxim. | 15 |  |  |  | 385.9 | 51457.9 | 41.9 | 9.2 | 4 | W/R/S |
| Desmodium podocarpum var. oxyphyllum (DC.) H.Ohashi | 190 |  |  |  | 463.6 | 92985.0 | 9.9 | 46.9 | 9 | W/B/L |
| Deutzia crenata for. plena Schneid | 2 |  |  |  | 240.9 | NA | 240.9 | 1.0 | 2 | N/R/S |
| Deutzia glabrata Kom. | 119 |  |  |  | 357.1 | 64211.1 | 9.8 | 36.3 | 6 | W/R/L |
| Deutzia grandiflora var. baroniana Diels | 56 |  |  |  | 438.8 | 70107.0 | 14.0 | 31.4 | 5 | W/R/L |
| Deutzia paniculata Nakai | 5 |  | V | VU | 349.3 | 1643.4 | 73.6 | 4.7 | 3 | W/R/S |
| Deutzia parviflora Bunge | 73 |  |  |  | 408.8 | 81608.2 | 16.3 | 25.2 | 6 | W/R/S |
| Deutzia uniflora Shirai | 213 |  |  |  | 419.6 | 77211.6 | 7.7 | 54.7 | 8 | W/B/L |
| Dianthus chinensis var. chinensis L. | 181 |  |  |  | 449.7 | 92283.1 | 11.0 | 40.7 | 10 | W/B/L |
| Dianthus longicalyx Miq. | 59 |  |  |  | 430.5 | 82048.6 | 17.6 | 24.4 | 6 | W/R/S |
| Diarrhena fauriei (Hack.) Ohwi | 17 |  |  |  | 248.9 | 25291.1 | 23.4 | 10.6 | 5 | N/B/L |
| Diarrhena japonica (Franch. & Sav.) Franch. & Sav. | 57 |  |  |  | 334.2 | 57572.7 | 14.5 | 23.0 | 7 | W/B/S |
| Diarrhena mandshurica Maxim. | 39 |  |  |  | 286.2 | 42622.0 | 14.5 | 19.7 | 4 | N/B/L |
| Dicentra spectabilis (L.) Lem. | 65 |  |  |  | 351.3 | 57500.9 | 13.2 | 26.6 | 7 | W/B/S |
| Dicranopteris linearis (Burm. f.) Underw. | 4 |  |  |  | 171.0 | 1810.3 | 50.3 | 3.4 | 3 | N/B/S |
| Dictamnus dasycarpus Turcz. | 181 |  |  |  | 481.8 | 98740.0 | 10.6 | 45.5 | 9 | W/B/L |
| Digitaria ciliaris (Retz.) Koel. | 278 |  |  |  | 454.3 | 97585.0 | 10.0 | 45.4 | 12 | W/B/L |
| Digitaria radicosa (Presl) Miq. | 28 |  |  |  | 397.6 | 56225.6 | 24.8 | 16.0 | 8 | W/B/S |
| Digitaria violascens Link | 43 |  |  |  | 422.7 | 72403.7 | 25.1 | 16.8 | 8 | W/B/S |
| Dimeria ornithopoda Trin. | 5 |  |  |  | 161.4 | 5431.4 | 54.1 | 3.0 | 4 | N/B/S |
| Diodia teres var. teres Walter | 14 |  |  |  | 328.9 | 34355.4 | 24.7 | 13.3 | 4 | W/R/S |
| Dioscorea bulbifera L. | 11 |  |  |  | 354.3 | 46039.1 | 54.8 | 6.5 | 6 | W/R/S |
| Dioscorea japonica Thunb. | 142 |  |  |  | 470.4 | 91321.5 | 9.9 | 47.6 | 8 | W/B/L |
| Dioscorea nipponica Makino | 118 |  |  |  | 449.6 | 86468.2 | 11.6 | 38.8 | 7 | W/B/L |
| Dioscorea oppostifolia L. | 207 |  |  |  | 450.9 | 94351.8 | 10.4 | 43.2 | 8 | W/B/L |
| Dioscorea quinqueloba Thunb. | 177 |  |  |  | 475.4 | 93352.4 | 9.8 | 48.3 | 10 | W/B/L |
| Dioscorea septemloba Thunb. | 28 |  |  |  | 417.2 | 60835.7 | 19.1 | 21.8 | 6 | W/R/S |
| Dioscorea tenuipes Franch. & Sav. | 59 |  |  |  | 421.8 | 78224.2 | 14.6 | 28.9 | 6 | W/R/L |
| Dioscorea tokoro Makino | 87 |  |  |  | 455.5 | 90016.3 | 14.7 | 31.1 | 6 | W/R/L |
| Diospyros kaki Thunb. | 67 |  |  |  | 442.6 | 69909.7 | 17.4 | 25.5 | 8 | W/B/S |
| Diospyros lotus L. | 143 |  |  |  | 449.7 | 81923.6 | 11.5 | 39.2 | 12 | W/B/L |
| Diplazium chinense (Baker) C.Chr. | 3 |  |  |  | 315.2 | 22980.2 | 203.3 | 1.6 | 3 | W/R/S |
| Diplazium mesosorum (Mak.) Makino | 5 |  |  |  | 178.8 | 9209.4 | 35.4 | 5.1 | 5 | N/B/S |
| Diplazium squamigerum (Mett.) Hope | 4 |  |  |  | 207.2 | 1615.8 | 59.7 | 3.5 | 2 | N/R/S |
| Dipsacus japonicus Miq. | 13 |  |  | LC | 174.8 | 7262.2 | 10.3 | 17.1 | 4 | N/B/L |
| Disporum smilacinum A.Gray | 521 |  |  |  | 495.2 | 104093.5 | 5.6 | 89.1 | 10 | W/B/L |
| Disporum uniflorum Baker | 147 |  |  |  | 420.6 | 81621.6 | 10.4 | 40.5 | 8 | W/B/L |
| Disporum viridescens (Maxim.) Nakai | 165 |  |  |  | 439.6 | 91634.6 | 10.6 | 41.4 | 9 | W/B/L |
| Dontostemon dentatus (Bunge) Ledeb. | 16 |  |  |  | 372.6 | 42746.1 | 26.0 | 14.3 | 6 | W/R/S |
| Draba nemorosa for. leiocarpa (Lindb.) Kitag. | 2 |  |  |  | 18.0 | NA | 18.0 | 1.0 | 2 | N/R/S |
| Draba nemorosa for. nemorosa L. | 343 |  |  |  | 449.9 | 96067.6 | 8.2 | 55.1 | 13 | W/B/L |
| Dracocephalum argunense Fisch. ex Link | 5 |  |  |  | 278.6 | 2818.4 | 34.3 | 8.1 | 3 | N/B/L |
| Drosera peltata var. nipponica (Masam.) Ohwi | 2 | V |  | VU | 1.1 | NA | 1.1 | 1.0 | 2 | N/R/S |
| Drosera rotundifolia L. | 4 |  |  | LC | 454.3 | 44439.5 | 128.7 | 3.5 | 2 | W/R/S |
| Dryopteris bissetiana (Baker) C.Chr. | 71 |  |  |  | 435.0 | 70695.0 | 13.1 | 33.2 | 8 | W/B/L |
| Dryopteris chinensis (Baker) Koidz. | 303 |  |  |  | 485.0 | 96509.1 | 7.9 | 61.7 | 9 | W/B/L |
| Dryopteris crassirhizoma Nakai | 129 |  |  |  | 357.3 | 69813.0 | 10.0 | 35.9 | 7 | W/B/L |
| Dryopteris erythrosora (D.C.Eaton) Kuntze | 36 |  |  |  | 277.7 | 29939.6 | 10.8 | 25.8 | 4 | N/B/L |
| Dryopteris expansa (C.Presl) Fraser-Jenk. & Jermy | 7 |  |  |  | 331.8 | 20340.6 | 57.0 | 5.8 | 3 | W/R/S |
| Dryopteris fragrans (L.) Schott | 4 |  |  |  | 316.0 | 11249.9 | 93.6 | 3.4 | 3 | W/R/S |
| Dryopteris gymnophylla (Baker) C.Chr. | 41 |  |  |  | 327.6 | 46850.4 | 15.8 | 20.7 | 6 | W/R/S |
| Dryopteris lacera (Thunb.) Kuntze | 161 |  |  |  | 442.1 | 82744.1 | 8.5 | 51.9 | 7 | W/B/L |
| Dryopteris laeta (Kom.) C.Chr. | 5 |  |  |  | 338.1 | 27876.5 | 122.9 | 2.8 | 2 | W/R/S |
| Dryopteris monticola (Makino) C.Chr. | 46 |  |  |  | 360.1 | 58894.5 | 14.6 | 24.6 | 4 | W/R/S |
| Dryopteris nipponensis Koidz. | 2 |  |  |  | 102.1 | NA | 102.1 | 1.0 | 2 | N/R/S |
| Dryopteris pacifica (Nakai) Tagawa | 22 |  |  |  | 298.9 | 38068.1 | 22.9 | 13.1 | 5 | N/B/L |
| Dryopteris polylepis (Franch. & Sav.) C.Chr. | 3 |  |  |  | 128.0 | 1556.5 | 67.2 | 1.9 | 3 | N/B/S |
| Dryopteris sacrosanta Koidz. | 80 |  |  |  | 376.6 | 60148.7 | 12.2 | 30.8 | 6 | W/R/L |
| Dryopteris saxifraga H.Ito | 93 |  |  |  | 411.2 | 79528.5 | 12.5 | 32.8 | 6 | W/R/L |
| Dryopteris uniformis (Makino) Makino | 32 |  |  |  | 400.1 | 66526.4 | 26.6 | 15.0 | 6 | W/R/S |
| Dryopteris varia (L.) Kuntze | 178 |  |  |  | 440.2 | 87086.5 | 8.5 | 51.5 | 11 | W/B/L |
| Duchesnea chrysantha (Zoll. & Mor.) Miq. | 420 |  |  |  | 471.6 | 100831.9 | 7.5 | 63.0 | 9 | W/B/L |
| Dunbaria villosa (Thunb.) Makino | 43 |  |  |  | 440.0 | 64652.4 | 19.6 | 22.5 | 6 | W/R/S |
| Echinochloa crusgalli var. crusgalli (L.) P.Beauv. | 282 |  |  |  | 461.4 | 99756.6 | 9.5 | 48.8 | 10 | W/B/L |
| Echinochloa crusgalli var. frumentacea (Link) W.Wight | 27 |  |  |  | 362.6 | 56350.0 | 24.7 | 14.7 | 5 | W/R/S |
| Echinochloa crusgalli var. oryzicola (Vasinger) Ohwi | 35 |  |  |  | 437.3 | 71701.4 | 26.2 | 16.7 | 7 | W/B/S |
| Echinochloa crusgalli var. praticola Ohwi | 2 |  |  |  | 202.2 | NA | 202.2 | 1.0 | 2 | N/R/S |
| Echinops latifolius Tausch | 3 |  |  |  | 64.2 | 242.3 | 27.5 | 2.3 | 1 | N/R/S |
| Echinops setifer Iljin | 29 |  |  |  | 381.7 | 62948.2 | 24.1 | 15.8 | 3 | W/R/S |
| Echinosophora koreensis (Nakai) Nakai | 3 | V | V |  | 235.6 | 3807.1 | 100.5 | 2.3 | 2 | N/R/S |
| Eclipta prostrata (L.) L. | 125 |  |  |  | 419.3 | 87948.9 | 12.1 | 34.6 | 9 | W/B/L |
| Elaeagnus glabra Thunb. | 5 |  |  |  | 321.3 | 17638.8 | 54.2 | 5.9 | 3 | W/R/S |
| Elaeagnus macrophylla Thunb. | 21 |  |  |  | 324.6 | 53682.3 | 32.3 | 10.1 | 5 | W/R/S |
| Elaeagnus multiflora Thunb. | 10 |  |  |  | 326.0 | 33978.4 | 47.3 | 6.9 | 4 | W/R/S |
| Elaeagnus umbellata Thunb. | 298 |  |  |  | 435.4 | 94444.4 | 8.3 | 52.7 | 11 | W/B/L |
| Elatine triandra Schkuhr | 2 |  |  |  | 240.6 | NA | 240.6 | 1.0 | 1 | N/R/S |
| Eleocharis acicularis for. longiseta (Svenson) T.Koyama | 14 |  |  |  | 349.7 | 40676.4 | 38.7 | 9.0 | 7 | W/B/S |
| Eleocharis attenuata for. laeviseta (Nakai) Hara | 5 |  |  |  | 255.0 | 13564.0 | 44.7 | 5.7 | 4 | N/B/S |
| Eleocharis congesta var. japonica (Miq.) T.Koyama | 20 |  |  |  | 372.8 | 49177.5 | 31.6 | 11.8 | 5 | W/R/S |
| Eleocharis kuroguwai Ohwi | 2 |  |  |  | 98.8 | NA | 98.8 | 1.0 | 2 | N/R/S |
| Eleocharis mamillata var. cyclocarpa Kitag. | 6 |  |  |  | 282.0 | 29659.8 | 56.8 | 5.0 | 4 | N/B/S |
| Eleocharis tetraquetra Nees ex Wight | 9 |  |  |  | 430.8 | 47183.5 | 68.7 | 6.3 | 2 | W/R/S |
| Eleusine indica (L.) Gaertn. | 145 |  |  |  | 457.4 | 97399.0 | 13.3 | 34.5 | 10 | W/B/L |
| Eleutherococcus divaricatus var. chiisanensis (Nakai) C.H.Kim & B.Y.Sun | 14 |  | V | LC | 246.3 | 21990.6 | 29.0 | 8.5 | 4 | N/B/L |
| Eleutherococcus senticosus (Rupr. & Maxim.) Maxim. | 5 | V |  | VU | 191.6 | 3280.3 | 49.3 | 3.9 | 3 | N/B/S |
| Eleutherococcus sessiliflorus (Rupr. & Maxim.) S.Y.Hu | 82 |  |  |  | 413.3 | 72381.6 | 13.7 | 30.2 | 7 | W/B/L |
| Eleutherococcus sieboldianus (Makino) Koidz. | 2 |  |  |  | 75.0 | NA | 75.0 | 1.0 | 2 | N/R/S |
| Elsholtzia ciliata (Thunb.) Hyl. | 201 |  |  |  | 423.6 | 93670.0 | 10.8 | 39.3 | 9 | W/B/L |
| Elsholtzia splendens Nakai | 251 |  |  |  | 457.7 | 89172.7 | 9.4 | 48.7 | 9 | W/B/L |
| Elymus mollis Trin. | 3 |  |  |  | 459.4 | 26478.5 | 225.3 | 2.0 | 3 | W/R/S |
| Elymus sibiricus L. | 12 |  |  |  | 329.0 | 19405.4 | 27.1 | 12.1 | 2 | W/R/S |
| Epilobium cephalostigma Hausskn. | 14 |  |  |  | 288.7 | 15866.2 | 25.5 | 11.3 | 4 | N/B/L |
| Epilobium pyrricholophum Franch. & Sav. | 32 |  |  |  | 433.1 | 52748.7 | 20.0 | 21.6 | 7 | W/B/S |
| Epimedium koreanum Nakai | 15 |  |  | LC | 121.9 | 5352.9 | 7.7 | 15.8 | 4 | N/B/L |
| Epipactis papillosa Franch. & Sav. | 3 |  |  | NT | 44.9 | 595.8 | 34.7 | 1.3 | 2 | N/R/S |
| Epipactis thunbergii A.Gray | 13 |  |  |  | 379.5 | 59895.4 | 39.2 | 9.7 | 4 | W/R/S |
| Equisetum arvense L. | 441 |  |  |  | 437.8 | 98435.7 | 7.1 | 61.6 | 13 | W/B/L |
| Equisetum hyemale L. | 27 |  |  |  | 286.1 | 37971.0 | 21.3 | 13.4 | 4 | N/B/L |
| Equisetum palustre L. | 7 |  |  |  | 245.7 | 12793.5 | 46.2 | 5.3 | 4 | N/B/S |
| Equisetum pratense Ehrh. | 2 |  |  |  | 0.2 | NA | 0.2 | 1.0 | 2 | N/R/S |
| Equisetum ramosissimum Desf. | 7 |  |  |  | 114.1 | 3418.2 | 24.2 | 4.7 | 4 | N/B/S |
| Equisetum sylvaticum L. | 2 |  |  |  | 9.5 | NA | 9.5 | 1.0 | 2 | N/R/S |
| Eragrostis cilianensis (All.) Link ex Vignolo | 6 |  |  |  | 386.7 | 36733.4 | 45.5 | 8.5 | 3 | W/R/S |
| Eragrostis curvula Nees | 14 |  |  |  | 291.6 | 39041.5 | 32.4 | 9.0 | 6 | N/B/L |
| Eragrostis ferruginea (Thunb.) P.Beauv. | 233 |  |  |  | 489.1 | 100549.2 | 9.3 | 52.5 | 9 | W/B/L |
| Eragrostis japonica (Thunb.) Trin. | 6 |  |  |  | 156.4 | 10705.5 | 54.7 | 2.9 | 4 | N/B/S |
| Eragrostis multicaulis Steud. | 49 |  |  |  | 445.9 | 66708.8 | 20.9 | 21.4 | 7 | W/B/S |
| Eragrostis pilosa (L.) P.Beauv. | 23 |  |  |  | 344.7 | 49355.6 | 29.4 | 11.7 | 6 | W/R/S |
| Eranthis byunsanensis B.Y.Sun | 6 |  | V | LC | 253.7 | 22944.1 | 67.1 | 3.8 | 3 | N/B/S |
| Eranthis stellata Maxim. | 42 |  |  |  | 335.3 | 31454.1 | 19.3 | 17.4 | 4 | W/R/S |
| Erechtites hieracifolia Raf. | 193 |  |  |  | 443.8 | 93560.3 | 10.3 | 43.0 | 9 | W/B/L |
| Erigeron annuus (L.) Pers. | 503 |  |  |  | 468.9 | 102636.7 | 7.2 | 65.2 | 11 | W/B/L |
| Erigeron philadelphicus L. | 3 |  |  |  | 68.4 | 7.6 | 25.2 | 2.7 | 2 | N/R/S |
| Erigeron strigosus Muhl. | 12 |  |  |  | 233.1 | 27747.1 | 20.7 | 11.2 | 5 | N/B/L |
| Eriocaulon decemflorum Maxim. | 4 |  |  |  | 200.1 | 11883.0 | 77.6 | 2.6 | 3 | N/B/S |
| Eriocaulon miquelianum Koern. | 3 |  |  |  | 367.4 | 27287.5 | 221.7 | 1.7 | 1 | W/R/S |
| Eriocaulon robustius (Maxim.) Makino | 4 |  |  |  | 145.7 | 2594.1 | 20.8 | 7.0 | 3 | N/B/L |
| Eriocaulon sieboldianum Siebold & Zucc. | 2 |  |  |  | 106.8 | NA | 106.8 | 1.0 | 2 | N/R/S |
| Eriocaulon sikokianum Maxim. | 4 |  |  |  | 367.9 | 37324.4 | 184.5 | 2.0 | 3 | W/R/S |
| Eriochloa villosa (Thunb.) Kunth | 161 |  |  |  | 446.4 | 89082.3 | 11.4 | 39.0 | 11 | W/B/L |
| Erythronium japonicum (Balrer) Decne. | 90 |  |  |  | 428.8 | 79089.9 | 12.0 | 35.7 | 8 | W/B/L |
| Eucommia ulmoides Oliv. | 29 |  |  |  | 274.8 | 39811.0 | 20.3 | 13.5 | 6 | N/B/L |
| Eulalia quadrinervis (Hack.) Kuntze | 5 |  |  |  | 263.5 | 17650.6 | 83.3 | 3.2 | 4 | N/B/S |
| Eulalia speciosa (Debeaux) Kuntze | 7 |  |  |  | 314.9 | 24384.2 | 64.1 | 4.9 | 4 | N/B/S |
| Euonymus alatus (Thunb.) Siebold | 248 |  |  |  | 475.4 | 101966.3 | 9.3 | 51.1 | 11 | W/B/L |
| Euonymus alatus for. ciliatodentatus (Franch. & Sav.) Hiyama | 274 |  |  |  | 442.3 | 97232.4 | 8.4 | 52.6 | 9 | W/B/L |
| Euonymus alatus for. striatus (Thunb.) Makino | 15 |  |  |  | 151.5 | 5701.7 | 6.8 | 22.4 | 4 | N/B/L |
| Euonymus bungeanus Maxim. | 8 |  |  |  | 179.7 | 10401.3 | 18.0 | 10.0 | 6 | N/B/L |
| Euonymus fortunei var. radicans (Miq.) Rehder | 15 |  |  |  | 361.8 | 29940.4 | 35.8 | 10.1 | 4 | W/R/S |
| Euonymus hamiltonianus var. hamiltonianus Wall. | 74 |  |  |  | 433.6 | 70078.2 | 12.8 | 33.7 | 7 | W/B/L |
| Euonymus hamiltonianus var. maackii (Rupr.) Kom. | 14 |  |  |  | 380.0 | 50618.2 | 55.1 | 6.9 | 4 | W/R/S |
| Euonymus japonicus Thunb. | 61 |  |  |  | 455.9 | 86971.2 | 17.6 | 25.9 | 7 | W/B/S |
| Euonymus macropterus Rupr. | 52 |  |  |  | 388.2 | 64409.5 | 17.7 | 21.9 | 3 | W/R/S |
| Euonymus oxyphyllus Miq. | 174 |  |  |  | 468.5 | 91844.6 | 10.1 | 46.2 | 10 | W/B/L |
| Euonymus pauciflorus Maxim. | 34 |  |  |  | 423.9 | 74028.2 | 22.1 | 19.1 | 4 | W/R/S |
| Euonymus sachalinensis (F.Schmidt) Maxim. | 94 |  |  |  | 441.9 | 81017.7 | 12.4 | 35.7 | 6 | W/R/L |
| Euonymus trapococcus Nakai | 12 |  |  |  | 350.3 | 49821.0 | 58.1 | 6.0 | 3 | W/R/S |
| Eupatorium japonicum Thunb. ex Murray | 515 |  |  |  | 495.3 | 107067.7 | 6.9 | 71.4 | 10 | W/B/L |
| Eupatorium lindleyanum | 82 |  |  |  | 454.1 | 86813.9 | 16.5 | 27.5 | 7 | W/B/S |
| Eupatorium makinoi var. oppisitifolium (Koidz.) Kawahara & Yahara | 141 |  |  |  | 469.1 | 89495.1 | 9.6 | 49.1 | 7 | W/B/L |
| Eupatorium rugosum Houtt. | 16 |  |  |  | 348.6 | 14481.5 | 16.7 | 20.9 | 4 | W/R/S |
| Eupatorium tripartitum (Makino) Murata & H.Koyama | 16 |  |  |  | 382.6 | 38566.5 | 25.5 | 15.0 | 4 | W/R/S |
| Euphorbia ebracteolata Hayata | 6 |  |  |  | 122.7 | 4447.7 | 27.5 | 4.5 | 4 | N/B/S |
| Euphorbia esula L. | 8 |  |  |  | 303.6 | 23614.6 | 56.0 | 5.4 | 5 | N/B/S |
| Euphorbia helioscopia L. | 14 |  |  |  | 363.1 | 49815.0 | 40.2 | 9.0 | 4 | W/R/S |
| Euphorbia humifusa Willd. ex Schltdl. | 43 |  |  |  | 432.4 | 78535.2 | 21.7 | 20.0 | 6 | W/R/S |
| Euphorbia maculata L. | 54 |  |  |  | 299.1 | 42642.9 | 13.4 | 22.3 | 8 | N/B/L |
| Euphorbia pekinensis Rupr. | 53 |  |  |  | 401.9 | 76439.7 | 15.9 | 25.3 | 9 | W/B/S |
| Euphorbia sieboldiana Morren & Decne. | 127 |  |  |  | 427.0 | 80119.4 | 12.8 | 33.3 | 8 | W/B/L |
| Euphorbia supina Raf. | 47 |  |  |  | 408.6 | 71824.4 | 20.7 | 19.8 | 8 | W/B/S |
| Euphrasia maximowiczii Wettst. | 2 |  |  |  | 123.7 | NA | 123.7 | 1.0 | 2 | N/R/S |
| Eurya emarginata (Thunb.) Makino | 2 |  |  |  | 91.5 | NA | 91.5 | 1.0 | 2 | N/R/S |
| Eurya japonica Thunb. | 81 |  |  |  | 372.4 | 56104.6 | 8.3 | 44.8 | 9 | W/B/L |
| Euscaphis japonica (Thunb.) Kanitz | 61 |  |  |  | 309.0 | 35049.9 | 8.9 | 34.6 | 4 | N/B/L |
| Evodia daniellii Hemsl. | 23 |  |  |  | 365.4 | 61719.6 | 34.6 | 10.6 | 5 | W/R/S |
| Exochorda serratifolia var. serratifolia S.Moore | 39 |  |  | LC | 263.6 | 24070.1 | 9.8 | 27.0 | 6 | N/B/L |
| Fagopyrum esculentum Moench | 48 |  |  |  | 354.1 | 59391.5 | 20.9 | 17.0 | 7 | W/B/S |
| Fallopia ciliinervis (Nakai) Hammer | 13 |  |  |  | 363.2 | 26328.4 | 29.8 | 12.2 | 3 | W/R/S |
| Fallopia convolvulus (L.) A.Love | 13 |  |  |  | 391.9 | 52401.6 | 41.8 | 9.4 | 6 | W/R/S |
| Fallopia dentatoalata (F.Schmidt) Holub | 32 |  |  |  | 359.0 | 58294.6 | 22.6 | 15.9 | 6 | W/R/S |
| Fallopia dumetorum (L.) Holub | 157 |  |  |  | 415.4 | 93796.7 | 11.0 | 37.9 | 8 | W/B/L |
| Fallopia forbesii (Hance) K.Yonekura & Ohashi | 3 |  |  |  | 161.6 | 282.2 | 56.2 | 2.9 | 3 | N/B/S |
| Fallopia japonica (Houtt.) RonseDecr. | 29 |  |  |  | 480.0 | 75982.2 | 30.3 | 15.8 | 7 | W/B/S |
| Fallopia koreana B.U.Oh & J.G.Kim | 6 |  | V |  | 116.0 | 5678.7 | 23.6 | 4.9 | 3 | N/B/S |
| Fallopia multiflora var. multiflora (Thunb. ex Murray) Haraldson | 13 |  |  |  | 366.1 | 51314.2 | 36.3 | 10.1 | 5 | W/R/S |
| Farfugium japonicum (L.) Kitam. | 3 |  |  |  | 111.3 | 327.9 | 40.9 | 2.7 | 3 | N/B/S |
| Fatoua villosa (Thunb.) Nakai | 20 |  |  |  | 357.6 | 58130.1 | 30.9 | 11.6 | 7 | W/B/S |
| Fatsia japonica (Thunb.) Decne. & Planch. | 6 |  |  |  | 168.2 | 3632.2 | 31.9 | 5.3 | 3 | N/B/S |
| Festuca arundinacea Schreb. | 86 |  |  |  | 382.3 | 59333.0 | 12.8 | 29.8 | 7 | W/B/L |
| Festuca ovina var. ovina L. | 167 |  |  |  | 456.8 | 93341.0 | 11.1 | 41.0 | 7 | W/B/L |
| Festuca parvigluma Steud. | 47 |  |  |  | 382.0 | 65402.5 | 18.2 | 21.0 | 6 | W/R/S |
| Festuca pratensis Huds. | 3 |  |  |  | 363.2 | 9467.7 | 157.7 | 2.3 | 2 | W/R/S |
| Festuca rubra L. | 11 |  |  |  | 230.6 | 21674.4 | 39.9 | 5.8 | 4 | N/B/S |
| Festuca subulata var. japonica Hack. | 17 |  |  |  | 396.0 | 50290.8 | 41.8 | 9.5 | 5 | W/R/S |
| Ficus carica L. | 4 |  |  |  | 12.6 | 56.6 | 6.9 | 1.8 | 3 | N/B/S |
| Ficus erecta Thunb. | 7 |  |  |  | 176.0 | 4306.6 | 26.6 | 6.6 | 4 | N/B/L |
| Filifolium sibiricum (L.) Kitam. | 8 |  |  |  | 335.4 | 36885.9 | 55.0 | 6.1 | 5 | W/R/S |
| Filipendula glaberrima (Nakai) Nakai | 70 |  |  |  | 331.1 | 54735.5 | 12.2 | 27.2 | 4 | W/R/S |
| Fimbristylis autumnalis (L.) Roem. & Schult. | 11 |  |  |  | 315.1 | 37015.4 | 42.7 | 7.4 | 4 | W/R/S |
| Fimbristylis complanata for. exalata T.Koyama | 6 |  |  |  | 243.2 | 18102.9 | 58.1 | 4.2 | 2 | N/R/S |
| Fimbristylis dichotoma for. dichotoma (L.) Vahl | 41 |  |  |  | 431.7 | 79859.4 | 22.3 | 19.4 | 9 | W/B/S |
| Fimbristylis globosa var. austrojaponica Ohwi | 5 |  |  |  | 97.7 | 1914.5 | 22.7 | 4.3 | 4 | N/B/S |
| Fimbristylis longispica Steud. | 3 |  |  |  | 147.3 | 876.4 | 56.5 | 2.6 | 2 | N/R/S |
| Fimbristylis miliacea (L.) Vahl | 39 |  |  |  | 424.4 | 61495.7 | 20.0 | 21.2 | 6 | W/R/S |
| Fimbristylis squarrosa Vahl | 7 |  |  |  | 338.6 | 27041.9 | 47.6 | 7.1 | 3 | W/R/S |
| Fimbristylis stauntonii Debeaux & Franch. ex Debeaux | 3 |  |  |  | 223.5 | 14882.0 | 169.8 | 1.3 | 3 | N/B/S |
| Fimbristylis tristachya var. subbispicata (Nees & Meyen) T.Koyama | 7 |  |  |  | 223.6 | 10194.7 | 30.4 | 7.4 | 4 | N/B/L |
| Fimbristylis verrucifera (Maxim.) Makino | 2 |  |  |  | 237.0 | NA | 237.0 | 1.0 | 2 | N/R/S |
| Firmiana simplex (L.) W.F.Wight | 14 |  |  |  | 249.4 | 24451.3 | 25.8 | 9.7 | 4 | N/B/L |
| Forsythia koreana (Rehder) Nakai | 110 |  | V |  | 441.8 | 91127.7 | 14.7 | 30.1 | 9 | W/B/L |
| Fragaria ananassa Duch. | 5 |  |  |  | 330.3 | 15655.1 | 57.6 | 5.7 | 4 | W/R/S |
| Fraxinus mandshurica Rupr. | 25 |  |  |  | 287.8 | 39187.5 | 22.7 | 12.7 | 6 | N/B/L |
| Fraxinus rhynchophylla Hance | 308 |  |  |  | 450.6 | 95646.8 | 8.3 | 54.2 | 11 | W/B/L |
| Fraxinus sieboldiana Blume | 254 |  |  |  | 417.4 | 80229.2 | 8.1 | 51.7 | 9 | W/B/L |
| Gagea hiensis Pascher | 2 |  |  |  | 216.8 | NA | 216.8 | 1.0 | 1 | N/R/S |
| Gagea lutea (L.) KerGawl. | 16 |  |  |  | 345.8 | 49755.5 | 28.1 | 12.3 | 4 | W/R/S |
| Galeopsis bifida Boenn. | 2 |  |  |  | 5.7 | NA | 5.7 | 1.0 | 2 | N/R/S |
| Galinsoga ciliata (Raf.) S.F.Blake | 161 |  |  |  | 447.0 | 90362.3 | 10.3 | 43.4 | 9 | W/B/L |
| Galinsoga parviflora Cav. | 55 |  |  |  | 365.2 | 64257.5 | 15.9 | 23.0 | 6 | W/R/S |
| Galium boreale var. boreale L. | 9 |  |  |  | 304.0 | 23640.6 | 38.8 | 7.8 | 3 | N/B/L |
| Galium dahuricum var. dahuricum Turcz. | 69 |  |  |  | 391.1 | 84985.1 | 18.1 | 21.6 | 6 | W/R/S |
| Galium dahuricum var. tokyoense (Makino) Cufod. | 17 |  |  |  | 352.7 | 28691.7 | 21.0 | 16.8 | 5 | W/R/S |
| Galium gracilens (A.Gray) Makino | 16 |  |  |  | 364.7 | 37788.4 | 39.1 | 9.3 | 4 | W/R/S |
| Galium japonicum (Maxim.) Makino & Nakai | 4 |  |  |  | 0.6 | 0.1 | 0.2 | 2.8 | 1 | N/R/S |
| Galium kamtschaticum Steller ex Roem. & Schult. | 14 |  |  |  | 364.6 | 37868.7 | 40.5 | 9.0 | 2 | W/R/S |
| Galium kinuta Nakai & Hara | 42 |  |  |  | 309.1 | 40336.6 | 17.0 | 18.2 | 4 | N/B/L |
| Galium koreanum (Nakai) Nakai | 13 |  | V |  | 409.1 | 61087.5 | 56.6 | 7.2 | 4 | W/R/S |
| Galium paradoxum Maxim. | 9 |  |  |  | 113.9 | 2087.2 | 14.0 | 8.1 | 3 | N/B/L |
| Galium pogonanthum Franch. & Sav. | 106 |  |  |  | 443.1 | 76385.9 | 14.0 | 31.6 | 7 | W/B/L |
| Galium spurium var. echinospermon (Wallr.) Hayek | 237 |  |  |  | 446.2 | 97797.5 | 10.5 | 42.4 | 10 | W/B/L |
| Galium trachyspermum A.Gray | 120 |  |  |  | 473.7 | 83510.0 | 10.7 | 44.2 | 7 | W/B/L |
| Galium trifidum L. | 5 |  |  |  | 244.6 | 13713.5 | 78.9 | 3.1 | 3 | N/B/S |
| Galium trifloriforme Kom. | 16 |  |  |  | 286.9 | 15581.3 | 14.2 | 20.1 | 3 | N/B/L |
| Galium verum for. pusillum (Nakai) M.Park | 2 |  | V |  | 18.8 | NA | 18.8 | 1.0 | 2 | N/R/S |
| Galium verum var. asiaticum Nakai | 271 |  |  |  | 455.2 | 98577.1 | 8.8 | 51.9 | 11 | W/B/L |
| Gastrodia elata Blume | 17 |  |  | LC | 353.3 | 34632.8 | 26.6 | 13.3 | 3 | W/R/S |
| Gentiana scabra for. scabra Bunge | 132 |  |  |  | 423.8 | 77500.5 | 11.5 | 36.9 | 9 | W/B/L |
| Gentiana squarrosa var. microphylla Nakai | 3 |  | V |  | 34.2 | 41.9 | 14.5 | 2.4 | 1 | N/R/S |
| Gentiana squarrosa var. squarrosa Ledeb. | 53 |  |  |  | 439.5 | 64987.5 | 19.3 | 22.8 | 6 | W/R/S |
| Gentiana thunbergii (G.Don) Griseb. | 9 |  |  |  | 425.3 | 41713.8 | 33.0 | 12.9 | 3 | W/R/S |
| Gentiana triflora for. japonica (Kusn.) W.T.Lee & W.Paik | 5 |  |  |  | 316.0 | 3507.0 | 65.4 | 4.8 | 3 | W/R/S |
| Gentiana triflora var. japonica (Kusn.) H.Hara | 21 |  |  |  | 347.0 | 35936.6 | 18.4 | 18.8 | 3 | W/R/S |
| Gentiana zollingeri for. zollingeri Faw. | 143 |  |  |  | 445.9 | 94844.2 | 12.9 | 34.4 | 8 | W/B/L |
| Geranium carolinianum L. | 8 |  |  |  | 228.3 | 6005.2 | 11.2 | 20.4 | 5 | N/B/L |
| Geranium dahuricum DC. | 2 |  |  |  | 142.7 | NA | 142.7 | 1.0 | 1 | N/R/S |
| Geranium eriostemon Fisher ex DC. | 11 |  |  |  | 237.9 | 17038.6 | 25.7 | 9.2 | 4 | N/B/L |
| Geranium knuthii Nakai | 8 |  | V |  | 273.2 | 8521.5 | 33.9 | 8.1 | 5 | N/B/L |
| Geranium koraiense Nakai | 11 |  | V |  | 321.8 | 38662.5 | 25.8 | 12.5 | 4 | W/R/S |
| Geranium koreanum Kom. | 27 |  | V |  | 405.4 | 55176.6 | 19.1 | 21.3 | 3 | W/R/S |
| Geranium koreanum var. hirsutum Nakai | 2 |  | V |  | 85.1 | NA | 85.1 | 1.0 | 2 | N/R/S |
| Geranium krameri Franch. & Sav. | 24 |  |  |  | 372.3 | 51393.6 | 30.9 | 12.0 | 5 | W/R/S |
| Geranium sibiricum L. | 116 |  |  |  | 449.1 | 92050.1 | 13.0 | 34.6 | 9 | W/B/L |
| Geranium thunbergii Siebold & Zucc. | 297 |  |  |  | 453.1 | 95956.8 | 8.8 | 51.7 | 9 | W/B/L |
| Geranium wilfordii Maxim. | 63 |  |  |  | 368.5 | 73831.8 | 16.8 | 21.9 | 4 | W/R/S |
| Geum aleppicum Jacq. | 167 |  |  |  | 434.4 | 82112.0 | 10.5 | 41.4 | 9 | W/B/L |
| Geum japonicum Thunb. | 123 |  |  |  | 442.8 | 86486.8 | 12.2 | 36.3 | 5 | W/R/L |
| Ginkgo biloba L. | 60 |  |  |  | 415.3 | 72155.3 | 16.3 | 25.4 | 7 | W/B/S |
| Girardinia diversifolia subsp. Suborbiculata (Link) Friis (C. J. Chen) C. J. Chen & Friis | 7 |  |  |  | 349.0 | 37876.0 | 77.8 | 4.5 | 2 | W/R/S |
| Glechoma grandis (A.Gray) Kuprian. | 14 |  |  |  | 303.4 | 30867.7 | 26.5 | 11.5 | 6 | N/B/L |
| Gleditsia japonica Miq. | 12 |  |  |  | 282.9 | 37177.1 | 34.5 | 8.2 | 6 | N/B/L |
| Glehnia littoralis F.Schmidt ex Miq. | 3 |  |  |  | 254.7 | 98.4 | 86.1 | 3.0 | 3 | N/B/S |
| Glyceria ischyroneura Steud. | 3 |  |  |  | 270.3 | 1581.1 | 119.8 | 2.3 | 2 | N/R/S |
| Glyceria leptolepis Ohwi | 4 |  |  |  | 244.9 | 6508.9 | 80.3 | 3.0 | 2 | N/R/S |
| Glycine max (L.) Merr. | 5 |  |  |  | 287.0 | 14023.2 | 74.9 | 3.8 | 3 | N/B/S |
| Glycine soja Siebold & Zucc. | 225 |  |  |  | 457.3 | 94913.8 | 9.9 | 46.2 | 11 | W/B/L |
| Gnaphalium affine D.Don | 120 |  |  |  | 421.2 | 78371.3 | 12.4 | 33.9 | 8 | W/B/L |
| Gnaphalium hypoleucum DC. | 4 |  |  |  | 0.7 | 0.1 | 0.2 | 3.7 | 2 | N/R/S |
| Gnaphalium japonicum Thunb. | 16 |  |  |  | 388.3 | 48041.1 | 37.7 | 10.3 | 6 | W/R/S |
| Gnaphalium uliginosum L. | 3 |  |  |  | 254.7 | 18632.8 | 187.0 | 1.4 | 2 | N/R/S |
| Goodyera repens (L.) R.Br. | 3 |  |  | VU | 94.4 | 223.1 | 34.2 | 2.8 | 3 | N/B/S |
| Goodyera schlechtendaliana Rchb.f. | 7 |  |  | LC | 95.3 | 3112.3 | 24.4 | 3.9 | 3 | N/B/S |
| Grewia biloba Bunge | 17 |  |  |  | 207.7 | 12304.6 | 18.0 | 11.6 | 5 | N/B/L |
| Grewia parviflora Bunge | 18 |  |  |  | 301.4 | 40797.6 | 13.3 | 22.7 | 4 | N/B/L |
| Gymnaster koraiensis (Nakai) Kitam. | 49 |  | V |  | 361.0 | 68743.8 | 18.5 | 19.5 | 9 | W/B/S |
| Gymnocarpium robertianum (Hoffm.) Newman | 2 |  |  |  | 12.8 | NA | 12.8 | 1.0 | 2 | N/R/S |
| Gynostemma pentaphyllum (Thunb.) Makino | 21 |  |  |  | 330.6 | 37650.8 | 21.2 | 15.6 | 4 | W/R/S |
| Gypsophila oldhamiana Miq. | 27 |  |  |  | 399.8 | 57568.6 | 18.4 | 21.8 | 4 | W/R/S |
| Habenaria linearifolia for. linearifolia Maxim. | 3 |  |  |  | 193.4 | 9564.5 | 132.3 | 1.5 | 2 | N/R/S |
| Haloragis micrantha (Thunb.) R.Br. ex Siebold & Zucc. | 45 |  |  |  | 327.5 | 52562.5 | 15.6 | 21.0 | 7 | W/B/S |
| Hanabusaya asiatica (Nakai) Nakai | 10 |  | V | LC | 75.3 | 1933.7 | 6.9 | 10.9 | 4 | N/B/L |
| Hedera rhombea (Miq.) Bean | 36 |  |  |  | 236.8 | 23394.7 | 11.6 | 20.4 | 4 | N/B/L |
| Hedyotis diffusa | 3 |  |  |  | 20.0 | 0.2 | 9.1 | 2.2 | 1 | N/R/S |
| Helianthus annuus L. | 2 |  |  |  | 278.9 | NA | 278.9 | 1.0 | 1 | N/R/S |
| Helianthus tuberosus L. | 64 |  |  |  | 461.3 | 85073.0 | 15.4 | 29.9 | 6 | W/R/L |
| Heloniopsis koreana S. Fuse, N. S. Lee & M. N. Tamura | 66 |  |  |  | 247.0 | 21867.6 | 9.9 | 25.0 | 5 | N/B/L |
| Hemarthria sibirica (Gand.) Ohwi | 17 |  |  |  | 277.5 | 31836.3 | 33.7 | 8.2 | 4 | N/B/L |
| Hemerocallis coreana Nakai | 44 |  |  |  | 287.3 | 47821.9 | 18.8 | 15.3 | 6 | N/B/L |
| Hemerocallis dumortieri Morren | 51 |  |  |  | 398.5 | 69579.1 | 16.2 | 24.6 | 7 | W/B/S |
| Hemerocallis fulva (L.) L. | 192 |  |  |  | 438.8 | 94634.8 | 10.2 | 42.9 | 9 | W/B/L |
| Hemerocallis fulva for. kwanso (Regel) Kitam. | 17 |  |  |  | 369.1 | 58086.5 | 25.6 | 14.4 | 7 | W/B/S |
| Hemerocallis fulva var. longituba (Miq.) Maxim. | 2 |  |  |  | 79.0 | NA | 79.0 | 1.0 | 2 | N/R/S |
| Hemerocallis hakuunensis Nakai | 11 |  | V |  | 173.6 | 12400.1 | 23.3 | 7.5 | 4 | N/B/L |
| Hemerocallis middendorffii Trautv. & C.A.Mey. | 5 |  |  |  | 247.3 | 11145.8 | 64.1 | 3.9 | 3 | N/B/S |
| Hemerocallis minor Mill. | 39 |  |  |  | 414.8 | 82930.0 | 25.6 | 16.2 | 7 | W/B/S |
| Hemerocallis thunbergii Baker | 11 |  |  |  | 433.8 | 36195.4 | 44.5 | 9.8 | 3 | W/R/S |
| Hemiptelea davidii (Hance) Planch. | 73 |  |  |  | 439.0 | 76448.7 | 12.6 | 35.0 | 8 | W/B/L |
| Hemistepa lyrata Bunge | 328 |  |  |  | 453.9 | 98469.2 | 9.0 | 50.6 | 13 | W/B/L |
| Hepatica asiatica Nakai | 226 |  |  |  | 457.0 | 99783.8 | 9.3 | 49.1 | 10 | W/B/L |
| Hepatica insularis Nakai | 26 |  | V |  | 382.6 | 59019.0 | 25.8 | 14.8 | 5 | W/R/S |
| Heracleum moellendorffii Hance | 95 |  |  |  | 413.9 | 73294.7 | 13.2 | 31.3 | 8 | W/B/L |
| Heteropappus hispidus (Thunb.) Less. | 35 |  |  |  | 421.6 | 85181.2 | 17.2 | 24.5 | 8 | W/B/S |
| Heteropappus meyendorfii (Regel & Maack) Kom. & Klob.-Alisova | 287 |  |  |  | 468.9 | 94140.3 | 8.6 | 54.7 | 9 | W/B/L |
| Hibiscus mutabilis L. | 2 |  |  |  | 128.1 | NA | 128.1 | 1.0 | 2 | N/R/S |
| Hibiscus syriacus L. | 19 |  |  |  | 366.8 | 64407.5 | 32.0 | 11.5 | 7 | W/B/S |
| Hibiscus trionum L. | 11 |  |  |  | 233.8 | 14357.5 | 25.1 | 9.3 | 4 | N/B/L |
| Hieracium umbellatum L. | 137 |  |  |  | 467.5 | 89249.8 | 13.1 | 35.7 | 9 | W/B/L |
| Hierochloe odorata (L.) P.Beauv. | 54 |  |  |  | 387.9 | 60611.0 | 17.1 | 22.7 | 8 | W/B/S |
| Hololeion maximowiczii Kitam. | 12 |  |  | NT | 348.4 | 58595.9 | 68.2 | 5.1 | 5 | W/R/S |
| Hordeum vulgare var. hexastichon (L.) Asch. | 4 |  |  |  | 262.6 | 4493.5 | 81.8 | 3.2 | 3 | N/B/S |
| Hosta capitata (Koidz.) Nakai | 118 |  |  |  | 404.8 | 58501.5 | 9.3 | 43.6 | 7 | W/B/L |
| Hosta clausa Nakai | 2 |  |  |  | 259.0 | NA | 259.0 | 1.0 | 1 | N/R/S |
| Hosta longipes (Franch. & Sav.) Matsum. | 131 |  |  |  | 476.1 | 94828.9 | 10.9 | 43.9 | 7 | W/B/L |
| Hosta longisima Honda | 3 |  |  |  | 461.5 | 13954.9 | 197.7 | 2.3 | 3 | W/R/S |
| Hosta minor (Baker) Nakai | 25 |  | V |  | 400.6 | 65846.9 | 25.2 | 15.9 | 3 | W/R/S |
| Hosta plantaginea (Lam.) Aschers. | 6 |  |  |  | 319.3 | 28875.4 | 83.6 | 3.8 | 3 | W/R/S |
| Houttuynia cordata Thunb. | 5 |  |  |  | 183.3 | 9109.3 | 52.5 | 3.5 | 4 | N/B/S |
| Hovenia dulcis Thunb. ex Murray | 16 |  |  |  | 311.1 | 42685.6 | 30.4 | 10.2 | 4 | N/B/L |
| Humulus japonicus Sieboid & Zucc. | 381 |  |  |  | 438.1 | 101002.5 | 8.0 | 55.0 | 11 | W/B/L |
| Huperzia miyoshiana (Makino) Ching | 2 |  |  |  | 137.7 | NA | 137.7 | 1.0 | 2 | N/R/S |
| Huperzia selago (L.) Bernh. ex Schrank & Mart. | 2 |  |  | VU | 24.6 | NA | 24.6 | 1.0 | 1 | N/R/S |
| Hydrangea macrophylla (Thunb.) Ser. | 6 |  |  |  | 246.1 | 29393.1 | 70.6 | 3.5 | 3 | N/B/S |
| Hydrangea macrophylla var. acuminata (Siebold & Zucc.) Makino | 219 |  |  |  | 396.2 | 69334.0 | 8.0 | 49.8 | 8 | W/B/L |
| Hydrilla verticillata (L.f.) Royle | 15 |  |  |  | 352.3 | 49592.8 | 35.5 | 9.9 | 7 | W/B/S |
| Hydrocharis dubia (Blume) Backer | 2 |  |  |  | 159.5 | NA | 159.5 | 1.0 | 1 | N/R/S |
| Hydrocotyle javanica Thunb. | 3 |  |  |  | 210.5 | 11230.3 | 149.2 | 1.4 | 2 | N/R/S |
| Hydrocotyle maritima Honda | 14 |  |  |  | 221.3 | 23138.0 | 28.7 | 7.7 | 5 | N/B/L |
| Hydrocotyle ramiflora Maxim. | 35 |  |  |  | 339.7 | 50069.4 | 18.7 | 18.2 | 8 | W/B/S |
| Hydrocotyle sibthorpioides Lam. | 22 |  |  |  | 312.4 | 44082.2 | 20.7 | 15.1 | 7 | N/B/L |
| Hylomecon vernalis Maxim. | 107 |  |  |  | 388.2 | 72856.3 | 13.1 | 29.6 | 6 | W/R/L |
| Hylotelephium erythrostictum (Miq.) H.Ohba | 30 |  |  |  | 415.6 | 76204.8 | 23.4 | 17.8 | 4 | W/R/S |
| Hylotelephium spectabile (Boreau) H.Ohba | 10 |  |  |  | 418.2 | 60114.5 | 54.8 | 7.6 | 4 | W/R/S |
| Hylotelephium telephium (L.) H.Ohba | 2 |  |  |  | 224.0 | NA | 224.0 | 1.0 | 1 | N/R/S |
| Hylotelephium verticillatum (L.) H.Ohba | 32 |  |  |  | 315.2 | 53097.3 | 23.8 | 13.2 | 5 | W/R/S |
| Hylotelephium viviparum (Maxim.) H.Ohba | 28 |  |  |  | 333.4 | 42110.3 | 18.6 | 17.9 | 5 | W/R/S |
| Hymenophyllum polyanthos (Sw.) Sw. | 3 |  |  |  | 245.7 | 19713.2 | 196.9 | 1.2 | 2 | N/R/S |
| Hypericum ascyron L. | 274 |  |  |  | 448.3 | 89265.2 | 9.2 | 49.0 | 8 | W/B/L |
| Hypericum ascyron var. longistylum Maxim. | 3 |  |  |  | 180.9 | 7520.5 | 116.1 | 1.6 | 2 | N/R/S |
| Hypericum atenuatum var. confertissium (Nakai) T.B.Lee | 3 |  | V |  | 212.3 | 2875.4 | 103.8 | 2.0 | 3 | N/B/S |
| Hypericum attenuatum Choisy | 12 |  |  | NT | 314.4 | 38461.0 | 43.9 | 7.2 | 3 | N/B/L |
| Hypericum erectum Thunb. | 317 |  |  |  | 495.4 | 104358.3 | 8.5 | 58.1 | 11 | W/B/L |
| Hypericum galioides Lam. | 2 |  |  |  | 316.3 | NA | 316.3 | 1.0 | 2 | W/R/S |
| Hypericum japonicum Thunb. | 37 |  |  |  | 454.1 | 89973.7 | 25.0 | 18.2 | 8 | W/B/S |
| Hypericum laxum (Blume) Koidz. | 40 |  |  |  | 487.9 | 72574.9 | 21.4 | 22.8 | 8 | W/B/S |
| Hypericum oliganthum Franch. & Sav. | 2 |  |  | EN | 93.1 | NA | 93.1 | 1.0 | 2 | N/R/S |
| Hypochaeris ciliata (Thunb.) Makino | 3 |  |  |  | 78.4 | 295.3 | 31.7 | 2.5 | 3 | N/B/S |
| Hypochaeris radicata L. | 13 |  |  |  | 369.3 | 48875.0 | 45.0 | 8.2 | 5 | W/R/S |
| Hypodematium glanduloso-pilosum (Tagawa) Ohwi | 7 |  |  | NT | 248.2 | 6841.7 | 40.5 | 6.1 | 2 | N/R/S |
| Hystrix longearistata (Hack.) Honda | 3 |  |  |  | 194.7 | 572.4 | 69.4 | 2.8 | 2 | N/R/S |
| Idesia polycarpa Maxim. | 7 |  |  |  | 172.3 | 7401.6 | 36.4 | 4.7 | 3 | N/B/S |
| Ilex cornuta Lindl. & Paxton | 8 |  |  | LC | 208.9 | 8926.8 | 26.9 | 7.8 | 3 | N/B/L |
| Ilex crenata var. crenata Thunb. | 4 |  |  |  | 56.9 | 738.5 | 19.8 | 2.9 | 3 | N/B/S |
| Ilex integra Thunb. | 6 |  |  |  | 328.2 | 3306.7 | 12.2 | 27.0 | 3 | W/R/S |
| Ilex macropoda for. macropoda Miq. | 76 |  |  |  | 360.9 | 50191.0 | 11.5 | 31.4 | 4 | W/R/L |
| Ilex macropoda for. pseudo-macropoda (Loes.) H.Hara | 2 |  |  |  | 169.0 | NA | 169.0 | 1.0 | 1 | N/R/S |
| Impatiens atrosanguinea (Nakai) B.U.Oh & Y.P.Hong | 4 |  | V |  | 12.5 | 64.5 | 7.1 | 1.8 | 2 | N/R/S |
| Impatiens balsamina L. | 15 |  |  |  | 394.0 | 45439.1 | 32.8 | 12.0 | 4 | W/R/S |
| Impatiens kojensis Y.N.Lee | 4 |  |  |  | 426.8 | 24183.5 | 80.1 | 5.3 | 2 | W/R/S |
| Impatiens nolitangere var. nolitangere L. | 128 |  |  |  | 415.7 | 64728.6 | 10.6 | 39.3 | 10 | W/B/L |
| Impatiens textori var. koreana Nakai | 16 |  |  |  | 334.5 | 33485.4 | 19.1 | 17.5 | 4 | W/R/S |
| Impatiens textori var. textori Miquel | 476 |  |  |  | 491.6 | 98159.3 | 7.2 | 68.6 | 10 | W/B/L |
| Imperata cylindrica var. koenigii (Retz.) Pilg. | 115 |  |  |  | 433.2 | 95457.9 | 13.6 | 31.8 | 9 | W/B/L |
| Indigofera kirilowii Maxim. ex Palib. | 354 |  |  |  | 443.7 | 90546.7 | 6.8 | 65.2 | 11 | W/B/L |
| Indigofera koreana Ohwi | 37 |  |  |  | 260.5 | 27595.9 | 9.9 | 26.3 | 4 | N/B/L |
| Indigofera pseudotinctoria Matsum. | 74 |  |  |  | 480.8 | 90154.5 | 18.4 | 26.1 | 10 | W/B/S |
| Inula britannica var. japonica (Thunb.) Franch. & Sav. | 45 |  |  |  | 382.3 | 65447.5 | 18.1 | 21.1 | 7 | W/B/S |
| Inula britannica var. linariifolia (Turcz.) Regel | 10 |  |  |  | 321.0 | 30596.6 | 53.0 | 6.1 | 4 | W/R/S |
| Inula salicina var. asiatica Kitam. | 18 |  |  |  | 332.0 | 52713.2 | 35.5 | 9.4 | 6 | W/R/S |
| Ipomoea hederacea var. hederacea Jacq. | 26 |  |  |  | 417.3 | 49536.5 | 28.6 | 14.6 | 6 | W/R/S |
| Ipomoea hederacea var. integriuscula A.Gray | 12 |  |  |  | 417.3 | 63237.5 | 59.0 | 7.1 | 3 | W/R/S |
| Ipomoea lacunosa L. | 11 |  |  |  | 351.7 | 24323.8 | 35.4 | 9.9 | 6 | W/R/S |
| Ipomoea purpurea Roth | 72 |  |  |  | 420.2 | 79956.2 | 14.4 | 29.2 | 9 | W/B/L |
| Iris ensata var. spontanea (Makino) Nakai | 19 |  |  |  | 405.9 | 57506.9 | 41.0 | 9.9 | 4 | W/R/S |
| Iris koreana Nakai | 2 |  | V | VU | 110.3 | NA | 110.3 | 1.0 | 2 | N/R/S |
| Iris lactea var. chinensis (Fisch.) Koidz. | 11 |  |  |  | 439.5 | 56104.9 | 69.2 | 6.3 | 8 | W/B/S |
| Iris minutoaurea Makino | 40 |  |  | LC | 380.2 | 51452.6 | 20.3 | 18.7 | 5 | W/R/S |
| Iris odaesanensis Y.N.Lee | 14 |  | V | LC | 227.9 | 7376.9 | 8.2 | 27.8 | 4 | N/B/L |
| Iris pseudoacorus L. | 9 |  |  |  | 341.5 | 33681.6 | 62.0 | 5.5 | 5 | W/R/S |
| Iris rossii var. latifolia J.K.Sim & Y.S.Kim | 11 |  | V |  | 15.4 | 26.4 | 0.5 | 29.9 | 3 | N/B/L |
| Iris rossii var. rossii Baker | 247 |  |  |  | 444.6 | 97102.2 | 9.2 | 48.3 | 10 | W/B/L |
| Iris ruthenica var. nana Ker Gawl. Maxim. | 11 |  |  |  | 277.4 | 28675.5 | 34.1 | 8.1 | 3 | N/B/L |
| Iris sanguinea Donn ex Horn | 92 |  |  |  | 424.3 | 74478.1 | 15.4 | 27.5 | 8 | W/B/S |
| Iris uniflora var. caricina Kitag. | 2 |  |  | LC | 239.1 | NA | 239.1 | 1.0 | 1 | N/R/S |
| Isachne globosa (Thunb.) Kuntze | 32 |  |  |  | 437.4 | 68841.0 | 28.1 | 15.6 | 8 | W/B/S |
| Ischaemum antephoroides (Steud.) Miq. | 3 |  |  |  | 325.5 | 10360.4 | 148.4 | 2.2 | 1 | W/R/S |
| Ischaemum crassipes (Steud.) Thell. | 11 |  |  |  | 326.0 | 35647.3 | 36.5 | 8.9 | 4 | W/R/S |
| Isodon excisus (Maxim.) Kudo | 98 |  |  |  | 470.8 | 74825.2 | 9.1 | 51.5 | 6 | W/R/L |
| Isodon inflexus (Thunb.) Kudo | 469 |  |  |  | 495.5 | 105250.2 | 6.7 | 73.7 | 11 | W/B/L |
| Isoetes coreana Y.H.Chung & H.G.Choi | 2 |  | V | VU | 216.8 | NA | 216.8 | 1.0 | 2 | N/R/S |
| Isopyrum manshuricum (Kom.) Kom. | 6 |  |  | LC | 292.2 | 18871.6 | 44.2 | 6.6 | 4 | N/B/L |
| Isopyrum raddeanum (Regel) Maxim. | 10 |  |  |  | 106.4 | 4663.0 | 17.7 | 6.0 | 3 | N/B/S |
| Ixeridium dentatum (Thunb. ex Mori) Tzvelev | 340 |  |  |  | 463.6 | 97230.6 | 8.1 | 57.3 | 10 | W/B/L |
| Ixeridium dentatum for. albiflora (Makino) H.Hara | 32 |  |  |  | 382.4 | 51481.1 | 24.2 | 15.8 | 7 | W/B/S |
| Ixeris chinensis (Thunb.) Nakai | 25 |  |  |  | 413.6 | 69620.7 | 30.7 | 13.5 | 5 | W/R/S |
| Ixeris debilis (Thunb.) A.Gray | 34 |  |  |  | 428.7 | 76306.1 | 24.3 | 17.6 | 6 | W/R/S |
| Ixeris polycephala Cass. | 112 |  |  |  | 420.9 | 83431.0 | 13.2 | 31.8 | 10 | W/B/L |
| Ixeris repens (L.) A.Gray | 5 |  |  |  | 236.6 | 2131.2 | 23.5 | 10.1 | 2 | N/R/L |
| Ixeris stolonifera A.Gray | 35 |  |  |  | 387.5 | 54871.7 | 19.8 | 19.5 | 8 | W/B/S |
| Ixeris strigosa (H.Lev. & Vaniot) J.H.Pak & Kawano | 114 |  |  |  | 469.6 | 94231.5 | 12.9 | 36.5 | 9 | W/B/L |
| Ixeris tamagawaensis (Makino) Kitam. | 9 |  |  |  | 266.2 | 20131.2 | 30.4 | 8.7 | 4 | N/B/L |
| Jeffersonia dubia (Maxim.) Benth. & Hook.f. ex Baker & S.Moore | 6 |  |  | NT | 292.1 | 30356.8 | 83.4 | 3.5 | 3 | N/B/S |
| Juglans mandshurica var. mandshurica for. mandshurica Maxim. | 85 |  |  |  | 388.5 | 66990.6 | 11.3 | 34.4 | 7 | W/B/L |
| Juglans sinensis Dode | 18 |  |  |  | 336.8 | 31397.0 | 28.1 | 12.0 | 4 | W/R/S |
| Juncus alatus Franch. & Sav. | 8 |  |  |  | 294.0 | 24080.0 | 61.6 | 4.8 | 4 | N/B/S |
| Juncus bufonius L. | 4 |  |  |  | 304.0 | 14117.8 | 104.0 | 2.9 | 2 | N/R/S |
| Juncus diastrophanthus Buchenau | 14 |  |  |  | 465.4 | 34375.2 | 32.1 | 14.5 | 4 | W/R/S |
| Juncus effusus var. decipiens Buchenau | 282 |  |  |  | 495.2 | 103050.2 | 8.3 | 59.5 | 10 | W/B/L |
| Juncus gracillimus (Buchenau) V.I.Krecz. & Gontsch. | 6 |  |  |  | 174.6 | 15648.5 | 59.4 | 2.9 | 4 | N/B/S |
| Juncus haenkei E.Mey. | 2 |  |  |  | 171.3 | NA | 171.3 | 1.0 | 1 | N/R/S |
| Juncus krameri Franch. & Sav. | 13 |  |  |  | 380.4 | 36720.4 | 45.5 | 8.4 | 6 | W/R/S |
| Juncus leschenaultii J.Gay | 17 |  |  |  | 325.3 | 39945.5 | 35.2 | 9.2 | 5 | W/R/S |
| Juncus papillosus Franch. & Sav. | 59 |  |  |  | 371.8 | 73503.1 | 17.7 | 21.1 | 7 | W/B/S |
| Juncus setchuensis var. effusoides Buchenau | 4 |  |  |  | 410.6 | 22692.1 | 105.8 | 3.9 | 3 | W/R/S |
| Juncus tenuis Willd. | 75 |  |  |  | 391.2 | 75279.5 | 14.4 | 27.2 | 6 | W/R/S |
| Juncus wallichianus Laharpe | 15 |  |  |  | 443.9 | 69892.4 | 45.1 | 9.8 | 6 | W/R/S |
| Juniperus chinensis L. | 25 |  |  | VU | 392.0 | 67839.5 | 33.0 | 11.9 | 6 | W/R/S |
| Juniperus chinensis var. procumbens (Siebold) Endl. | 2 |  |  |  | 0.4 | NA | 0.4 | 1.0 | 1 | N/R/S |
| Juniperus chinensis var. sargentii Henry | 2 |  |  |  | 169.1 | NA | 169.1 | 1.0 | 2 | N/R/S |
| Juniperus rigida Siebold & Zucc. | 382 |  |  |  | 437.5 | 94518.5 | 6.8 | 64.8 | 11 | W/B/L |
| Justicia procumbens L. | 244 |  |  |  | 413.2 | 85051.5 | 9.2 | 44.7 | 10 | W/B/L |
| Kadsura japonica (L.) Dunal | 4 |  |  |  | 50.8 | 592.5 | 20.4 | 2.5 | 3 | N/B/S |
| Kalimeris associata (Kitag.) Kitag. | 4 |  |  |  | 104.7 | 1515.0 | 31.5 | 3.3 | 2 | N/R/S |
| Kalimeris incisa (Fisch.) DC | 92 |  |  |  | 403.3 | 70981.7 | 13.8 | 29.2 | 11 | W/B/L |
| Kalimeris integrifolia Turcz. ex DC. | 60 |  |  |  | 350.2 | 73747.6 | 16.5 | 21.2 | 7 | W/B/S |
| Kalimeris pinnatifida (Maxim.) Kitam. | 32 |  |  |  | 360.7 | 55678.5 | 19.2 | 18.8 | 5 | W/R/S |
| Kalimeris yomena Kitam. | 158 |  |  |  | 438.1 | 95398.7 | 11.4 | 38.4 | 11 | W/B/L |
| Kalopanax septemlobus (Thunb. ex Murray) Koidz. | 154 |  |  |  | 452.3 | 88365.6 | 10.7 | 42.3 | 8 | W/B/L |
| Kalopanax septemlobus var. maximowiczi (VanHoutte) Hand.-Mazz. | 11 |  |  |  | 222.4 | 10950.3 | 33.0 | 6.7 | 3 | N/B/L |
| Kerria japonica (L.) DC. | 12 |  |  |  | 331.3 | 33529.0 | 32.1 | 10.3 | 4 | W/R/S |
| Kerria japonica for. pleniflora (Witte) Rehder | 23 |  |  |  | 384.9 | 43496.4 | 27.6 | 13.9 | 5 | W/R/S |
| Kobresia bellardii (All.) Degl. | 3 |  |  |  | 176.3 | 9760.9 | 137.0 | 1.3 | 2 | N/R/S |
| Kochia scoparia var. scoparia (L.) Schrad. | 4 |  |  |  | 233.9 | 11839.4 | 97.2 | 2.4 | 3 | N/B/S |
| Koeleria cristata (L.) Pers. | 21 |  |  |  | 342.6 | 44405.4 | 36.3 | 9.4 | 6 | W/R/S |
| Koelreuteria paniculata Laxmann | 22 |  |  |  | 428.3 | 80458.5 | 35.3 | 12.1 | 6 | W/R/S |
| Kummerowia stipulacea (Maxim.) Makino | 49 |  |  |  | 459.5 | 84937.1 | 23.4 | 19.6 | 8 | W/B/S |
| Kummerowia striata (Thunb. ex Murray) Schindl. | 287 |  |  |  | 486.2 | 100091.5 | 9.9 | 48.9 | 9 | W/B/L |
| Kyllinga brevifolius (Rottb.) Hassk. | 101 |  |  |  | 452.1 | 88488.6 | 15.1 | 30.0 | 8 | W/B/L |
| Lactuca indica for. indivisa (Makino) Hara | 56 |  |  |  | 371.1 | 73188.9 | 17.0 | 21.8 | 7 | W/B/S |
| Lactuca indica var. laciniata (Kuntze) H.Hara | 399 |  |  |  | 479.5 | 104959.5 | 8.2 | 58.4 | 12 | W/B/L |
| Lactuca raddeana Maxim. | 139 |  |  |  | 436.3 | 89054.0 | 13.0 | 33.5 | 8 | W/B/L |
| Lactuca scariola L. | 9 |  |  |  | 368.3 | 65144.6 | 79.3 | 4.6 | 4 | W/R/S |
| Lactuca triangulata Maxim. | 54 |  |  |  | 380.6 | 67958.0 | 18.6 | 20.4 | 7 | W/B/S |
| Lagerstroemia indica L. | 33 |  |  |  | 438.9 | 69638.4 | 20.0 | 21.9 | 6 | W/R/S |
| Lamium album var. barbatum (Siebold & Zucc.) Franch. & Sav. | 156 |  |  |  | 462.5 | 88672.3 | 11.3 | 40.9 | 8 | W/B/L |
| Lamium amplexicaule L. | 244 |  |  |  | 433.4 | 95219.4 | 9.6 | 45.1 | 10 | W/B/L |
| Lamium purpureum L. | 4 |  |  |  | 247.2 | 11372.7 | 108.1 | 2.3 | 3 | N/B/S |
| Laportea bulbifera (Siebold & Zucc.) Wedd. | 34 |  |  |  | 342.4 | 49882.3 | 16.4 | 20.9 | 5 | W/R/S |
| Lapsanastrum apogonoides (Maxim.) J.H.Pak & K.Bremer | 4 |  |  |  | 320.2 | 27583.2 | 112.3 | 2.9 | 2 | W/R/S |
| Larix gmelinii var. olgensis (Rupr.) Kuzen. (A. Henry) Ostenf. & Syrach | 13 |  |  |  | 197.4 | 17331.8 | 25.0 | 7.9 | 4 | N/B/L |
| Larix kaempferi (Lamb.) Carriere | 152 |  |  |  | 350.3 | 67600.6 | 10.7 | 32.6 | 8 | W/B/L |
| Lathyrus davidii Hance | 156 |  |  |  | 433.3 | 74471.1 | 10.2 | 42.6 | 8 | W/B/L |
| Lathyrus japonicus Willd. | 6 |  |  |  | 374.0 | 46206.4 | 106.9 | 3.5 | 4 | W/R/S |
| Lathyrus quinquenervius (Miq.) Litv. | 2 |  |  |  | 59.5 | NA | 59.5 | 1.0 | 2 | N/R/S |
| Lathyrus vaniotii H.Lev. | 18 |  |  |  | 296.6 | 22810.5 | 22.3 | 13.3 | 5 | N/B/L |
| Leersia japonica Makino | 20 |  |  |  | 386.5 | 66092.5 | 37.0 | 10.4 | 7 | W/B/S |
| Leersia oryzoides var. oryzoides (L.) Sw. | 2 |  |  |  | 190.6 | NA | 190.6 | 1.0 | 2 | N/R/S |
| Leibnitzia anandria (L.) Turcz. | 291 |  |  |  | 460.9 | 98261.5 | 8.0 | 57.5 | 9 | W/B/L |
| Lemmaphyllum microphyllum C.Presl | 4 |  |  |  | 200.2 | 9565.2 | 69.7 | 2.9 | 3 | N/B/S |
| Lemna paucicostata (L.) Hegelm. | 25 |  |  |  | 329.5 | 53150.0 | 17.7 | 18.6 | 7 | W/B/S |
| Leontopodium japonicum Miq. | 3 |  |  |  | 33.8 | 252.9 | 21.5 | 1.6 | 2 | N/R/S |
| Leontopodium leontopodioides (Willd.) Beauverd | 4 |  |  |  | 131.2 | 3942.9 | 34.9 | 3.8 | 2 | N/R/S |
| Leonurus japonicus Houtt. | 321 |  |  |  | 474.7 | 104262.4 | 9.1 | 52.3 | 12 | W/B/L |
| Leonurus macranthus Maxim. | 90 |  |  |  | 442.2 | 72200.2 | 14.2 | 31.0 | 9 | W/B/L |
| Lepidium apetalum Willd. | 206 |  |  |  | 461.6 | 101566.3 | 12.0 | 38.4 | 10 | W/B/L |
| Lepidium virginicum L. | 64 |  |  |  | 425.1 | 74418.2 | 15.4 | 27.6 | 7 | W/B/S |
| Lepisorus onoei (Franch. & Sav.) Ching | 3 |  |  |  | 267.7 | 2853.3 | 101.8 | 2.6 | 2 | N/R/S |
| Lepisorus thunbergianus (Kaulf.) Ching | 23 |  |  |  | 361.5 | 60706.9 | 33.4 | 10.8 | 3 | W/R/S |
| Lepisorus ussuriensis (Regel & Maack) Ching | 85 |  |  |  | 371.1 | 70811.0 | 13.7 | 27.0 | 6 | W/R/S |
| Leptochloa chinensis (L.) Nees | 6 |  |  |  | 199.3 | 15726.6 | 39.4 | 5.1 | 3 | N/B/S |
| Lespedeza bicolor for. alba (Bean) Ohwi | 6 |  |  |  | 201.2 | 1724.8 | 36.4 | 5.5 | 5 | N/B/S |
| Lespedeza bicolor Turcz. | 484 |  |  |  | 495.3 | 105429.2 | 7.1 | 70.1 | 12 | W/B/L |
| Lespedeza cuneata G.Don | 287 |  |  |  | 480.3 | 101931.0 | 9.5 | 50.3 | 13 | W/B/L |
| Lespedeza cyrtobotrya Miq. | 360 |  |  |  | 485.4 | 99658.8 | 7.6 | 64.3 | 11 | W/B/L |
| Lespedeza daurica (Laxm.) Schindl. | 15 |  |  |  | 279.5 | 37876.8 | 24.5 | 11.4 | 5 | N/B/L |
| Lespedeza inschanica (Maxim.) Schindl. | 5 |  |  |  | 220.6 | 14682.6 | 79.8 | 2.8 | 3 | N/B/S |
| Lespedeza juncea (L.f.) Pers. | 18 |  |  |  | 338.2 | 53103.1 | 35.0 | 9.7 | 7 | W/B/S |
| Lespedeza macro-virgata Kitag. | 2 |  |  |  | 12.5 | NA | 12.5 | 1.0 | 2 | N/R/S |
| Lespedeza maritima Nakai | 30 |  | V |  | 354.7 | 44191.3 | 15.7 | 22.6 | 5 | W/R/S |
| Lespedeza maximowiczii C.K.Schneid. | 502 |  |  |  | 495.3 | 103332.9 | 6.5 | 75.9 | 10 | W/B/L |
| Lespedeza maximowiczii var. tomentella Nakai | 120 |  | V |  | 445.5 | 84745.1 | 11.3 | 39.4 | 9 | W/B/L |
| Lespedeza maximowiczii var. tricolor Nakai | 2 |  | V |  | 22.1 | NA | 22.1 | 1.0 | 2 | N/R/S |
| Lespedeza pilosa (Thunb.) Siebold & Zucc. | 34 |  |  |  | 325.5 | 41852.2 | 14.3 | 22.8 | 5 | W/R/S |
| Lespedeza thunbergii subsp. formosa (Vogel) H.Ohashi | 40 |  |  |  | 457.7 | 79134.7 | 21.1 | 21.7 | 5 | W/R/S |
| Lespedeza tomentosa (Thunb.) Siebold ex Maxim. | 45 |  |  |  | 478.0 | 72482.1 | 21.7 | 22.0 | 9 | W/B/S |
| Lespedeza virgata (Thunb.) DC. | 31 |  |  |  | 416.1 | 78737.1 | 28.0 | 14.8 | 5 | W/R/S |
| Lespedeza xchiisanensis | 2 |  |  |  | 0.7 | NA | 0.7 | 1.0 | 2 | N/R/S |
| Lespedeza xnakaii T.B.Lee | 23 |  |  |  | 369.9 | 53047.7 | 29.7 | 12.4 | 7 | W/B/S |
| Lespedeza xrogusta Nakai | 7 |  | V |  | 233.3 | 7191.5 | 31.2 | 7.5 | 4 | N/B/L |
| Lespedeza xschindleri T.B.Lee | 10 |  |  |  | 318.8 | 48598.5 | 67.7 | 4.7 | 4 | W/R/S |
| Ligularia fischeri (Ledeb.) Turcz. | 37 |  |  |  | 319.5 | 43490.3 | 16.7 | 19.2 | 5 | W/R/S |
| Ligularia stenocephala (Maxim.) Matsum. & Koidz. | 2 |  |  |  | 322.0 | NA | 322.0 | 1.0 | 2 | W/R/S |
| Ligustrum japonicum var. japonicum Thunb. | 24 |  |  |  | 404.0 | 55039.8 | 24.1 | 16.8 | 6 | W/R/S |
| Ligustrum obtusifolium Siebold & Zucc. | 503 |  |  |  | 446.4 | 99294.0 | 5.7 | 78.1 | 11 | W/B/L |
| Lilium amabile Palib. | 205 |  | V |  | 465.1 | 88305.8 | 9.2 | 50.5 | 8 | W/B/L |
| Lilium callosum Siebold & Zucc. | 7 |  |  | LC | 337.6 | 38055.3 | 66.7 | 5.1 | 3 | W/R/S |
| Lilium cernuum Kom. | 19 |  |  | LC | 247.5 | 14833.5 | 11.2 | 22.2 | 3 | N/B/L |
| Lilium concolor Salisb. | 19 |  |  |  | 389.4 | 64203.1 | 28.4 | 13.7 | 4 | W/R/S |
| Lilium distichum Nakai ex Kamib. | 87 |  |  | LC | 448.5 | 80342.0 | 12.8 | 34.9 | 7 | W/B/L |
| Lilium lancifolium Thunb. | 128 |  |  |  | 455.5 | 94078.2 | 13.1 | 34.8 | 8 | W/B/L |
| Lilium leichtlinii var. maximowiczii (Regel) Baker | 50 |  |  |  | 411.0 | 79674.5 | 16.8 | 24.4 | 6 | W/R/S |
| Lilium medeoloides A.Gray | 3 |  |  |  | 0.1 | 0.0 | 0.1 | 1.3 | 1 | N/R/S |
| Lilium tsingtauense Gilg | 239 |  |  |  | 430.5 | 92105.9 | 8.8 | 48.9 | 6 | W/R/L |
| Limnophila sessiliflora (Vahl) Blume | 2 |  |  | LC | 162.8 | NA | 162.8 | 1.0 | 2 | N/R/S |
| Limonium tetragonum (Thunb.) Bullock | 2 |  |  |  | 336.6 | NA | 336.6 | 1.0 | 1 | W/R/S |
| Linaria japonica Miq. | 6 |  |  |  | 145.6 | 1045.4 | 6.6 | 22.1 | 3 | N/B/L |
| Lindera erythrocarpa Makino | 344 |  |  |  | 439.3 | 83432.7 | 5.9 | 74.4 | 10 | W/B/L |
| Lindera glauca var. glauca (Siebold & Zucc.) Blume | 185 |  |  |  | 400.2 | 73267.3 | 7.7 | 51.7 | 7 | W/B/L |
| Lindera obtusiloba for. ovata T.B.Lee | 2 |  |  |  | 220.2 | NA | 220.2 | 1.0 | 2 | N/R/S |
| Lindera obtusiloba for. villosum Nakai | 9 |  |  |  | 268.1 | 12015.2 | 20.6 | 13.0 | 4 | N/B/L |
| Lindera obtusiloba var. obtusiloba Blume | 762 |  |  |  | 488.0 | 105876.7 | 5.0 | 96.8 | 13 | W/B/L |
| Lindera sericea (Siebold & Zucc.) Blume | 7 |  |  |  | 275.5 | 11852.5 | 48.9 | 5.6 | 3 | N/B/S |
| Lindernia dubia (L.) Pennell | 5 |  |  |  | 186.6 | 2023.6 | 42.6 | 4.4 | 3 | N/B/S |
| Lindernia micrantha D.Don | 30 |  |  |  | 416.4 | 56270.7 | 22.4 | 18.6 | 6 | W/R/S |
| Lindernia procumbens (Krock.) Borbas | 53 |  |  |  | 420.3 | 71894.2 | 19.5 | 21.6 | 5 | W/R/S |
| Linum stelleroides Planch. | 4 |  |  |  | 58.1 | 225.1 | 16.4 | 3.5 | 3 | N/B/S |
| Linum usitatissimum L. | 8 |  |  |  | 183.1 | 841.5 | 23.3 | 7.9 | 5 | N/B/L |
| Liparis japonica (Miq.) Maxim. | 12 |  |  |  | 204.8 | 12103.4 | 16.2 | 12.6 | 4 | N/B/L |
| Liparis krameri Franch. & Sav. | 19 |  |  |  | 375.3 | 54704.3 | 42.5 | 8.8 | 3 | W/R/S |
| Liparis kumokiri F.Maek. | 120 |  |  |  | 390.0 | 69146.0 | 11.1 | 35.2 | 6 | W/R/L |
| Liparis makinoana Schlech. | 8 |  |  |  | 261.9 | 23156.4 | 44.5 | 5.9 | 3 | N/B/S |
| Lipocarpha microcephala (R.Br.) Kunth | 7 |  |  |  | 233.8 | 7474.8 | 22.9 | 10.2 | 3 | N/B/L |
| Liriodendron tulipifera L. | 9 |  |  |  | 193.8 | 12996.2 | 29.0 | 6.7 | 3 | N/B/L |
| Liriope platyphylla F.T.Wang & T.Tang | 138 |  |  |  | 437.9 | 79410.4 | 9.9 | 44.1 | 7 | W/B/L |
| Liriope spicata (Thunb.) Lour. | 134 |  |  |  | 382.8 | 68546.0 | 10.7 | 35.6 | 6 | W/R/L |
| Lithospermum arvense L. | 2 |  |  |  | 100.9 | NA | 100.9 | 1.0 | 2 | N/R/S |
| Lithospermum erythrorhizon Siebold & Zucc. | 59 |  |  | LC | 390.8 | 46866.4 | 10.6 | 37.0 | 5 | W/R/L |
| Lithospermum zollingeri A.DC. | 22 |  |  |  | 381.2 | 55674.8 | 31.2 | 12.2 | 6 | W/R/S |
| Lloydia triflora (Ledeb.) Baker | 23 |  |  |  | 293.5 | 24698.1 | 26.6 | 11.0 | 5 | N/B/L |
| Lobelia chinensis Lour. | 59 |  |  |  | 377.7 | 71966.2 | 15.6 | 24.2 | 8 | W/B/S |
| Lobelia sessilifolia Lamb. | 5 |  |  |  | 348.2 | 6570.4 | 53.9 | 6.5 | 3 | W/R/S |
| Lolium multiflorum var. multiflorum Lam. | 22 |  |  |  | 417.5 | 56222.7 | 29.9 | 14.0 | 5 | W/R/S |
| Lolium multiflorum var. ramosum Guss. ex Arcang. | 3 |  |  |  | 134.0 | 179.7 | 46.2 | 2.9 | 2 | N/R/S |
| Lolium perenne L. | 48 |  |  |  | 447.1 | 80172.7 | 21.1 | 21.2 | 8 | W/B/S |
| Lonicera caerulea subsp. Edulis L. (Turcz. ex Herder) Hulten | 5 |  |  |  | 222.3 | 6387.2 | 51.5 | 4.3 | 3 | N/B/S |
| Lonicera chrysantha Turcz. | 7 |  |  |  | 247.1 | 17414.3 | 51.6 | 4.8 | 4 | N/B/S |
| Lonicera chrysantha var. crassipes Nakai | 4 |  |  |  | 151.6 | 4884.2 | 54.5 | 2.8 | 3 | N/B/S |
| Lonicera coreana Nakai | 22 |  |  |  | 411.8 | 65237.7 | 22.1 | 18.6 | 5 | W/R/S |
| Lonicera ferdinandii Franch. | 5 |  |  |  | 192.5 | 10840.5 | 58.5 | 3.3 | 3 | N/B/S |
| Lonicera harai Makino | 29 |  |  |  | 421.8 | 52776.2 | 26.2 | 16.1 | 4 | W/R/S |
| Lonicera japonica Thunb. | 344 |  |  |  | 491.4 | 107033.0 | 8.2 | 59.7 | 13 | W/B/L |
| Lonicera japonica var. repens (Siebold) Rehder | 3 |  |  |  | 82.4 | 997.6 | 43.7 | 1.9 | 2 | N/R/S |
| Lonicera maackii (Rupr.) Maxim. | 80 |  |  |  | 451.9 | 87567.0 | 15.2 | 29.7 | 8 | W/B/L |
| Lonicera maximowiczii var. maximowiczii (Rupr.) Regel | 6 |  |  |  | 201.9 | 11329.9 | 44.4 | 4.5 | 3 | N/B/S |
| Lonicera praeflorens Batalin | 134 |  |  |  | 457.0 | 81657.2 | 10.9 | 41.8 | 6 | W/R/L |
| Lonicera sachalinensis | 2 |  |  |  | 135.8 | NA | 135.8 | 1.0 | 2 | N/R/S |
| Lonicera subhispida Nakai | 10 |  |  |  | 360.1 | 26935.6 | 35.5 | 10.1 | 3 | W/R/S |
| Lonicera subsessilis Rehder | 59 |  | V |  | 306.9 | 39057.2 | 11.0 | 28.0 | 5 | N/B/L |
| Lonicera tatarinovii var. leptantha Maxim. (Rehder) Nakai | 2 |  | V |  | 101.4 | NA | 101.4 | 1.0 | 2 | N/R/S |
| Lonicera vidalii Franch. & Sav. | 5 |  |  |  | 263.9 | 1520.5 | 20.5 | 12.9 | 3 | N/B/L |
| Lophatherum gracile Brongn. | 10 |  |  |  | 372.2 | 32800.0 | 43.7 | 8.5 | 3 | W/R/S |
| Lotus corniculatus var. japonica Regel | 70 |  |  |  | 474.6 | 87007.1 | 12.2 | 39.0 | 10 | W/B/L |
| Ludwigia prostrata Roxb. | 78 |  |  |  | 419.3 | 76127.1 | 14.9 | 28.1 | 7 | W/B/S |
| Luffa cylindrica Roem. | 2 |  |  |  | 247.4 | NA | 247.4 | 1.0 | 2 | N/R/S |
| Luzula capitata (Miq.) Miq. | 295 |  |  |  | 456.4 | 97701.4 | 8.7 | 52.4 | 11 | W/B/L |
| Luzula multiflora Lej. | 14 |  |  |  | 451.4 | 65850.1 | 36.9 | 12.2 | 5 | W/R/S |
| Luzula plumosa E.Mey. | 3 |  |  |  | 33.1 | 2.3 | 11.2 | 3.0 | 2 | N/R/S |
| Luzula wahlenbergii Rupr. | 3 |  |  |  | 44.6 | 150.5 | 19.0 | 2.4 | 2 | N/R/S |
| Lychnis cognata Maxim. | 98 |  |  |  | 364.5 | 63156.1 | 9.8 | 37.1 | 5 | W/R/L |
| Lychnis fulgens Fisch. ex Spreng. | 2 |  |  |  | 148.5 | NA | 148.5 | 1.0 | 1 | N/R/S |
| Lycium chinense Mill. | 26 |  |  |  | 311.7 | 50833.8 | 25.9 | 12.0 | 6 | N/B/L |
| Lycopodium clavatum L. | 7 |  |  |  | 421.5 | 44970.4 | 79.7 | 5.3 | 3 | W/R/S |
| Lycopodium serratum Thunb. | 14 |  |  |  | 419.1 | 23108.7 | 23.6 | 17.8 | 3 | W/R/S |
| Lycopus lucidus Turcz. | 167 |  |  |  | 408.2 | 82137.6 | 10.3 | 39.8 | 9 | W/B/L |
| Lycopus maackianus (Maxim. ex Herder) Makino | 8 |  |  |  | 326.4 | 27786.5 | 56.7 | 5.8 | 5 | W/R/S |
| Lycopus ramosissimus (Makino) Makino | 6 |  |  |  | 441.5 | 40023.9 | 90.9 | 4.9 | 4 | W/R/S |
| Lycoris radiata (L'Her.) Herb. | 5 |  |  |  | 191.3 | 1869.4 | 39.2 | 4.9 | 2 | N/R/S |
| Lycoris squamigera Maxim. | 3 |  |  |  | 179.9 | 75.1 | 68.6 | 2.6 | 3 | N/B/S |
| Lygodium japonicum (Thunb.) Sw. | 22 |  |  |  | 224.7 | 17153.2 | 20.9 | 10.8 | 6 | N/B/L |
| Lysimachia barystachys Bunge | 89 |  |  |  | 405.3 | 77472.7 | 11.1 | 36.4 | 7 | W/B/L |
| Lysimachia clethroides Duby | 577 |  |  |  | 495.3 | 108245.5 | 6.4 | 77.2 | 10 | W/B/L |
| Lysimachia coreana Nakai | 16 |  | V | LC | 277.6 | 17500.0 | 22.6 | 12.3 | 4 | N/B/L |
| Lysimachia fortunei Maxim. | 3 |  |  |  | 34.2 | 41.9 | 14.5 | 2.4 | 1 | N/R/S |
| Lysimachia japonica Thunb. | 86 |  |  |  | 298.3 | 41175.7 | 9.3 | 31.9 | 9 | N/B/L |
| Lysimachia mauritiana Lam. | 7 |  |  |  | 357.3 | 23302.5 | 59.0 | 6.1 | 5 | W/R/S |
| Lysimachia vulgaris var. davurica (Ledeb.) R.Kunth | 98 |  |  |  | 385.7 | 50511.7 | 9.9 | 39.0 | 9 | W/B/L |
| Lythrum anceps (Koehne) Makino | 29 |  |  |  | 320.1 | 60133.4 | 19.7 | 16.2 | 9 | W/B/S |
| Lythrum salicaria L. | 14 |  |  |  | 446.8 | 41080.1 | 30.5 | 14.6 | 8 | W/B/S |
| Maackia amurensis var. amurensis Rupr. & Maxim. | 130 |  |  |  | 450.5 | 86396.9 | 11.2 | 40.2 | 9 | W/B/L |
| Machilus thunbergii Siebold & Zucc. | 16 |  |  |  | 172.2 | 8871.6 | 14.5 | 11.8 | 6 | N/B/L |
| Macrothelypteris oligophlebia var. elegans (Baker) Ching (Koidz.) Ching | 11 |  |  |  | 352.5 | 19739.6 | 34.5 | 10.2 | 6 | W/R/S |
| Magnolia denudata Desr. | 16 |  |  |  | 411.5 | 48323.5 | 34.6 | 11.9 | 4 | W/R/S |
| Magnolia kobus DC. | 3 |  |  | LC | 357.1 | 3626.7 | 131.0 | 2.7 | 2 | W/R/S |
| Magnolia liliflora Desr. | 3 |  |  |  | 144.9 | 370.3 | 51.9 | 2.8 | 2 | N/R/S |
| Magnolia obovata Thunb. | 12 |  |  |  | 302.8 | 32517.6 | 44.3 | 6.8 | 4 | N/B/L |
| Magnolia sieboldii K.Koch | 149 |  |  |  | 462.9 | 75710.6 | 8.3 | 55.7 | 7 | W/B/L |
| Maianthemum bifolium (L.) F.W.Schmidt | 11 |  |  |  | 283.8 | 14652.0 | 27.7 | 10.3 | 5 | N/B/L |
| Mallotus japonicus (Thunb.) Muell. Arg. | 77 |  |  |  | 287.1 | 30977.2 | 8.3 | 34.5 | 7 | N/B/L |
| Malus baccata Borkh. | 112 |  |  |  | 380.3 | 68829.5 | 11.7 | 32.5 | 8 | W/B/L |
| Malus baccata for. jackii Rehder | 2 |  |  |  | 213.4 | NA | 213.4 | 1.0 | 1 | N/R/S |
| Malus baccata var. mandshurica (Maxim.) C.K.Schneid. | 10 |  |  |  | 342.8 | 31077.2 | 27.6 | 12.4 | 5 | W/R/S |
| Malus pumila Mill. | 3 |  |  |  | 295.0 | 24.9 | 98.4 | 3.0 | 2 | N/R/S |
| Malus sieboldii (Regel) Rehder | 38 |  |  |  | 427.9 | 52642.2 | 19.9 | 21.5 | 6 | W/R/S |
| Malva verticillata L. | 3 |  |  |  | 127.9 | 3962.4 | 84.6 | 1.5 | 3 | N/B/S |
| Marsilea quadrifolia L. | 3 |  |  |  | 207.4 | 2855.8 | 90.1 | 2.3 | 2 | N/R/S |
| Matricaria chamomilla L. | 2 |  |  |  | 3.0 | NA | 3.0 | 1.0 | 2 | N/R/S |
| Matricaria matricariodes Porter | 2 |  |  |  | 73.5 | NA | 73.5 | 1.0 | 1 | N/R/S |
| Matteuccia orientalis (Hook.) Trevis. | 117 |  |  |  | 355.1 | 60315.5 | 9.4 | 37.8 | 8 | W/B/L |
| Matteuccia struthiopteris (L.) Tod. | 20 |  |  |  | 261.9 | 23372.4 | 18.8 | 14.0 | 5 | N/B/L |
| Mazus miquelii Makino | 28 |  |  |  | 396.3 | 57384.8 | 25.4 | 15.6 | 5 | W/R/S |
| Mazus pumilus (Burm.f.) Steenis | 251 |  |  |  | 441.5 | 97963.9 | 9.8 | 45.2 | 12 | W/B/L |
| Mazus stachydifolius (Turcz.) Maxim. | 4 |  |  |  | 220.3 | 16660.0 | 99.1 | 2.2 | 3 | N/B/S |
| Medicago lupulina L. | 14 |  |  |  | 348.7 | 51154.8 | 46.3 | 7.5 | 4 | W/R/S |
| Medicago polymorpha L. | 11 |  |  |  | 407.7 | 40665.5 | 49.3 | 8.3 | 6 | W/R/S |
| Medicago ruthenica | 3 |  |  |  | 27.5 | 43.6 | 12.1 | 2.3 | 1 | N/R/S |
| Medicago sativa L. | 24 |  |  |  | 350.3 | 60287.7 | 30.1 | 11.6 | 8 | W/B/S |
| Meehania urticifolia (Miq.) Makino | 214 |  |  |  | 409.9 | 87103.1 | 9.0 | 45.4 | 9 | W/B/L |
| Meehania urticifolia for. leucantha Hara | 2 |  |  |  | 227.2 | NA | 227.2 | 1.0 | 2 | N/R/S |
| Melampyrum roseum Maxim. | 338 |  |  |  | 490.6 | 100022.6 | 7.9 | 62.3 | 8 | W/B/L |
| Melampyrum roseum var. japonicum Franch. & Sav. | 25 |  |  |  | 359.7 | 60344.1 | 26.0 | 13.9 | 4 | W/R/S |
| Melampyrum roseum var. ovalifolium Nakai ex Beauverd | 96 |  |  |  | 436.4 | 85844.5 | 13.4 | 32.4 | 9 | W/B/L |
| Melampyrum setaceum (Maxim.) Nakai | 36 |  |  |  | 345.2 | 49271.1 | 11.3 | 30.6 | 6 | W/R/L |
| Melampyrum setaceum var. nakaianum (Tuyama) T.Yamaz. | 27 |  |  |  | 380.6 | 34500.5 | 16.0 | 23.8 | 5 | W/R/S |
| Melia azedarach L. | 23 |  |  |  | 168.0 | 9148.9 | 9.3 | 18.1 | 5 | N/B/L |
| Melica nutans L. | 22 |  |  |  | 335.0 | 35935.4 | 16.9 | 19.9 | 4 | W/R/S |
| Melica onoei Franch. & Sav. | 31 |  |  |  | 361.9 | 67855.6 | 27.1 | 13.4 | 6 | W/R/S |
| Melilotus alba Medicus | 27 |  |  |  | 222.6 | 25764.9 | 20.8 | 10.7 | 7 | N/B/L |
| Melilotus suaveolens Ledeb. | 31 |  |  |  | 420.2 | 62361.6 | 26.8 | 15.7 | 6 | W/R/S |
| Meliosma myriantha Siebold & Zucc. | 45 |  |  |  | 309.0 | 53516.6 | 12.5 | 24.7 | 4 | N/B/L |
| Meliosma oldhamii Maxim. | 46 |  |  |  | 268.5 | 32761.7 | 9.9 | 27.0 | 4 | N/B/L |
| Melothria japonica Maxim. | 9 |  |  | LC | 196.1 | 6008.9 | 22.0 | 8.9 | 5 | N/B/L |
| Menispermum dauricum DC. | 155 |  |  |  | 400.6 | 74013.6 | 11.6 | 34.5 | 9 | W/B/L |
| Mentha arvensis L. | 31 |  |  |  | 409.9 | 71635.8 | 33.3 | 12.3 | 6 | W/R/S |
| Menyanthes trifoliata L. | 3 | V |  | VU | 37.0 | 8.0 | 13.9 | 2.7 | 2 | N/R/S |
| Metaplexis japonica (Thunb.) Makino | 278 |  |  |  | 461.9 | 98184.8 | 9.1 | 51.0 | 12 | W/B/L |
| Metasequoia glyptostroboides Hu & Cheng | 7 |  |  |  | 306.1 | 23836.2 | 68.7 | 4.5 | 4 | N/B/S |
| Meterostachys sikokianus (Makino) Nakai | 11 |  |  |  | 321.0 | 29404.0 | 44.0 | 7.3 | 3 | W/R/S |
| Microstegium japonicum (Miq.) Koidz. | 11 |  |  |  | 218.6 | 20915.3 | 34.5 | 6.3 | 5 | N/B/L |
| Microstegium vimineum var. imberbe (Nees ex Steud.) Honda | 25 |  |  |  | 334.6 | 45075.0 | 18.2 | 18.4 | 7 | W/B/S |
| Microstegium vimineum var. vimineum (Trin.) A.Camus | 66 |  |  |  | 423.1 | 66893.4 | 13.3 | 31.9 | 7 | W/B/L |
| Milium effusum L. | 31 |  |  |  | 377.3 | 37643.7 | 23.8 | 15.9 | 4 | W/R/S |
| Mimulus nepalensis Benth. | 21 |  |  |  | 291.3 | 40337.6 | 21.8 | 13.4 | 5 | N/B/L |
| Mirabilis jalapa L. | 5 |  |  |  | 350.7 | 24465.2 | 68.6 | 5.1 | 3 | W/R/S |
| Miscanthus changii Y.N.Lee | 2 |  | V |  | 359.4 | NA | 359.4 | 1.0 | 2 | W/R/S |
| Miscanthus sacchariflorus (Maxim.) Benth. | 30 |  |  |  | 463.2 | 65560.0 | 20.2 | 23.0 | 7 | W/B/S |
| Miscanthus sinensis for. gracillimus (Hitchc.) Ohwi | 2 |  |  |  | 212.9 | NA | 212.9 | 1.0 | 2 | N/R/S |
| Miscanthus sinensis var. ionandros (Nakai) Y.N.Lee | 2 |  |  |  | 23.6 | NA | 23.6 | 1.0 | 2 | N/R/S |
| Miscanthus sinensis var. purpurascens (Andersson) Rendle | 83 |  |  |  | 422.5 | 81922.4 | 13.4 | 31.5 | 9 | W/B/L |
| Miscanthus sinensis var. sinensis Andersson | 319 |  |  |  | 472.7 | 101173.0 | 8.5 | 55.8 | 11 | W/B/L |
| Mitchella undulata Siebold & Zucc. | 2 |  |  |  | 40.4 | NA | 40.4 | 1.0 | 2 | N/R/S |
| Mitrasacme alsinoides R.Br. | 3 |  |  |  | 225.8 | 4636.7 | 100.8 | 2.2 | 2 | N/R/S |
| Mitrasacme pygmaea R.Br. | 23 |  |  |  | 329.2 | 42438.0 | 26.9 | 12.3 | 6 | W/R/S |
| Moehringia lateriflora (L.) Fenzl | 4 |  |  |  | 127.9 | 2607.1 | 25.7 | 5.0 | 2 | N/R/S |
| Moliniopsis japonica (Hack.) Hayata | 11 |  |  |  | 456.9 | 58822.3 | 44.6 | 10.2 | 5 | W/R/S |
| Mollugo pentaphylla L. | 91 |  |  |  | 421.3 | 71100.4 | 12.3 | 34.2 | 9 | W/B/L |
| Mollugo verticillata L. | 2 |  |  |  | 138.1 | NA | 138.1 | 1.0 | 1 | N/R/S |
| Monochoria korsakowii Regel & Maack | 11 |  |  |  | 227.7 | 10091.0 | 14.9 | 15.3 | 4 | N/B/L |
| Monochoria vaginalis var. plantaginea (Roxb.) Solms | 44 |  |  |  | 410.5 | 72363.6 | 20.1 | 20.4 | 8 | W/B/S |
| Monotropa hypopithys L. | 4 |  |  | LC | 260.5 | 18815.9 | 110.1 | 2.4 | 2 | N/R/S |
| Monotropa uniflora L. | 10 |  |  | LC | 336.4 | 39669.4 | 61.0 | 5.5 | 3 | W/R/S |
| Morus alba L. | 155 |  |  |  | 437.2 | 95353.6 | 10.9 | 40.2 | 8 | W/B/L |
| Morus bombucis for. dissecta Nakai | 17 |  |  |  | 309.2 | 29508.5 | 14.6 | 21.2 | 4 | N/B/L |
| Morus bombycis var. bombycis Koidz. | 351 |  |  |  | 488.0 | 98988.5 | 8.4 | 58.4 | 10 | W/B/L |
| Morus mongolica (Bureau) C.K.Schneid. | 2 |  |  |  | 55.7 | NA | 55.7 | 1.0 | 2 | N/R/S |
| Morus tiliaefolia Makino | 9 |  |  |  | 274.4 | 17342.2 | 41.9 | 6.5 | 4 | N/B/L |
| Mosla chinensis Maxim. | 9 |  |  |  | 310.8 | 30454.5 | 50.1 | 6.2 | 4 | N/B/L |
| Mosla dianthera (Buch.-Ham. ex Roxb.) ex Maxim. | 142 |  |  |  | 464.4 | 91949.6 | 12.4 | 37.4 | 10 | W/B/L |
| Mosla japonica (Benth.) Maxim. | 26 |  |  |  | 433.8 | 71019.7 | 27.7 | 15.7 | 7 | W/B/S |
| Mosla punctulata (J.F.Gmelin) Nakai | 281 |  |  |  | 462.3 | 100248.0 | 9.8 | 47.0 | 10 | W/B/L |
| Muhlenbergia hakonensis (Hack.) Makino | 8 |  |  |  | 238.5 | 20234.2 | 23.4 | 10.2 | 3 | N/B/L |
| Muhlenbergia huegelii Trin. | 25 |  |  |  | 362.0 | 49262.9 | 29.9 | 12.1 | 4 | W/R/S |
| Muhlenbergia japonica Steud. | 69 |  |  |  | 456.7 | 81533.8 | 19.3 | 23.7 | 7 | W/B/S |
| Mukdenia rosii (Oliv.) Koidz. | 57 |  |  |  | 363.5 | 43586.8 | 12.3 | 29.7 | 8 | W/B/L |
| Myriophyllum spicatum L. | 5 |  |  |  | 235.3 | 19149.4 | 81.8 | 2.9 | 4 | N/B/S |
| Myriophyllum verticillatum L. | 9 |  |  |  | 404.5 | 52330.8 | 68.9 | 5.9 | 8 | W/B/S |
| Najas marina L. | 2 |  |  |  | 175.9 | NA | 175.9 | 1.0 | 2 | N/R/S |
| Nandina domestica Thunb. | 5 |  |  |  | 215.8 | 7093.6 | 60.2 | 3.6 | 2 | N/R/S |
| Nanocnide japonica Blume | 14 |  |  |  | 380.3 | 17547.1 | 32.4 | 11.7 | 5 | W/R/S |
| Narcissus tazetta var. chinensis Roem. | 4 |  |  |  | 160.9 | 1292.1 | 47.9 | 3.4 | 3 | N/B/S |
| Neillia ueki Nakai | 13 |  |  | LC | 190.2 | 15618.3 | 11.7 | 16.3 | 7 | N/B/L |
| Nelumbo nucifera Gaertn. | 3 |  |  |  | 261.7 | 5308.6 | 130.8 | 2.0 | 2 | N/R/S |
| Neolitsea sericea (Blume) Koidz. | 14 |  |  |  | 157.5 | 6192.3 | 15.4 | 10.2 | 4 | N/B/L |
| Nepeta cataria L. | 11 |  |  |  | 413.6 | 53045.6 | 45.8 | 9.0 | 4 | W/R/S |
| Nephrolepis cordifolia Presl | 2 |  |  |  | 165.5 | NA | 165.5 | 1.0 | 2 | N/R/S |
| Nymphoides indica (L.) Kuntze | 2 |  |  |  | 120.2 | NA | 120.2 | 1.0 | 2 | N/R/S |
| Nymphoides peltata (J.G.Gmelin) Kuntze | 4 |  |  |  | 190.5 | 787.6 | 14.7 | 12.9 | 4 | N/B/L |
| Oenanthe javanica (Blume) DC. | 67 |  |  |  | 377.0 | 78901.7 | 16.1 | 23.4 | 12 | W/B/S |
| Oenothera biennis L. | 433 |  |  |  | 468.9 | 100063.1 | 7.8 | 59.8 | 11 | W/B/L |
| Oenothera erythrosepala Borbas | 20 |  |  |  | 432.2 | 76328.6 | 28.1 | 15.4 | 7 | W/B/S |
| Oenothera stricta Ledeb. | 2 |  |  |  | 11.7 | NA | 11.7 | 1.0 | 1 | N/R/S |
| Onoclea sensibilis L. | 63 |  |  |  | 333.3 | 59838.1 | 14.5 | 23.0 | 8 | W/B/S |
| Onychium japonicum (Thunb.) Kunze | 3 |  |  |  | 383.9 | 0.1 | 128.0 | 3.0 | 2 | W/R/S |
| Ophiopogon jaburan (Kunth) Lodd. | 4 |  |  |  | 92.1 | 1716.1 | 34.4 | 2.7 | 3 | N/B/S |
| Ophiopogon japonicus (L.f.) KerGawl. | 49 |  |  |  | 299.3 | 38014.6 | 8.5 | 35.2 | 6 | N/B/L |
| Oplismenus undulatifolius var. japonicus (Steud.) Koidz. | 7 |  |  |  | 257.9 | 24924.9 | 9.1 | 28.2 | 4 | N/B/L |
| Oplismenus undulatifolius var. undulatifolius (Ard.) P.Beauv. | 539 |  |  |  | 461.0 | 103679.0 | 6.6 | 70.0 | 12 | W/B/L |
| Oreorchis patens (Lindl.) Lindl. | 31 |  |  |  | 340.4 | 46797.5 | 22.3 | 15.3 | 4 | W/R/S |
| Orixa japonica Thunb. | 31 |  |  |  | 249.5 | 20914.3 | 9.8 | 25.6 | 4 | N/B/L |
| Orostachys filirera (Nakai) Nakai | 2 |  | V |  | 104.5 | NA | 104.5 | 1.0 | 2 | N/R/S |
| Orostachys japonica (Maxim.) A.Berger | 30 |  |  |  | 374.8 | 72274.2 | 27.3 | 13.7 | 6 | W/R/S |
| Orostachys malacophylla (Pall.) Fisch. | 5 |  |  |  | 463.8 | 43792.8 | 115.5 | 4.0 | 4 | W/R/S |
| Oryza sativa var. sativa L. | 6 |  |  |  | 236.9 | 19288.6 | 44.8 | 5.3 | 4 | N/B/S |
| Osmanthus heterophyllus (G.Don) P.S.Green | 5 |  |  |  | 229.6 | 4942.4 | 52.1 | 4.4 | 3 | N/B/S |
| Osmorhiza aristata (Thunb.) Makino & Yabe | 49 |  |  |  | 430.6 | 69232.3 | 21.3 | 20.2 | 5 | W/R/S |
| Osmunda cinnamomea L. | 53 |  |  |  | 341.6 | 58008.4 | 13.6 | 25.0 | 6 | W/R/S |
| Osmunda claytoniana L. | 3 |  |  |  | 263.5 | 11034.2 | 143.3 | 1.8 | 3 | N/B/S |
| Osmunda japonica Thunb. | 311 |  |  |  | 463.8 | 96462.2 | 7.9 | 58.9 | 9 | W/B/L |
| Ottelia alismoides (L.) Pers. | 5 |  |  |  | 203.6 | 13023.3 | 59.6 | 3.4 | 2 | N/R/S |
| Oxalis acetosella var. acetosella L. | 13 |  |  |  | 281.3 | 35758.8 | 30.9 | 9.1 | 4 | N/B/L |
| Oxalis articulata Sabigny | 4 |  |  |  | 155.0 | 4176.5 | 42.2 | 3.7 | 3 | N/B/S |
| Oxalis corniculata L. | 350 |  |  |  | 482.2 | 104985.3 | 8.4 | 57.2 | 11 | W/B/L |
| Oxalis obtriangulata Maxim. | 102 |  |  |  | 416.9 | 71570.9 | 11.8 | 35.4 | 6 | W/R/L |
| Oxalis stricta L. | 85 |  |  |  | 433.1 | 73147.0 | 13.2 | 32.7 | 7 | W/B/L |
| Paederia scandens var. angustifolia (Nakai) T.B.Lee | 4 |  |  |  | 20.9 | 126.9 | 9.5 | 2.2 | 3 | N/B/S |
| Paederia scandens var. scandens (Lour.) Merr. | 211 |  |  |  | 449.9 | 91107.4 | 7.8 | 57.5 | 9 | W/B/L |
| Paeonia japonica (Makino) Miyabe & Takeda | 16 |  |  | NT | 304.2 | 33332.3 | 26.1 | 11.7 | 4 | N/B/L |
| Paeonia japonica var. pillosa Nakai | 3 |  |  |  | 58.6 | 1.6 | 19.6 | 3.0 | 2 | N/R/S |
| Paeonia lactiflora Pall. | 4 |  |  |  | 45.9 | 144.3 | 7.6 | 6.0 | 2 | N/R/S |
| Paeonia lactiflora var. trichocarpa (Bunge) Stern | 5 |  |  |  | 53.0 | 312.7 | 5.1 | 10.4 | 2 | N/R/L |
| Paeonia obovata Maxim. | 3 | V |  | CR | 120.3 | 516.4 | 47.0 | 2.6 | 3 | N/B/S |
| Panax ginseng C.A.Mey. | 2 |  |  |  | 37.5 | NA | 37.5 | 1.0 | 2 | N/R/S |
| Panicum bisulcatum Thunb. | 120 |  |  |  | 426.7 | 87187.9 | 13.8 | 31.0 | 8 | W/B/L |
| Panicum dichotomiflorum Michx. | 43 |  |  |  | 397.6 | 67515.1 | 19.7 | 20.2 | 7 | W/B/S |
| Panicum miliaceum L. | 3 |  |  |  | 85.8 | 407.2 | 37.0 | 2.3 | 2 | N/R/S |
| Panicum virgatum L. | 2 |  |  |  | 19.3 | NA | 19.3 | 1.0 | 2 | N/R/S |
| Parasenecio adenostyloides (Franch. & Sav. ex Maxim.) H.Koyama | 4 |  |  | LC | 69.6 | 411.0 | 18.6 | 3.7 | 2 | N/R/S |
| Parasenecio auriculata (DC.) H.Koyama | 7 |  |  |  | 122.8 | 874.2 | 19.2 | 6.4 | 2 | N/R/L |
| Parasenecio auriculata var. matsumurana Nakai | 18 |  |  |  | 356.7 | 41220.7 | 22.4 | 15.9 | 3 | W/R/S |
| Parasenecio auriculatus var. kamtschaticus (DC.) H. Koyama (Maxim.) H. Koyama | 18 |  |  |  | 241.9 | 19450.8 | 24.9 | 9.7 | 4 | N/B/L |
| Parasenecio firmus (Kom.) Y.L.Chen | 9 |  |  |  | 381.7 | 38346.6 | 35.5 | 10.8 | 3 | W/R/S |
| Parasenecio hastatus subsp. Orientalis (L.) H. Koyama (Kitam.) H. Koyama | 3 |  |  |  | 30.5 | 7.6 | 11.8 | 2.6 | 2 | N/R/S |
| Parasenecio pseudotamingasa (Nakai) B. U. Oh | 3 |  | V | LC | 107.8 | 5.0 | 36.0 | 3.0 | 1 | N/R/S |
| Paris verticillata M.Bieb. | 124 |  |  |  | 420.7 | 74978.5 | 11.8 | 35.5 | 5 | W/R/L |
| Parnassia palustris L. | 33 |  |  |  | 454.2 | 54466.6 | 20.0 | 22.7 | 6 | W/R/S |
| Parthenocissus tricuspidata (Siebold & Zucc.) Planch. | 331 |  |  |  | 463.3 | 102212.5 | 8.3 | 55.8 | 11 | W/B/L |
| Paspalum distichum L. | 2 |  |  |  | 230.4 | NA | 230.4 | 1.0 | 2 | N/R/S |
| Paspalum distichum var. indutum Shinners | 5 |  |  |  | 148.1 | 1339.8 | 33.0 | 4.5 | 3 | N/B/S |
| Paspalum thunbergii Kunth ex Steud. | 118 |  |  |  | 456.9 | 87317.2 | 13.1 | 35.0 | 9 | W/B/L |
| Patrinia rupestris (Pall.) Juss. | 22 |  |  |  | 258.4 | 24193.4 | 19.7 | 13.1 | 6 | N/B/L |
| Patrinia saniculaefolia Hemsl. | 31 |  |  |  | 406.9 | 36731.4 | 11.2 | 36.4 | 3 | W/R/L |
| Patrinia scabiosaefolia Fisch. ex Trevir. | 430 |  |  |  | 495.4 | 104505.7 | 7.3 | 67.8 | 10 | W/B/L |
| Patrinia villosa (Thunb.) Juss. | 451 |  |  |  | 469.4 | 100227.4 | 6.9 | 67.8 | 10 | W/B/L |
| Paulownia coreana Uyeki | 148 |  | V |  | 437.6 | 94242.5 | 11.2 | 39.1 | 12 | W/B/L |
| Paulownia tomentosa (Thunb.) Steud. | 49 |  |  |  | 425.6 | 74591.2 | 16.4 | 26.0 | 6 | W/R/S |
| Pedicularis resupinata for. albiflora (Nakai) W.T.Lee | 2 |  |  |  | 40.9 | NA | 40.9 | 1.0 | 2 | N/R/S |
| Pedicularis resupinata L. | 69 |  |  |  | 400.2 | 52484.9 | 11.7 | 34.1 | 6 | W/R/L |
| Pedicularis resupinata var. gigantea Nakai | 4 |  |  |  | 19.5 | 114.0 | 9.8 | 2.0 | 3 | N/B/S |
| Pedicularis resupinata var. oppositifolia Miq. | 6 |  |  |  | 368.8 | 33562.0 | 79.0 | 4.7 | 4 | W/R/S |
| Pennisetum alopecuroides var. alopecuroides (L.) Spreng. | 282 |  |  |  | 480.9 | 103087.4 | 9.3 | 51.9 | 10 | W/B/L |
| Pennisetum alopecuroides var. viridescens (Miq.) Ohwi | 2 |  |  |  | 141.3 | NA | 141.3 | 1.0 | 2 | N/R/S |
| Penthorum chinense Pursh | 3 |  |  | LC | 246.3 | 6606.2 | 116.4 | 2.1 | 3 | N/B/S |
| Perilla frutescens var. acuta Kudo | 4 |  |  |  | 206.2 | 5586.5 | 63.0 | 3.3 | 3 | N/B/S |
| Perilla frutescens var. japonica (Hassk.) Hara | 14 |  |  |  | 367.8 | 53849.1 | 43.0 | 8.6 | 5 | W/R/S |
| Perilla frutescens var. purpurascens (Hayata) H.W.Li | 9 |  |  |  | 366.0 | 47128.1 | 38.9 | 9.4 | 4 | W/R/S |
| Persicaria breviochreata (Makino) Ohwi | 10 |  |  |  | 221.0 | 21895.3 | 41.7 | 5.3 | 5 | N/B/S |
| Persicaria conspicua (Nakai) Nakai ex Mori | 34 |  |  |  | 310.4 | 52783.7 | 17.7 | 17.5 | 9 | N/B/L |
| Persicaria debilis (Meisn.) H.Gross ex Mori | 8 |  |  |  | 320.7 | 28512.7 | 44.5 | 7.2 | 4 | W/R/S |
| Persicaria dissitiflora (Hemsl.) H.Gross ex Mori | 129 |  |  |  | 377.4 | 70627.5 | 9.1 | 41.5 | 7 | W/B/L |
| Persicaria filiformis (Thunb.) Nakai ex Mori | 294 |  |  |  | 459.6 | 96300.5 | 8.5 | 54.3 | 8 | W/B/L |
| Persicaria hastatosagittata (Makino) Nakai ex Mori | 3 |  |  |  | 265.0 | 1458.2 | 95.2 | 2.8 | 2 | N/R/S |
| Persicaria hydropiper var. hydropiper (L.) Spach | 172 |  |  |  | 471.7 | 96724.7 | 10.4 | 45.2 | 9 | W/B/L |
| Persicaria japonica (Meisn.) H.Gross ex Nakai | 83 |  |  |  | 423.7 | 78050.6 | 14.3 | 29.6 | 9 | W/B/L |
| Persicaria lapathifolia for. alba Y.N.Lee | 7 |  |  |  | 283.5 | 21193.5 | 31.7 | 8.9 | 4 | N/B/L |
| Persicaria lapathifolia var. lapathifolia (L.) Gray | 172 |  |  |  | 431.8 | 89527.3 | 11.6 | 37.3 | 9 | W/B/L |
| Persicaria longiseta (Bruijn) Kitag. | 367 |  |  |  | 479.9 | 103271.2 | 8.2 | 58.2 | 10 | W/B/L |
| Persicaria maackiana (Regel) Nakai ex Mori | 4 |  |  |  | 101.9 | 714.8 | 30.1 | 3.4 | 1 | N/R/S |
| Persicaria modosa Opiz | 112 |  |  |  | 419.9 | 82999.4 | 12.1 | 34.7 | 7 | W/B/L |
| Persicaria muricata (Meisn.) Nemoto | 24 |  |  |  | 372.5 | 57391.4 | 23.3 | 16.0 | 5 | W/R/S |
| Persicaria nepalensis (Meisn.) H.Gross | 144 |  |  |  | 426.1 | 76605.0 | 10.2 | 41.9 | 9 | W/B/L |
| Persicaria nodosa (Pers.) Opiz | 5 |  |  |  | 417.2 | 26270.3 | 104.3 | 4.0 | 3 | W/R/S |
| Persicaria orientalis (L.) Spach | 26 |  |  |  | 345.3 | 54283.1 | 23.5 | 14.7 | 5 | W/R/S |
| Persicaria perfoliata (L.) H.Gross | 240 |  |  |  | 451.1 | 91496.7 | 10.0 | 45.2 | 12 | W/B/L |
| Persicaria posumbu var. laxiflora (Meisn.) H.Hara | 169 |  |  |  | 443.7 | 89319.7 | 10.6 | 41.7 | 8 | W/B/L |
| Persicaria praetermissa (Hook.f.) Hara | 2 |  |  |  | 211.0 | NA | 211.0 | 1.0 | 2 | N/R/S |
| Persicaria pubescens (Blume) H.Hara | 46 |  |  |  | 388.7 | 71607.2 | 21.2 | 18.3 | 6 | W/R/S |
| Persicaria sagittata (L.) H.Gross ex Nakai | 254 |  |  |  | 481.5 | 93887.6 | 9.9 | 48.7 | 10 | W/B/L |
| Persicaria senticosa var. senticosa (Meisn.) H.Gross ex Nakai | 417 |  |  |  | 495.5 | 103871.9 | 7.4 | 67.3 | 12 | W/B/L |
| Persicaria thunbergii (Siebold & Zucc.) H.Gross ex Nakai | 432 |  |  |  | 478.8 | 103847.2 | 7.3 | 65.2 | 11 | W/B/L |
| Persicaria tinctoria H.Gross | 5 |  |  |  | 340.9 | 36422.8 | 121.0 | 2.8 | 4 | W/R/S |
| Persicaria trigonocarpa (Makino) Nakai | 4 |  |  |  | 339.8 | 11013.1 | 102.4 | 3.3 | 3 | W/R/S |
| Persicaria viscofera var. robusta (Makino) Hiyama | 13 |  |  |  | 308.8 | 28104.5 | 23.1 | 13.4 | 4 | N/B/L |
| Persicaria viscofera var. viscofera (Makino) Nakai | 53 |  |  |  | 362.5 | 67544.1 | 14.6 | 24.8 | 7 | W/B/S |
| Persicaria viscosa (Hamilt. ex D.Don) H.Gross ex Nakai | 16 |  |  |  | 391.1 | 41652.8 | 29.0 | 13.5 | 5 | W/R/S |
| Persicaria vulgaris Webb & Moq. | 43 |  |  |  | 406.4 | 76652.0 | 16.9 | 24.1 | 6 | W/R/S |
| Petasites japonicum (Siebold & Zucc.) Maxim. | 165 |  |  |  | 428.8 | 92004.1 | 11.1 | 38.8 | 8 | W/B/L |
| Peucedanum hakuunense Nakai | 3 |  |  |  | 327.4 | 341.2 | 115.4 | 2.8 | 2 | W/R/S |
| Peucedanum insolens Kitag. | 7 |  | V |  | 63.3 | 1055.9 | 10.5 | 6.0 | 4 | N/B/S |
| Peucedanum japonicum Thunb. | 3 |  |  |  | 294.7 | 1816.6 | 127.2 | 2.3 | 3 | N/B/S |
| Peucedanum terebinthaceum (Fisch.) Fisch. ex DC. | 356 |  |  |  | 486.2 | 101697.7 | 8.2 | 59.2 | 11 | W/B/L |
| Phacellanthus tubiflorus Siebold & Zucc. | 2 |  |  |  | 200.8 | NA | 200.8 | 1.0 | 1 | N/R/S |
| Phaenosperma globosa Munro ex Benth. | 5 |  |  |  | 398.8 | 12734.5 | 74.1 | 5.4 | 3 | W/R/S |
| Phalaris arundinacea L. | 39 |  |  |  | 385.7 | 67367.8 | 22.3 | 17.3 | 8 | W/B/S |
| Pharbitis nil (L.) Choisy | 44 |  |  |  | 355.3 | 60718.9 | 20.4 | 17.4 | 8 | W/B/S |
| Phegopteirs koreana B. Y. Sun & C. H. Kim | 2 |  |  |  | 31.3 | NA | 31.3 | 1.0 | 1 | N/R/S |
| Phegopteris connectilis (Michx.) Watt | 3 |  |  |  | 243.0 | 5168.3 | 116.8 | 2.1 | 2 | N/R/S |
| Phegopteris decursivepinnata (H.C.Hall) Fee | 34 |  |  |  | 415.7 | 62856.3 | 21.3 | 19.5 | 4 | W/R/S |
| Phellodendron amurense Rupr. | 28 |  |  |  | 267.9 | 31228.2 | 14.7 | 18.2 | 3 | N/B/L |
| Philadelphus pekinensis Rupr. | 22 |  |  |  | 375.6 | 38173.1 | 20.3 | 18.5 | 6 | W/R/S |
| Philadelphus schrenckii var. jackii Koehne | 19 |  |  |  | 312.8 | 47657.2 | 36.4 | 8.6 | 6 | N/B/L |
| Philadelphus schrenkii var. lasiogynus (Nakai) W.T.Lee | 7 |  |  |  | 277.5 | 10003.4 | 43.7 | 6.3 | 3 | N/B/L |
| Philadelphus schrenkii var. schrenkii Rupr. | 217 |  | V |  | 475.3 | 87705.3 | 9.5 | 50.1 | 10 | W/B/L |
| Philadelphus seoulensis Y.H.Chung & H.C.Shin | 3 |  | V |  | 125.3 | 533.7 | 46.8 | 2.7 | 2 | N/R/S |
| Philadelphus tenuifolius Rupr. & Maxim. | 45 |  |  |  | 391.5 | 72785.3 | 18.4 | 21.3 | 7 | W/B/S |
| Phleum pratense L. | 28 |  |  |  | 366.0 | 65834.2 | 28.6 | 12.8 | 3 | W/R/S |
| Phlomis umbrosa Turcz. | 85 |  |  |  | 400.8 | 77362.5 | 13.9 | 28.9 | 4 | W/R/L |
| Phlox drummondii Hook. | 2 |  |  |  | 0.3 | NA | 0.3 | 1.0 | 1 | N/R/S |
| Phlox subulata L. | 21 |  |  |  | 407.6 | 40463.6 | 21.2 | 19.2 | 4 | W/R/S |
| Phragmites communis Trin. | 84 |  |  |  | 473.4 | 84752.9 | 13.3 | 35.6 | 10 | W/B/L |
| Phragmites japonica Steud. | 183 |  |  |  | 472.3 | 87732.0 | 10.3 | 45.9 | 10 | W/B/L |
| Phryma leptostachya var. asiatica H.Hara | 475 |  |  |  | 488.9 | 106227.5 | 7.0 | 70.3 | 13 | W/B/L |
| Phtheirospermum japonicum (Thunb.) Kanitz | 226 |  |  |  | 445.0 | 93474.3 | 10.1 | 43.9 | 10 | W/B/L |
| Phyllanthus urinaria L. | 16 |  |  |  | 312.3 | 42315.4 | 32.3 | 9.7 | 7 | N/B/L |
| Phyllanthus ussuriensis Rupr. & Maxim. | 86 |  |  |  | 425.9 | 80733.1 | 14.6 | 29.2 | 8 | W/B/L |
| Phyllostachys bambusoides Siebold & Zucc. | 17 |  |  |  | 218.7 | 9861.7 | 15.0 | 14.6 | 5 | N/B/L |
| Phyllostachys nigro var. henonis (Bean) Stapf ex Rendle | 31 |  |  |  | 433.4 | 60770.7 | 18.1 | 23.9 | 5 | W/R/S |
| Physaliastrum japonicum (Franch. & Sav.) Honda | 32 |  |  |  | 329.5 | 48545.1 | 18.8 | 17.5 | 4 | W/R/S |
| Physalis alkekengi var. francheti (Mast.) Hort | 25 |  |  |  | 331.2 | 51206.1 | 28.6 | 11.6 | 8 | W/B/S |
| Physalis angulata L. | 4 |  |  |  | 173.0 | 8153.6 | 63.8 | 2.7 | 3 | N/B/S |
| Phytolacca acinosa Roxb. | 22 |  |  |  | 440.9 | 57353.8 | 31.1 | 14.2 | 8 | W/B/S |
| Phytolacca americana L. | 328 |  |  |  | 438.6 | 97934.6 | 7.5 | 58.2 | 10 | W/B/L |
| Picea abies (L.) H.Karst. | 2 |  |  |  | 197.1 | NA | 197.1 | 1.0 | 1 | N/R/S |
| Picea jezoensis (Siebold & Zucc.) Carriere | 3 |  |  | VU | 61.2 | 8.5 | 20.6 | 3.0 | 2 | N/R/S |
| Picrasma quassioides (D.Don) Benn. | 165 |  |  |  | 457.8 | 89997.9 | 10.5 | 43.4 | 9 | W/B/L |
| Picris hieracioides var. koreana Kitam. | 132 |  |  |  | 398.3 | 75144.3 | 10.9 | 36.5 | 8 | W/B/L |
| Pilea hamaoi Makino | 55 |  |  |  | 372.4 | 69025.1 | 15.1 | 24.7 | 8 | W/B/S |
| Pilea japonica (Maxim.) Hand.-Mazz. | 52 |  |  |  | 373.2 | 63601.3 | 15.8 | 23.6 | 6 | W/R/S |
| Pilea mongolica Wedd. | 173 |  |  |  | 399.9 | 80563.2 | 10.9 | 36.6 | 8 | W/B/L |
| Pilea peploides (Gaudich.) Hook. & Arn. | 27 |  |  |  | 414.7 | 64287.0 | 26.2 | 15.8 | 5 | W/R/S |
| Pimpinella brachycarpa (Kom.) Nakai | 163 |  |  |  | 481.3 | 90620.1 | 10.7 | 45.1 | 8 | W/B/L |
| Pimpinella gustavohegiana Koidz. | 9 |  |  |  | 349.1 | 51059.2 | 61.6 | 5.7 | 4 | W/R/S |
| Pimpinella koreana (Yabe) Nakai | 9 |  |  |  | 361.6 | 38576.0 | 54.3 | 6.7 | 6 | W/R/S |
| Pinellia ternata (Thunb.) Breitenb. | 69 |  |  |  | 443.2 | 89732.1 | 19.8 | 22.4 | 7 | W/B/S |
| Pinellia tripartita (Blume) Schott | 3 |  |  |  | 176.9 | 299.3 | 61.1 | 2.9 | 2 | N/R/S |
| Pinus banksiana | 3 |  |  |  | 48.7 | 62.5 | 21.5 | 2.3 | 2 | N/R/S |
| Pinus densiflora Siebold & Zucc. | 501 |  |  |  | 451.0 | 97382.8 | 6.6 | 68.8 | 11 | W/B/L |
| Pinus koraiensis Siebold & Zucc. | 151 |  |  |  | 432.6 | 85508.0 | 11.2 | 38.8 | 7 | W/B/L |
| Pinus parviflora Siebold & Zucc. | 3 |  |  |  | 41.0 | 1.0 | 13.7 | 3.0 | 2 | N/R/S |
| Pinus rigida Mill. | 141 |  |  |  | 425.5 | 74633.8 | 11.5 | 37.0 | 9 | W/B/L |
| Pinus strobus L. | 5 |  |  |  | 361.6 | 30861.7 | 121.9 | 3.0 | 2 | W/R/S |
| Pinus sylvestris L. | 4 |  |  |  | 158.8 | 1069.6 | 46.4 | 3.4 | 3 | N/B/S |
| Pinus taeda L. | 2 |  |  |  | 18.3 | NA | 18.3 | 1.0 | 2 | N/R/S |
| Pinus thunbergii Parl. | 60 |  |  |  | 437.7 | 89787.9 | 15.0 | 29.3 | 9 | W/B/L |
| Pittosporum tobira (Thunb.) W.T.Aiton | 2 |  |  |  | 39.7 | NA | 39.7 | 1.0 | 1 | N/R/S |
| Plantago asiatica L. | 492 |  |  |  | 486.8 | 106141.6 | 7.4 | 65.9 | 11 | W/B/L |
| Plantago camtschatica Cham. ex Link | 5 |  |  |  | 213.5 | 9204.5 | 54.8 | 3.9 | 5 | N/B/S |
| Plantago depressa Willd. | 20 |  |  |  | 376.0 | 46249.7 | 28.3 | 13.3 | 7 | W/B/S |
| Plantago major for. yezomaritima (Koidz.) Ohwi | 2 |  |  |  | 2.1 | NA | 2.1 | 1.0 | 1 | N/R/S |
| Plantago major var. japonica (Franch. & Sav.) Miyabe | 10 |  |  |  | 450.0 | 40765.1 | 38.3 | 11.8 | 4 | W/R/S |
| Plantago sibirica Poir. | 8 |  |  |  | 191.3 | 2726.4 | 24.9 | 7.7 | 6 | N/B/L |
| Plantago virginica L. | 14 |  |  |  | 326.2 | 52101.8 | 45.1 | 7.2 | 7 | W/B/S |
| Platanthera freynii Kraenzl. | 15 |  |  |  | 390.8 | 67956.2 | 50.0 | 7.8 | 5 | W/R/S |
| Platanthera hologlottis Maxim. | 2 |  |  | LC | 21.0 | NA | 21.0 | 1.0 | 1 | N/R/S |
| Platanthera mandarinorum var. brachycentron (Franch. & Sav.) Koidz. ex Ohwi | 12 |  |  |  | 380.1 | 46430.0 | 42.2 | 9.0 | 5 | W/R/S |
| Platanthera sachalinensis F.Schmidt | 3 |  |  |  | 200.0 | 2020.4 | 79.7 | 2.5 | 2 | N/R/S |
| Platanthera ussuriensis (Regel & Maack) Maxim. | 3 |  |  |  | 169.7 | 5174.5 | 99.8 | 1.7 | 2 | N/R/S |
| Platanus occidentalis L. | 7 |  |  |  | 326.5 | 14662.2 | 22.6 | 14.4 | 6 | W/R/S |
| Platanus orientalis L. | 2 |  |  |  | 238.0 | NA | 238.0 | 1.0 | 1 | N/R/S |
| Platanus Xhispanica Munchh. | 3 |  |  |  | 183.6 | 0.8 | 61.2 | 3.0 | 3 | N/B/S |
| Platycarya strobilacea for. coreana (Miq.) W.T.Lee | 3 |  | V |  | 152.6 | 896.1 | 57.9 | 2.6 | 2 | N/R/S |
| Platycarya strobilacea var. strobilacea for. strobilacea Siebold & Zucc. | 295 |  |  |  | 379.9 | 79806.8 | 6.8 | 55.8 | 10 | W/B/L |
| Platycladus orientalis (L.) Franco | 40 |  |  | LC | 437.8 | 57569.9 | 20.4 | 21.5 | 7 | W/B/S |
| Platycodon grandiflorum (Jacq.) A.DC. | 180 |  |  |  | 476.2 | 99627.9 | 10.9 | 43.7 | 8 | W/B/L |
| Platycodon grandiflorum for. albiflorum (Honda) H.Hara | 12 |  |  |  | 209.0 | 12092.7 | 26.1 | 8.0 | 6 | N/B/L |
| Plectranthus japonicus (Burm.) Koidz. | 211 |  |  |  | 444.9 | 88322.2 | 9.3 | 47.6 | 9 | W/B/L |
| Plectranthus serra Maxim. | 27 |  |  |  | 346.6 | 47303.9 | 22.6 | 15.3 | 6 | W/R/S |
| Pleurosoriopsis makinoi (Maxim. ex Makino) Fomin | 2 |  |  |  | 262.3 | NA | 262.3 | 1.0 | 1 | N/R/S |
| Pleurospermum camtschaticum Hoffm. | 4 |  |  |  | 216.3 | 10849.6 | 87.3 | 2.5 | 2 | N/R/S |
| Poa acroleuca Steud. | 39 |  |  |  | 340.8 | 65567.9 | 23.4 | 14.6 | 6 | W/R/S |
| Poa annua L. | 73 |  |  |  | 390.9 | 78193.4 | 16.1 | 24.2 | 8 | W/B/S |
| Poa compressa L. | 11 |  |  |  | 409.5 | 46473.0 | 44.8 | 9.2 | 4 | W/R/S |
| Poa glauca Vahl | 2 |  |  |  | 21.2 | NA | 21.2 | 1.0 | 1 | N/R/S |
| Poa hisauchii Honda | 13 |  |  |  | 276.3 | 29603.6 | 21.1 | 13.1 | 4 | N/B/L |
| Poa matsumurae Hack. | 5 |  |  |  | 227.0 | 18191.4 | 67.6 | 3.4 | 3 | N/B/S |
| Poa nemoralis L. | 6 |  |  |  | 237.4 | 16610.5 | 40.4 | 5.9 | 4 | N/B/S |
| Poa nipponica Koidz. | 3 |  |  |  | 250.6 | 3085.5 | 103.8 | 2.4 | 2 | N/R/S |
| Poa pratensis L. | 33 |  |  |  | 339.4 | 60081.7 | 22.8 | 14.9 | 8 | W/B/S |
| Poa sphondylodes Trin. | 141 |  |  |  | 444.8 | 89973.6 | 13.5 | 32.9 | 9 | W/B/L |
| Poa trivialis L. | 4 |  |  |  | 221.9 | 12087.1 | 84.7 | 2.6 | 3 | N/B/S |
| Poa viridula Palib. | 17 |  |  |  | 426.8 | 49682.9 | 29.8 | 14.3 | 5 | W/R/S |
| Pogonia japonica Rchb.f. | 2 |  |  | NT | 169.3 | NA | 169.3 | 1.0 | 2 | N/R/S |
| Pogonia minor (Makino) Makino | 2 |  |  | NT | 43.3 | NA | 43.3 | 1.0 | 1 | N/R/S |
| Polygala japonica Houtt. | 127 |  |  |  | 459.4 | 92358.3 | 10.3 | 44.5 | 8 | W/B/L |
| Polygala sibirica L. | 4 |  |  |  | 49.3 | 112.6 | 15.6 | 3.2 | 3 | N/B/S |
| Polygala tatarinowii Regel | 5 |  |  |  | 105.8 | 3145.8 | 31.0 | 3.4 | 2 | N/R/S |
| Polygala tenuifolia Willd. | 2 |  |  | NT | 10.2 | NA | 10.2 | 1.0 | 2 | N/R/S |
| Polygonatum cryptanthum H.Lev. & Vaniot | 8 |  |  |  | 191.3 | 4442.9 | 32.7 | 5.8 | 4 | N/B/S |
| Polygonatum desoulavyi Kom. | 2 |  |  |  | 152.4 | NA | 152.4 | 1.0 | 2 | N/R/S |
| Polygonatum falcatum A.Gray | 22 |  |  |  | 317.3 | 55737.1 | 22.7 | 14.0 | 4 | W/R/S |
| Polygonatum humile Fisch. ex Maxim. | 62 |  |  |  | 442.0 | 84899.0 | 18.4 | 24.0 | 7 | W/B/S |
| Polygonatum inflatum Kom. | 119 |  |  |  | 436.5 | 80502.1 | 11.2 | 38.9 | 9 | W/B/L |
| Polygonatum infundiflorum Y.S.Kim, B.U.Oh, C.G.Jang | 3 |  | V |  | 31.1 | 129.4 | 16.3 | 1.9 | 2 | N/R/S |
| Polygonatum involucratum (Franch. & Sav.) Maxim. | 219 |  |  |  | 450.9 | 94100.7 | 8.9 | 50.8 | 11 | W/B/L |
| Polygonatum lasianthum Maxim. | 171 |  |  |  | 445.7 | 85336.0 | 9.9 | 45.0 | 7 | W/B/L |
| Polygonatum odoratum var. pluriflorum (Miq.) Ohwi | 504 |  |  |  | 475.2 | 102934.8 | 6.0 | 79.4 | 11 | W/B/L |
| Polygonatum stenophyllum Maxim. | 3 |  |  | NT | 342.0 | 8555.2 | 159.4 | 2.1 | 1 | W/R/S |
| Polygonatum thunbergii Morr. & Decne. | 5 |  |  |  | 125.0 | 3335.1 | 22.3 | 5.6 | 3 | N/B/S |
| Polygonum aviculare L. | 171 |  |  |  | 442.6 | 99183.9 | 12.1 | 36.7 | 9 | W/B/L |
| Polygonum manshuriense (Petrov ex Kom.) Kom. | 35 |  |  |  | 451.4 | 80726.5 | 21.5 | 21.0 | 5 | W/R/S |
| Polypogon fugax Nees ex Steud. | 6 |  |  |  | 323.1 | 20475.3 | 64.5 | 5.0 | 4 | W/R/S |
| Polystichum braunii (Spenn.) Fee | 20 |  |  |  | 243.0 | 18620.1 | 19.6 | 12.4 | 4 | N/B/L |
| Polystichum craspedosorum (Maxim.) Diels | 19 |  |  |  | 279.9 | 24429.7 | 17.3 | 16.2 | 6 | N/B/L |
| Polystichum ovato-paleaceum var. coraiense (Kodama) Sa. Kurata (H. Christ) Sa. Kurata | 12 |  |  |  | 259.6 | 25381.3 | 40.2 | 6.5 | 4 | N/B/L |
| Polystichum ovatopaleaceum var. ovatopaleaceum (Kodama) Sa.Kurata | 2 |  |  |  | 26.9 | NA | 26.9 | 1.0 | 2 | N/R/S |
| Polystichum polyblepharum var. polyblepharum (Roem. ex Kunze) C.Presl | 12 |  |  |  | 348.7 | 27302.9 | 31.7 | 11.0 | 4 | W/R/S |
| Polystichum retrosopaleaceum (Kodama) Tagawa | 2 |  |  |  | 0.0 | NA | 0.0 | 1.0 | 1 | N/R/S |
| Polystichum tripteron for. tripteron (Kunze) C.Presl | 222 |  |  |  | 457.0 | 88368.3 | 9.4 | 48.8 | 8 | W/B/L |
| Poncirus trifoliatus (L.) Raf. | 39 |  |  |  | 395.3 | 67225.7 | 23.7 | 16.7 | 7 | W/B/S |
| Populus alba L. | 15 |  |  |  | 416.3 | 56751.3 | 48.2 | 8.6 | 5 | W/R/S |
| Populus davidiana Dode | 16 |  |  |  | 457.3 | 63051.8 | 47.1 | 9.7 | 4 | W/R/S |
| Populus deltoides Marsh. | 6 |  |  |  | 268.8 | 25439.1 | 72.2 | 3.7 | 4 | N/B/S |
| Populus euramericana Guinier | 4 |  |  |  | 246.6 | 2337.1 | 41.1 | 6.0 | 3 | N/B/S |
| Populus maximowiczii A.Henry | 2 |  |  |  | 16.6 | NA | 16.6 | 1.0 | 1 | N/R/S |
| Populus nigra var. italica Koehne | 6 |  |  |  | 227.0 | 16620.2 | 76.4 | 3.0 | 2 | N/R/S |
| Populus tomentiglandulosa T.B.Lee | 58 |  | V |  | 443.9 | 82574.6 | 16.2 | 27.5 | 7 | W/B/S |
| Portulaca grandiflora Hook. | 5 |  |  |  | 231.4 | 18860.8 | 47.7 | 4.8 | 3 | N/B/S |
| Portulaca oleracea L. | 133 |  |  |  | 441.5 | 90410.4 | 13.0 | 34.0 | 10 | W/B/L |
| Potamogeton crispus L. | 12 |  |  |  | 430.0 | 67940.3 | 54.0 | 8.0 | 7 | W/B/S |
| Potamogeton cristatus Regel & Maack | 4 |  |  |  | 236.1 | 597.0 | 61.7 | 3.8 | 2 | N/R/S |
| Potamogeton distincuts A.Benn. | 13 |  |  |  | 387.4 | 48915.0 | 39.6 | 9.8 | 6 | W/R/S |
| Potamogeton malaianus Miq. | 2 |  |  |  | 0.7 | NA | 0.7 | 1.0 | 2 | N/R/S |
| Potamogeton octandrus var. octandrus Poir. | 2 |  |  |  | 343.0 | NA | 343.0 | 1.0 | 2 | W/R/S |
| Potamogeton oxyphyllus | 2 |  |  |  | 13.3 | NA | 13.3 | 1.0 | 1 | N/R/S |
| Potamogeton pusillus L. | 4 |  |  |  | 188.2 | 2607.3 | 53.3 | 3.5 | 3 | N/B/S |
| Potentilla amurensis Maxim. | 2 |  |  |  | 114.9 | NA | 114.9 | 1.0 | 1 | N/R/S |
| Potentilla centigrana Maxim. | 26 |  |  |  | 349.5 | 52808.6 | 25.3 | 13.8 | 6 | W/R/S |
| Potentilla chinensis var. chinensis Ser. | 160 |  |  |  | 473.9 | 85764.7 | 9.9 | 47.7 | 8 | W/B/L |
| Potentilla cryptotaeniae Maxim. | 96 |  |  |  | 316.0 | 41243.6 | 9.0 | 35.3 | 7 | W/B/L |
| Potentilla dickinsii var. dickinsii Franch. & Sav. | 97 |  |  |  | 469.3 | 84015.2 | 12.9 | 36.4 | 4 | W/R/L |
| Potentilla discolor Bunge | 46 |  |  | LC | 383.3 | 39745.7 | 16.4 | 23.3 | 7 | W/B/S |
| Potentilla fragarioides var. major Maxim. | 527 |  |  |  | 457.5 | 101030.7 | 6.5 | 70.5 | 11 | W/B/L |
| Potentilla freyniana Bornm. | 262 |  |  |  | 466.8 | 93161.5 | 9.3 | 50.3 | 9 | W/B/L |
| Potentilla freyniana var. villosa Nakai | 3 |  |  |  | 88.7 | 479.6 | 36.1 | 2.5 | 2 | N/R/S |
| Potentilla fruticosa var. rigida (Wall.) Th.Wolf | 3 |  |  |  | 11.0 | 32.1 | 7.7 | 1.4 | 3 | N/B/S |
| Potentilla kleiniana Wight & Arn. | 106 |  |  |  | 425.8 | 77001.1 | 12.6 | 33.7 | 8 | W/B/L |
| Potentilla supina L. | 31 |  |  |  | 382.0 | 54765.6 | 21.0 | 18.2 | 7 | W/B/S |
| Potentilla yokusaina Makino | 33 |  |  |  | 383.0 | 55258.2 | 26.4 | 14.5 | 4 | W/R/S |
| Pourthiaea villosa var. villosa (Thunb.) Decne. | 61 |  |  |  | 390.0 | 50965.1 | 13.7 | 28.5 | 5 | W/R/L |
| Prenanthes ochroleuca (Maxim.) Hemsl. | 12 |  |  |  | 432.2 | 60442.7 | 37.9 | 11.4 | 3 | W/R/S |
| Prenanthes tatarinowii Maxim. | 2 |  |  |  | 83.8 | NA | 83.8 | 1.0 | 1 | N/R/S |
| Primula jesoana Miq. | 23 |  |  |  | 321.9 | 28815.8 | 17.1 | 18.9 | 3 | W/R/S |
| Primula sieboldii E.Morren | 29 |  |  |  | 391.0 | 50401.7 | 26.5 | 14.8 | 5 | W/R/S |
| Prunella vulgaris var. lilacina Nakai | 427 |  |  |  | 486.8 | 104568.6 | 7.6 | 63.7 | 13 | W/B/L |
| Prunus armeniaca var. ansu Maxim. | 21 |  |  |  | 399.9 | 56605.6 | 31.2 | 12.8 | 4 | W/R/S |
| Prunus avium L. | 4 |  |  |  | 309.3 | 24987.3 | 150.4 | 2.1 | 2 | N/R/S |
| Prunus choreiana Nakai ex Handb. | 3 |  | V |  | 227.5 | 3871.8 | 105.9 | 2.1 | 2 | N/R/S |
| Prunus davidiana (Carriere) Franch. | 25 |  |  |  | 343.3 | 58136.7 | 27.7 | 12.4 | 7 | W/B/S |
| Prunus glandulosa for. albiplena Koehne | 2 |  |  |  | 135.9 | NA | 135.9 | 1.0 | 2 | N/R/S |
| Prunus glandulosa for. glandulosa Thunb. | 16 |  |  |  | 387.8 | 49340.5 | 35.6 | 10.9 | 5 | W/R/S |
| Prunus ishidoyana Nakai | 2 |  | V |  | 208.8 | NA | 208.8 | 1.0 | 1 | N/R/S |
| Prunus japonica var. nakaii (H.Lev.) Rehder | 117 |  |  |  | 439.2 | 84559.8 | 10.2 | 43.0 | 8 | W/B/L |
| Prunus maackii Rupr. | 11 |  |  |  | 221.9 | 13518.9 | 11.9 | 18.7 | 3 | N/B/L |
| Prunus mandshurica var. glabra (Maxim.) Koehne Nakai | 15 |  |  |  | 319.7 | 42954.7 | 34.5 | 9.3 | 4 | W/R/S |
| Prunus maximowiczii Rupr. | 9 |  |  |  | 347.9 | 24271.5 | 41.1 | 8.5 | 3 | W/R/S |
| Prunus mume for. alba (Carr.) Rehder | 2 |  |  |  | 125.6 | NA | 125.6 | 1.0 | 1 | N/R/S |
| Prunus mume for. mume Siebold & Zucc. | 27 |  |  |  | 411.5 | 63816.6 | 29.3 | 14.0 | 5 | W/R/S |
| Prunus padus for. glauca (Nakai) Kitag. | 3 |  |  |  | 40.6 | 36.2 | 14.6 | 2.8 | 2 | N/R/S |
| Prunus padus for. padus L. | 110 |  |  |  | 401.0 | 64979.8 | 10.0 | 40.2 | 10 | W/B/L |
| Prunus persica for. persica (L.) Batsch | 221 |  |  |  | 495.2 | 97296.3 | 9.6 | 51.4 | 9 | W/B/L |
| Prunus salicina var. salicina Lindl. | 13 |  |  |  | 326.0 | 28841.7 | 24.0 | 13.6 | 6 | W/R/S |
| Prunus sargentii Rehder | 235 |  |  |  | 448.9 | 100428.1 | 9.0 | 49.9 | 8 | W/B/L |
| Prunus serrulata var. pubescens (Makino) Nakai | 76 |  |  |  | 374.0 | 75248.4 | 12.8 | 29.1 | 7 | W/B/L |
| Prunus serrulata var. sontagiae Nakai | 23 |  |  |  | 354.6 | 48410.1 | 26.4 | 13.4 | 3 | W/R/S |
| Prunus serrulata var. spontanea (Maxim.) E.H.Wilson | 170 |  |  |  | 442.7 | 91763.1 | 10.8 | 40.8 | 8 | W/B/L |
| Prunus serrulata var. tomentella Nakai | 6 |  |  |  | 188.0 | 13756.6 | 44.1 | 4.3 | 4 | N/B/S |
| Prunus spachiana for. Ascendens (Lavallee ex Ed. Otto) Kitam. (Makino) Kitam. | 19 |  |  |  | 372.2 | 61331.6 | 37.8 | 9.9 | 4 | W/R/S |
| Prunus tomentosa Thunb. | 23 |  |  |  | 370.1 | 54874.0 | 25.9 | 14.3 | 4 | W/R/S |
| Prunus verecunda var. verecunda (Koidz.) Koehne | 74 |  |  |  | 444.8 | 85018.9 | 17.8 | 24.9 | 6 | W/R/S |
| Prunus yedoensis Matsum. | 41 |  |  | EN | 384.8 | 63357.9 | 18.8 | 20.5 | 8 | W/B/S |
| Pseudolysimachion dahuricum (Steven) Holub | 15 |  |  |  | 205.0 | 10682.1 | 17.4 | 11.8 | 4 | N/B/L |
| Pseudolysimachion kiusianum (Furumi) Holub | 3 |  |  |  | 155.5 | 1202.0 | 70.1 | 2.2 | 2 | N/R/S |
| Pseudolysimachion pyrethrinum (Nakai) T.Yamaz. | 6 |  |  |  | 226.5 | 10499.6 | 56.1 | 4.0 | 4 | N/B/S |
| Pseudostellaria coreana (Nakai) Ohwi | 42 |  |  |  | 404.7 | 77210.1 | 19.4 | 20.9 | 6 | W/R/S |
| Pseudostellaria davidii (Franch.) Pax ex Pax & Hoffm. | 36 |  |  |  | 365.4 | 47193.7 | 15.9 | 23.0 | 4 | W/R/S |
| Pseudostellaria heterophylla (Miq.) Pax ex Pax & Hoffm. | 289 |  |  |  | 451.3 | 92047.0 | 7.9 | 56.8 | 13 | W/B/L |
| Pseudostellaria japonica Pax | 4 |  |  |  | 140.4 | 2811.6 | 54.6 | 2.6 | 3 | N/B/S |
| Pseudostellaria multiflora Y.N.Lee | 2 |  |  |  | 68.5 | NA | 68.5 | 1.0 | 2 | N/R/S |
| Pseudostellaria palibiniana (Takeda) Ohwi | 202 |  |  |  | 436.7 | 91147.0 | 10.1 | 43.2 | 11 | W/B/L |
| Pseudostellaria setulosa Ohwi | 6 |  | V |  | 219.1 | 16737.0 | 67.0 | 3.3 | 4 | N/B/S |
| Pteridium aquilinum var. latiusculum (Desv.) Underw. ex Hell. | 308 |  |  |  | 436.0 | 97027.8 | 8.0 | 54.4 | 10 | W/B/L |
| Pteris multifida Poir. | 19 |  |  |  | 238.2 | 15151.0 | 14.7 | 16.2 | 6 | N/B/L |
| Pterygocalyx volubilis Maxim. | 2 |  |  | VU | 31.1 | NA | 31.1 | 1.0 | 2 | N/R/S |
| Pueraria lobata (Willd.) Ohwi | 513 |  |  |  | 489.1 | 107304.0 | 6.7 | 72.9 | 12 | W/B/L |
| Pulsatilla koreana (Yabe ex Nakai) Nakai ex Mori | 195 |  |  |  | 446.8 | 89982.2 | 11.2 | 40.0 | 10 | W/B/L |
| Pyracantha angustifolia (Franch.) C.K.Schneid. | 5 |  |  |  | 256.4 | 14075.1 | 67.0 | 3.8 | 3 | N/B/S |
| Pyrola denticulata Koidz. | 2 |  |  |  | 132.2 | NA | 132.2 | 1.0 | 2 | N/R/S |
| Pyrola japonica Klenze ex Alef. | 533 |  |  |  | 486.8 | 102810.9 | 5.8 | 83.9 | 12 | W/B/L |
| Pyrola japonica var. subaphylla (Maxim.) H.Andres | 16 |  |  |  | 253.2 | 18158.9 | 20.6 | 12.3 | 5 | N/B/L |
| Pyrrosia linearifolia (Hook.) Ching | 29 |  |  |  | 359.7 | 41753.4 | 20.1 | 17.9 | 6 | W/R/S |
| Pyrrosia petiolosa (H.Christ & Baroni) Ching | 14 |  |  |  | 181.5 | 12433.4 | 20.3 | 8.9 | 4 | N/B/L |
| Pyrus calleryana var. fauriei (C.K.Schneid.) Rehder | 47 |  |  |  | 351.9 | 39454.9 | 13.3 | 26.5 | 7 | W/B/S |
| Pyrus pyrifolia (Burm.f.) Nakai | 37 |  |  |  | 422.2 | 63997.9 | 18.6 | 22.7 | 4 | W/R/S |
| Pyrus pyrifolia var. culta (Makino) Nakai | 4 |  |  |  | 258.9 | 20268.6 | 121.1 | 2.1 | 2 | N/R/S |
| Pyrus ussuriensis var. ussuriensis Maxim. | 51 |  |  |  | 467.9 | 77572.4 | 22.2 | 21.1 | 6 | W/R/S |
| Quamoclit coccinea Moench | 38 |  |  |  | 360.6 | 57101.2 | 25.2 | 14.3 | 7 | W/B/S |
| Quamoclit pennata (Desr.) Bojer | 3 |  |  |  | 376.6 | 3242.0 | 176.0 | 2.1 | 2 | W/R/S |
| Quercus acuta for. acuta Thunb. ex Murray | 5 |  |  |  | 1.1 | 0.5 | 0.4 | 2.7 | 1 | N/R/S |
| Quercus acutissima Carruth. | 258 |  |  |  | 441.6 | 96663.6 | 8.9 | 49.4 | 9 | W/B/L |
| Quercus aliena Blume | 452 |  |  |  | 434.1 | 96005.8 | 6.1 | 71.2 | 14 | W/B/L |
| Quercus aliena var. pellucida Blume | 5 |  |  |  | 334.5 | 30893.4 | 80.7 | 4.1 | 2 | W/R/S |
| Quercus dentata Thunb. ex Murray | 362 |  |  |  | 489.1 | 104486.9 | 7.7 | 63.2 | 12 | W/B/L |
| Quercus glauca Thunb. ex Murray | 2 |  |  |  | 323.1 | NA | 323.1 | 1.0 | 2 | W/R/S |
| Quercus mongolica Fisch. ex Ledeb. | 481 |  |  |  | 474.4 | 100585.0 | 6.4 | 74.7 | 12 | W/B/L |
| Quercus mongolica var. crispula (Blume) H.Ohashi | 9 |  |  |  | 298.6 | 17416.4 | 38.1 | 7.8 | 4 | N/B/L |
| Quercus phillyraeoides A.Gray | 2 |  |  |  | 110.8 | NA | 110.8 | 1.0 | 1 | N/R/S |
| Quercus salicina Blume | 3 |  |  |  | 9.0 | 24.2 | 6.9 | 1.3 | 3 | N/B/S |
| Quercus serrata Thunb. ex Murray | 578 |  |  |  | 491.4 | 106395.9 | 5.8 | 84.8 | 12 | W/B/L |
| Quercus variabilis Blume | 347 |  |  |  | 426.4 | 94426.1 | 7.9 | 53.9 | 10 | W/B/L |
| Quercus xdentatomongolica Nakai | 18 |  |  |  | 391.7 | 63077.1 | 23.7 | 16.5 | 5 | W/R/S |
| Quercus xdentatoserratoides T.B.Lee | 2 |  |  |  | 305.6 | NA | 305.6 | 1.0 | 2 | N/R/S |
| Quercus xmccormickii Carruth. | 7 |  |  |  | 195.4 | 13463.0 | 42.7 | 4.6 | 3 | N/B/S |
| Quercus xmccormickoserrata T.B.Lee | 3 |  |  |  | 43.8 | 8.4 | 14.8 | 3.0 | 2 | N/R/S |
| Quercus xurticaefolia Blume | 11 |  |  |  | 339.8 | 46034.6 | 49.4 | 6.9 | 5 | W/R/S |
| Ranunculus cantoniensis DC. | 23 |  |  |  | 394.1 | 55626.7 | 32.6 | 12.1 | 5 | W/R/S |
| Ranunculus chinensis Bunge | 141 |  |  |  | 418.4 | 88770.7 | 12.4 | 33.8 | 10 | W/B/L |
| Ranunculus extorris Hance | 3 |  |  |  | 392.9 | 1567.2 | 187.7 | 2.1 | 3 | W/R/S |
| Ranunculus japonicus Thunb. | 210 |  |  |  | 473.1 | 95262.8 | 10.3 | 46.0 | 9 | W/B/L |
| Ranunculus quelpaertensis (H.Lev.) Nakai | 18 |  |  |  | 439.9 | 58425.3 | 28.3 | 15.6 | 7 | W/B/S |
| Ranunculus sceleratus L. | 49 |  |  |  | 406.4 | 76934.4 | 20.5 | 19.8 | 7 | W/B/S |
| Ranunculus silerifolius Lev. | 2 |  |  |  | 327.9 | NA | 327.9 | 1.0 | 2 | W/R/S |
| Ranunculus tachiroei Franch. & Sav. | 63 |  |  |  | 488.0 | 85099.8 | 17.9 | 27.2 | 9 | W/B/S |
| Raphanus sativus L. | 5 |  |  |  | 312.2 | 23208.4 | 84.0 | 3.7 | 4 | N/B/S |
| Raphanus sativus var. hortensis for. raphanistroides Makino | 6 |  |  |  | 295.3 | 20287.3 | 28.6 | 10.3 | 3 | N/B/L |
| Rhamnella frangulioides (Maxim.) Weberb. | 39 |  |  |  | 303.3 | 27241.6 | 13.8 | 21.9 | 7 | N/B/L |
| Rhamnus crenata Siebold & Zucc. | 3 |  |  |  | 140.4 | 691.2 | 53.8 | 2.6 | 2 | N/R/S |
| Rhamnus davurica Pall. | 66 |  |  |  | 359.6 | 64074.7 | 14.8 | 24.4 | 8 | W/B/S |
| Rhamnus koraiensis C.K.Schneid. | 6 |  |  |  | 399.8 | 32368.1 | 73.8 | 5.4 | 4 | W/R/S |
| Rhamnus parvifolia Bunge | 8 |  |  |  | 138.5 | 3291.9 | 14.1 | 9.8 | 5 | N/B/L |
| Rhamnus ussuriensis J.Vass | 6 |  |  |  | 134.8 | 485.8 | 2.0 | 65.8 | 3 | N/B/L |
| Rhamnus yoshinoi Makino | 128 |  |  |  | 424.9 | 82006.3 | 9.9 | 43.0 | 7 | W/B/L |
| Rhaponticum uniflorum (L.) DC. | 46 |  |  |  | 409.0 | 45017.7 | 15.4 | 26.5 | 5 | W/R/S |
| Rhododendron micranthum Turcz. | 20 |  |  |  | 118.2 | 3192.0 | 5.7 | 20.8 | 4 | N/B/L |
| Rhododendron mucronulatum var. ciliatum Nakai | 65 |  |  |  | 441.6 | 83845.9 | 15.6 | 28.3 | 6 | W/R/S |
| Rhododendron mucronulatum var. mucronulatum Turcz. | 592 |  | V |  | 467.7 | 105659.4 | 6.2 | 75.1 | 11 | W/B/L |
| Rhododendron schlippenbachii for. albiflorum (Uyeki) T.B.Lee | 3 |  | V |  | 418.3 | 20875.9 | 212.9 | 2.0 | 3 | W/R/S |
| Rhododendron schlippenbachii Maxim. | 425 |  |  |  | 464.2 | 94676.4 | 6.7 | 69.7 | 9 | W/B/L |
| Rhododendron tschonoskii var. tschonoskii Maxim. | 3 |  |  | LC | 37.8 | 24.1 | 16.7 | 2.3 | 2 | N/R/S |
| Rhododendron yedoense for. poukhanense (H.Lev.) Sugim. | 201 |  | V |  | 437.7 | 90640.3 | 8.8 | 50.0 | 8 | W/B/L |
| Rhodotypos scandens (Thunb.) Makino | 9 |  |  |  | 259.0 | 17495.0 | 26.0 | 9.9 | 2 | N/R/L |
| Rhus javanica L. | 543 |  |  |  | 491.4 | 107081.8 | 6.7 | 73.7 | 12 | W/B/L |
| Rhus sylvestris Siebold & Zucc. | 78 |  |  |  | 420.1 | 77859.1 | 10.5 | 39.9 | 8 | W/B/L |
| Rhus tricocarpa Miq. | 454 |  |  |  | 476.5 | 102034.5 | 7.2 | 66.5 | 10 | W/B/L |
| Rhus verniciflua Stokes | 49 |  |  |  | 419.1 | 64168.3 | 15.3 | 27.3 | 8 | W/B/S |
| Rhynchosia acuminatifolia MAKINO | 15 |  |  |  | 284.4 | 40237.7 | 34.3 | 8.3 | 4 | N/B/L |
| Rhynchosia volubilis Lour. | 35 |  |  |  | 426.4 | 76976.0 | 23.6 | 18.0 | 5 | W/R/S |
| Rhynchospora chinensis Nees & Mey. ex Nees | 3 |  |  |  | 314.8 | 17585.2 | 191.9 | 1.6 | 1 | N/R/S |
| Rhynchospora faberi C.B.Clarke | 3 |  |  |  | 267.0 | 12581.1 | 154.6 | 1.7 | 3 | N/B/S |
| Rhynchospora fauriei Franch. | 2 |  |  |  | 265.4 | NA | 265.4 | 1.0 | 2 | N/R/S |
| Ribes fasciculatum var. chinense Maxim. | 90 |  |  |  | 402.9 | 78899.6 | 12.6 | 31.9 | 9 | W/B/L |
| Ribes mandshuricum for. mandshuricum (Maxim.) Kom. | 23 |  |  |  | 362.6 | 30856.4 | 22.2 | 16.3 | 5 | W/R/S |
| Ribes mandshuricum for. subglabrum (Kom.) Kitag. | 5 |  |  |  | 40.3 | 323.0 | 11.3 | 3.6 | 2 | N/R/S |
| Ribes maximowiczianum Kom. | 8 |  |  |  | 223.6 | 5413.9 | 24.6 | 9.1 | 3 | N/B/L |
| Ricinus communis L. | 12 |  |  |  | 334.2 | 28545.5 | 25.0 | 13.4 | 4 | W/R/S |
| Robinia hispida L. | 3 |  |  |  | 350.8 | 22737.1 | 203.1 | 1.7 | 2 | W/R/S |
| Robinia pseudo-acacia L. | 320 |  |  |  | 488.9 | 103704.5 | 9.1 | 53.5 | 10 | W/B/L |
| Rodgersia podophylla A.Gray | 58 |  |  | LC | 323.2 | 37161.0 | 12.8 | 25.3 | 5 | W/R/S |
| Rohdea japonica (Thunb.) Roth | 2 |  |  |  | 0.1 | NA | 0.1 | 1.0 | 1 | N/R/S |
| Rorippa cantoniensis (Lour.) Ohwi | 22 |  |  |  | 361.0 | 50599.8 | 38.2 | 9.4 | 6 | W/R/S |
| Rorippa globosa (Turcz.) Hayek | 4 |  |  |  | 189.2 | 11519.7 | 77.3 | 2.4 | 3 | N/B/S |
| Rorippa indica (L.) Hiern | 211 |  |  |  | 437.0 | 91651.1 | 10.1 | 43.2 | 13 | W/B/L |
| Rorippa palustris (Leyss.) Besser | 156 |  |  |  | 418.8 | 86008.2 | 10.7 | 39.1 | 13 | W/B/L |
| Rosa acicularis Lindl. | 2 |  |  |  | 11.9 | NA | 11.9 | 1.0 | 1 | N/R/S |
| Rosa banksiae Aiton | 5 |  |  |  | 236.1 | 19123.8 | 81.0 | 2.9 | 3 | N/B/S |
| Rosa davurica Pall. | 6 |  |  |  | 39.7 | 451.6 | 10.3 | 3.8 | 2 | N/R/S |
| Rosa koreana Kom. | 2 |  |  | LC | 0.6 | NA | 0.6 | 1.0 | 2 | N/R/S |
| Rosa maximowicziana var. maximowicziana Regel | 37 |  |  |  | 291.9 | 33050.8 | 15.8 | 18.5 | 7 | N/B/L |
| Rosa multiflora var. adenochaeta (Koidz.) Ohwi | 2 |  |  |  | 191.3 | NA | 191.3 | 1.0 | 1 | N/R/S |
| Rosa multiflora var. multiflora Thunb. | 566 |  |  |  | 488.0 | 103617.5 | 6.0 | 80.8 | 11 | W/B/L |
| Rosa pimpinellifolia L. | 3 |  |  |  | 34.1 | 47.2 | 14.3 | 2.4 | 2 | N/R/S |
| Rosa rugosa var. rugosa Thunb. | 15 |  |  |  | 396.0 | 52307.1 | 42.4 | 9.3 | 7 | W/B/S |
| Rosa suavis Willd. | 2 |  |  |  | 167.9 | NA | 167.9 | 1.0 | 1 | N/R/S |
| Rosa taisensis Nakai | 7 |  |  |  | 67.4 | 1155.2 | 11.5 | 5.9 | 3 | N/B/S |
| Rosa wichuraiana Crep. ex Franch. & Sav. | 42 |  |  |  | 380.9 | 51464.0 | 14.0 | 27.1 | 8 | W/B/S |
| Rosa wichuraiana for. ellipsoidea Nakai | 11 |  |  |  | 176.6 | 15559.0 | 17.6 | 10.0 | 4 | N/B/L |
| Rosa wichuraiana for. rosiflora Nakai | 4 |  |  |  | 137.0 | 2519.8 | 45.5 | 3.0 | 2 | N/R/S |
| Rotala indica var. uliginosa (Miq.) Koehne | 3 |  |  |  | 183.9 | 580.0 | 65.3 | 2.8 | 2 | N/R/S |
| Rotala mexicana Ohwi | 5 |  |  |  | 295.6 | 28518.7 | 82.5 | 3.6 | 4 | N/B/S |
| Rubia akane Nakai | 361 |  |  |  | 454.6 | 98255.8 | 7.1 | 63.7 | 12 | W/B/L |
| Rubia chinensis var. chinensis Regel & Maack | 82 |  |  |  | 398.7 | 68755.0 | 13.4 | 29.9 | 7 | W/B/L |
| Rubia chinensis var. glabrescens (Nakai) Kitag. | 3 |  |  |  | 299.4 | 294.8 | 101.0 | 3.0 | 1 | N/R/S |
| Rubia cordifolia var. pratensis Maxim. | 183 |  |  |  | 457.0 | 92601.5 | 10.7 | 42.8 | 12 | W/B/L |
| Rubia pubescens Nakai | 5 |  | V |  | 331.5 | 19886.2 | 67.4 | 4.9 | 3 | W/R/S |
| Rubus corchorifolius L.f. | 98 |  |  |  | 349.0 | 39974.0 | 6.1 | 56.9 | 6 | W/R/L |
| Rubus coreanus Miq. | 152 |  |  |  | 430.9 | 68469.4 | 8.5 | 50.5 | 7 | W/B/L |
| Rubus crataegifolius Bunge | 578 |  |  |  | 495.2 | 102418.8 | 6.3 | 79.2 | 12 | W/B/L |
| Rubus hirsutus Thunb. | 11 |  |  |  | 244.6 | 22328.7 | 25.1 | 9.8 | 5 | N/B/L |
| Rubus matsumuranus var. concolor (Kom.) Kitag. | 3 |  |  |  | 104.3 | 3.1 | 35.1 | 3.0 | 2 | N/R/S |
| Rubus matsumuranus var. matsumuranus H.Lev. & Vaniot | 13 |  |  |  | 300.0 | 33941.9 | 30.6 | 9.8 | 4 | N/B/L |
| Rubus oldhamii Miq. | 314 |  |  |  | 428.7 | 86576.5 | 8.1 | 53.1 | 8 | W/B/L |
| Rubus palmatus Thunb. | 2 |  |  |  | 314.1 | NA | 314.1 | 1.0 | 1 | N/R/S |
| Rubus parvifolius for. parvifolius L. | 378 |  |  |  | 490.2 | 104053.4 | 8.0 | 61.2 | 12 | W/B/L |
| Rubus phoenicolasius for. phoenicolasius Maxim. | 206 |  |  |  | 386.1 | 78891.5 | 9.2 | 42.0 | 9 | W/B/L |
| Rudbeckia bicolor Nutt. | 41 |  |  |  | 384.4 | 53271.7 | 21.3 | 18.0 | 6 | W/R/S |
| Rudbeckia laciniata L. | 10 |  |  |  | 236.7 | 18616.4 | 33.5 | 7.1 | 5 | N/B/L |
| Rudbeckia laciniata var. hortensis Bailey | 2 |  |  |  | 23.0 | NA | 23.0 | 1.0 | 2 | N/R/S |
| Rumex acetosa L. | 165 |  |  |  | 444.7 | 96729.5 | 11.3 | 39.2 | 10 | W/B/L |
| Rumex acetosella L. | 129 |  |  |  | 458.6 | 94305.2 | 13.6 | 33.7 | 8 | W/B/L |
| Rumex conglomeratus Murray | 11 |  |  |  | 427.0 | 68387.4 | 58.0 | 7.4 | 4 | W/R/S |
| Rumex crispus L. | 244 |  |  |  | 460.9 | 99677.2 | 9.7 | 47.4 | 12 | W/B/L |
| Rumex japonicus Houtt. | 33 |  |  |  | 427.3 | 54541.0 | 23.1 | 18.5 | 6 | W/R/S |
| Rumex maritimus L. | 6 |  |  |  | 314.6 | 19706.7 | 73.7 | 4.3 | 4 | N/B/S |
| Rumex nipponicus Franch. & Sav. | 3 |  |  |  | 259.3 | 8293.2 | 130.2 | 2.0 | 3 | N/B/S |
| Rumex obtusifolius L. | 45 |  |  |  | 424.6 | 80761.9 | 19.7 | 21.5 | 7 | W/B/S |
| Sacciolepis indica (L.) Chase | 25 |  |  |  | 370.1 | 59885.3 | 32.1 | 11.5 | 7 | W/B/S |
| Sacciolepis indica var. oryzetorum (Makino) Ohwi | 5 |  |  |  | 411.3 | 14093.8 | 79.5 | 5.2 | 3 | W/R/S |
| Sageretia thea (Osbeck) M. C. Johnst. | 7 |  |  |  | 275.7 | 2636.8 | 40.8 | 6.8 | 4 | N/B/L |
| Sagina japonica (Sw.) Ohwi | 37 |  |  |  | 469.3 | 79215.4 | 32.9 | 14.2 | 5 | W/R/S |
| Sagittaria aginashi Makino | 24 |  |  |  | 336.6 | 61680.6 | 28.3 | 11.9 | 7 | W/B/S |
| Sagittaria sagittifola subsp. leucopelata var. leucopetala (Miq.) Hartog | 25 |  |  |  | 358.8 | 45867.5 | 21.0 | 17.1 | 6 | W/R/S |
| Salicornia europaea | 2 |  |  |  | 129.8 | NA | 129.8 | 1.0 | 2 | N/R/S |
| Salix babylonica L. | 9 |  |  |  | 229.1 | 20877.4 | 37.9 | 6.1 | 5 | N/B/S |
| Salix caprea L. | 216 |  |  |  | 410.7 | 77159.7 | 8.7 | 47.4 | 10 | W/B/L |
| Salix chaenomeloides var. chaenomeloides Kimura | 40 |  |  |  | 415.2 | 60884.2 | 23.4 | 17.7 | 5 | W/R/S |
| Salix chaenomeloides var. pilosa (Nakai) Kimura | 2 |  | V |  | 211.0 | NA | 211.0 | 1.0 | 2 | N/R/S |
| Salix dependens Nakai | 2 |  | V |  | 188.5 | NA | 188.5 | 1.0 | 2 | N/R/S |
| Salix gilgiana Seem. | 33 |  |  |  | 401.6 | 49449.6 | 20.7 | 19.4 | 6 | W/R/S |
| Salix graciliglans Nakai | 34 |  |  |  | 411.4 | 60348.1 | 17.8 | 23.1 | 5 | W/R/S |
| Salix gracilistyla Miq. | 209 |  |  |  | 417.4 | 88812.1 | 9.0 | 46.4 | 12 | W/B/L |
| Salix hallaisanensis for. hallaisanensis H.Lev. | 35 |  | V |  | 343.6 | 46620.1 | 16.8 | 20.5 | 6 | W/R/S |
| Salix integra Thunb. | 4 |  |  |  | 300.6 | 14854.9 | 106.6 | 2.8 | 3 | N/B/S |
| Salix kangensis Nakai | 2 |  | V |  | 114.3 | NA | 114.3 | 1.0 | 2 | N/R/S |
| Salix koreensis Andersson | 156 |  |  |  | 435.4 | 88348.2 | 11.2 | 38.7 | 10 | W/B/L |
| Salix koriyanagi for. koriyanagi Kimura | 105 |  |  |  | 372.8 | 68153.6 | 10.5 | 35.5 | 10 | W/B/L |
| Salix matsudana for. tortuosa Rehder | 5 |  |  |  | 154.5 | 1218.9 | 8.8 | 17.6 | 3 | N/B/L |
| Salix maximowiczii Kom. | 3 |  |  |  | 164.4 | 136.3 | 65.8 | 2.5 | 3 | N/B/S |
| Salix pseudolasiogyne H.Lev. | 7 |  | V |  | 359.9 | 38086.0 | 85.8 | 4.2 | 4 | W/R/S |
| Salix purpurea var. smithiana Trautv. | 6 |  |  |  | 153.5 | 9434.7 | 35.2 | 4.4 | 3 | N/B/S |
| Salix rorida var. rorida Laksch. | 12 |  |  |  | 344.6 | 37701.7 | 52.7 | 6.5 | 4 | W/R/S |
| Salix subfragilis Andersson | 7 |  |  |  | 237.1 | 12178.3 | 51.0 | 4.6 | 5 | N/B/S |
| Salix xerophila for. xerophila Flod. | 5 |  |  |  | 265.4 | 17102.6 | 86.9 | 3.1 | 3 | N/B/S |
| Salsola collina Pall. | 3 |  |  |  | 280.6 | 87.3 | 95.0 | 3.0 | 2 | N/R/S |
| Salsola komarovii Iljin | 4 |  |  |  | 392.7 | 48494.9 | 185.9 | 2.1 | 4 | W/R/S |
| Salvia chanroenica Nakai | 31 |  | V |  | 452.2 | 52330.6 | 23.3 | 19.4 | 4 | W/R/S |
| Salvia japonica Thunb. | 28 |  |  |  | 181.8 | 8125.3 | 8.8 | 20.7 | 4 | N/B/L |
| Salvia plebeia R.Br. | 80 |  |  |  | 426.1 | 86470.1 | 15.9 | 26.9 | 11 | W/B/S |
| Sambucus sieboldiana var. miquelii | 13 |  |  |  | 270.2 | 25591.6 | 23.6 | 11.5 | 6 | N/B/L |
| Sambucus sieboldiana var. miquelii (Nakai) Hara | 223 |  |  |  | 447.6 | 88741.2 | 8.8 | 51.1 | 11 | W/B/L |
| Sanguisorba hakusanensis Makino | 10 |  | V |  | 443.7 | 21854.1 | 20.1 | 22.1 | 4 | W/R/S |
| Sanguisorba longifolia Bertol. | 3 |  |  |  | 413.2 | 10857.6 | 170.7 | 2.4 | 1 | W/R/S |
| Sanguisorba officinalis L. | 480 |  |  |  | 495.5 | 104319.1 | 7.1 | 70.3 | 12 | W/B/L |
| Sanguisorba tenuifolia var. tenuifolia Fisch. ex Link | 11 |  |  |  | 430.2 | 61817.5 | 57.9 | 7.4 | 4 | W/R/S |
| Sanicula chinensis Bunge | 180 |  |  |  | 441.8 | 83497.9 | 9.1 | 48.6 | 7 | W/B/L |
| Sanicula rubriflora F.Schmidt ex Maxim. | 10 |  |  |  | 189.3 | 4273.3 | 17.2 | 11.0 | 3 | N/B/L |
| Sanicula tuberculata Maxim. | 28 |  |  |  | 346.8 | 50260.1 | 25.5 | 13.6 | 4 | W/R/S |
| Sapium japonicum (Siebold & Zucc.) Pax & Hoffm. | 99 |  |  |  | 315.2 | 47432.9 | 8.2 | 38.5 | 8 | W/B/L |
| Sasa borealis (Hack.) Makino | 146 |  |  |  | 441.8 | 76556.7 | 9.0 | 49.3 | 5 | W/R/L |
| Sasa japonica (Siebold & Zucc. ex Steud.) Makino | 89 |  |  |  | 438.1 | 68096.4 | 11.0 | 39.8 | 9 | W/B/L |
| Saussurea calcicola Nakai | 6 |  | V |  | 98.7 | 1187.2 | 19.6 | 5.0 | 2 | N/R/S |
| Saussurea chabyoungsanica H.T.Im | 3 |  | V |  | 29.4 | 35.3 | 11.5 | 2.6 | 1 | N/R/S |
| Saussurea conandrifolia Nakai | 2 |  | V |  | 143.3 | NA | 143.3 | 1.0 | 1 | N/R/S |
| Saussurea eriophylla Nakai | 5 |  |  |  | 295.7 | 25654.6 | 55.2 | 5.4 | 3 | N/B/S |
| Saussurea gracilis Maxim. | 71 |  |  |  | 470.0 | 73972.3 | 14.7 | 32.1 | 6 | W/R/L |
| Saussurea grandifolia Maxim. | 44 |  |  |  | 384.7 | 55244.7 | 15.7 | 24.6 | 5 | W/R/S |
| Saussurea japonica (Thunb.) DC. | 8 |  |  |  | 333.7 | 9829.1 | 17.0 | 19.6 | 3 | W/R/S |
| Saussurea macrolepis (Nakai) Kitam. | 20 |  | V |  | 353.9 | 52211.3 | 32.4 | 10.9 | 4 | W/R/S |
| Saussurea maximowiczii Herd | 4 |  |  |  | 279.5 | 11262.3 | 100.0 | 2.8 | 4 | N/B/S |
| Saussurea neoserrata Nakai | 2 |  |  |  | 82.7 | NA | 82.7 | 1.0 | 2 | N/R/S |
| Saussurea odontolepis Sch.Bip. ex Herd | 16 |  |  |  | 345.9 | 38802.4 | 36.6 | 9.4 | 4 | W/R/S |
| Saussurea pseudo-gracilis Kitam. | 7 |  |  |  | 291.6 | 6645.8 | 43.9 | 6.6 | 3 | N/B/L |
| Saussurea pulchella (Fisch.) Fisch. | 56 |  |  |  | 472.9 | 80543.3 | 18.8 | 25.2 | 7 | W/B/S |
| Saussurea seoulensis Nakai | 34 |  | V |  | 355.9 | 46389.0 | 16.5 | 21.6 | 4 | W/R/S |
| Saussurea stenolepis Nakai | 5 |  |  |  | 216.3 | 6224.0 | 44.3 | 4.9 | 3 | N/B/S |
| Saussurea tanakae Franch. & Sav. ex Maxim. | 27 |  |  |  | 326.0 | 42670.3 | 16.2 | 20.1 | 4 | W/R/S |
| Saussurea uchiyamana Nakai | 2 |  | V |  | 20.5 | NA | 20.5 | 1.0 | 1 | N/R/S |
| Saussurea ussuriensis Maxim. | 7 |  |  |  | 192.1 | 3839.4 | 32.6 | 5.9 | 5 | N/B/S |
| Saxifraga fortunei var. incisolobata (Engl. & Irmsch.) Nakai | 67 |  |  |  | 455.8 | 66262.7 | 14.7 | 30.9 | 6 | W/R/L |
| Saxifraga nelsoniana D.Don | 2 |  |  |  | 39.4 | NA | 39.4 | 1.0 | 2 | N/R/S |
| Saxifraga oblongifolia Nakai | 3 |  |  |  | 28.8 | 67.3 | 12.7 | 2.3 | 2 | N/R/S |
| Saxifraga octopetala Nakai | 12 |  | V |  | 317.7 | 25003.3 | 24.6 | 12.9 | 3 | W/R/S |
| Saxifraga stolonifera Meerb. | 16 |  |  |  | 333.2 | 34529.7 | 33.5 | 9.9 | 5 | W/R/S |
| Scabiosa tschiliensis Gruning | 29 |  |  |  | 257.8 | 21364.1 | 8.0 | 32.1 | 5 | N/B/L |
| Schisandra chinensis (Turcz.) Baill. | 109 |  |  |  | 372.6 | 54794.7 | 8.8 | 42.6 | 4 | W/R/L |
| Schizachyrium brevifolium (Sw.) Nees ex Buse | 19 |  |  |  | 300.9 | 37597.8 | 25.4 | 11.9 | 3 | N/B/L |
| Schizopepon bryoniifolium Maxim. | 26 |  |  |  | 343.0 | 41021.8 | 20.2 | 17.0 | 6 | W/R/S |
| Schizophragma hydrangeoides Siebold & Zucc. | 2 |  |  |  | 72.6 | NA | 72.6 | 1.0 | 1 | N/R/S |
| Scilla scilloides (Lindl.) Druce | 250 |  |  |  | 474.1 | 102732.0 | 9.9 | 47.9 | 10 | W/B/L |
| Scirpus juncoides var. hotarui (Ohwi) Ohwi | 20 |  |  |  | 456.9 | 63251.1 | 28.4 | 16.1 | 5 | W/R/S |
| Scirpus karuizawensis Makino | 35 |  |  |  | 406.5 | 76386.9 | 17.7 | 22.9 | 7 | W/B/S |
| Scirpus lacustris var. creber (Fern.) T.Koyama | 8 |  |  |  | 293.1 | 34770.5 | 66.2 | 4.4 | 4 | N/B/S |
| Scirpus maritimus L. | 11 |  |  |  | 457.7 | 53030.0 | 39.2 | 11.7 | 5 | W/R/S |
| Scirpus mucronatus L. | 3 |  |  |  | 238.4 | 807.1 | 83.4 | 2.9 | 2 | N/R/S |
| Scirpus nipponicus Makino | 2 |  |  | NT | 438.6 | NA | 438.6 | 1.0 | 2 | W/R/S |
| Scirpus planiculmis F.Schmidt | 8 |  |  |  | 250.7 | 21677.7 | 45.0 | 5.6 | 6 | N/B/S |
| Scirpus radicans Schkuhr | 5 |  |  |  | 202.9 | 12829.8 | 83.7 | 2.4 | 3 | N/B/S |
| Scirpus triangulatus Roxb. | 8 |  |  |  | 371.7 | 25743.9 | 56.2 | 6.6 | 3 | W/R/S |
| Scirpus triqueter L. | 7 |  |  |  | 339.9 | 52455.2 | 65.6 | 5.2 | 5 | W/R/S |
| Scirpus wallichii Nees | 8 |  |  |  | 429.5 | 32110.8 | 52.3 | 8.2 | 3 | W/R/S |
| Scirpus wichurae var. asiaticus (Beetle) T.Koyama | 48 |  |  |  | 386.0 | 79616.5 | 21.0 | 18.4 | 10 | W/B/S |
| Scopolia japonica Maxim. | 37 |  |  |  | 318.5 | 38916.4 | 14.7 | 21.6 | 4 | W/R/S |
| Scorzonera albicaulis Bunge | 21 |  |  |  | 300.5 | 40029.0 | 22.1 | 13.6 | 4 | N/B/L |
| Scorzonera austriaca subsp. glabra (Rupr.) Lipsch. & Krasch. ex Lipsch. | 3 |  |  |  | 40.3 | 237.1 | 20.9 | 1.9 | 1 | N/R/S |
| Scrophularia buergeriana Miq. | 6 |  |  |  | 295.1 | 12185.9 | 51.3 | 5.7 | 3 | N/B/S |
| Scrophularia grayana Maxim. ex Kom. | 2 |  |  |  | 39.5 | NA | 39.5 | 1.0 | 1 | N/R/S |
| Scrophularia kakudensis Franch. | 42 |  |  |  | 380.9 | 68614.6 | 19.5 | 19.5 | 5 | W/R/S |
| Scrophularia koraiensis Nakai | 26 |  |  |  | 363.4 | 63316.2 | 25.2 | 14.4 | 4 | W/R/S |
| Scutellaria baicalensis Georgi | 7 |  |  |  | 164.7 | 2279.1 | 28.7 | 5.7 | 2 | N/R/S |
| Scutellaria dentata var. alpina | 2 |  |  |  | 37.1 | NA | 37.1 | 1.0 | 1 | N/R/S |
| Scutellaria dependens Maxim. | 4 |  |  |  | 127.0 | 746.8 | 35.9 | 3.5 | 3 | N/B/S |
| Scutellaria fauriei H.Lev. & Vaniot | 29 |  |  |  | 413.9 | 56203.6 | 27.0 | 15.3 | 5 | W/R/S |
| Scutellaria indica L. | 164 |  |  |  | 488.0 | 92185.8 | 10.7 | 45.8 | 8 | W/B/L |
| Scutellaria indica var. parvifolia (Makino) Makino | 4 |  |  |  | 210.1 | 11811.1 | 80.1 | 2.6 | 3 | N/B/S |
| Scutellaria insignis Nakai | 23 |  | V |  | 357.4 | 51126.0 | 24.7 | 14.5 | 6 | W/R/S |
| Scutellaria pekinensis Maxim. | 150 |  |  |  | 450.6 | 97332.1 | 10.3 | 43.8 | 9 | W/B/L |
| Scutellaria pekinensis var. ussuriensis (Regel) Hand.-Mazz. | 12 |  |  |  | 295.9 | 25053.4 | 17.7 | 16.7 | 3 | N/B/L |
| Scutellaria strigillosa Hemsl. | 11 |  |  |  | 463.0 | 78780.5 | 58.3 | 7.9 | 4 | W/R/S |
| Securinega suffruticosa (Pall.) Rehder | 463 |  |  |  | 489.0 | 102614.1 | 7.0 | 69.7 | 13 | W/B/L |
| Sedum aizoon L. | 64 |  |  |  | 378.5 | 60323.2 | 15.6 | 24.3 | 6 | W/R/S |
| Sedum bulbiferum Makino | 37 |  |  |  | 366.3 | 59038.3 | 26.6 | 13.8 | 5 | W/R/S |
| Sedum japonicum Siebold ex Miq. | 3 |  |  |  | 255.3 | 3420.9 | 101.2 | 2.5 | 2 | N/R/S |
| Sedum kamtschaticum Fisch. & Mey. | 411 |  |  |  | 468.0 | 96520.0 | 6.6 | 70.4 | 10 | W/B/L |
| Sedum middendorffianum Maxim. | 2 |  |  |  | 126.9 | NA | 126.9 | 1.0 | 2 | N/R/S |
| Sedum oryzifolium Makino | 11 |  |  |  | 357.3 | 43876.7 | 27.8 | 12.9 | 4 | W/R/S |
| Sedum polytrichoides Hemsl. | 160 |  |  |  | 469.2 | 91953.9 | 10.8 | 43.5 | 6 | W/R/L |
| Sedum satmentosum Bunge | 235 |  |  |  | 436.7 | 98477.0 | 10.0 | 43.7 | 10 | W/B/L |
| Selaginella involvens (Sw.) Spring | 17 |  |  |  | 204.0 | 20259.3 | 26.7 | 7.7 | 3 | N/B/L |
| Selaginella rossii (Baker) Warb. | 87 |  |  |  | 435.3 | 74522.3 | 13.0 | 33.6 | 5 | W/R/L |
| Selaginella stauntoniana Spring | 2 |  |  |  | 24.0 | NA | 24.0 | 1.0 | 1 | N/R/S |
| Selaginella tamariscina (P.Beauv.) Spring | 120 |  |  |  | 403.6 | 71614.2 | 9.1 | 44.4 | 7 | W/B/L |
| Semiaquilegia adoxoides (DC.) Makino | 43 |  |  |  | 210.1 | 14236.4 | 9.7 | 21.7 | 7 | N/B/L |
| Senecio argunensis Turcz. | 9 |  |  | NT | 179.1 | 11832.7 | 33.5 | 5.3 | 7 | N/B/S |
| Senecio vulgaris L. | 67 |  |  |  | 461.8 | 93924.7 | 15.4 | 30.1 | 7 | W/B/L |
| Serratula coronata var. insularis for. insularis (Iljin) Kitam. | 103 |  |  |  | 474.8 | 85540.4 | 14.9 | 32.0 | 9 | W/B/L |
| Sesamum indicum L. | 3 |  |  |  | 157.6 | 2770.1 | 75.3 | 2.1 | 2 | N/R/S |
| Setaria chondrachne (Steud.) Honda | 4 |  |  |  | 33.5 | 128.3 | 10.9 | 3.1 | 3 | N/B/S |
| Setaria faberii Herrm. | 72 |  |  |  | 428.2 | 74949.0 | 12.9 | 33.3 | 8 | W/B/L |
| Setaria glauca (L.) P.Beauv. | 199 |  |  |  | 453.8 | 92948.0 | 11.0 | 41.2 | 9 | W/B/L |
| Setaria viridis var. major (Gaudin) Petermann | 2 |  |  |  | 34.0 | NA | 34.0 | 1.0 | 1 | N/R/S |
| Setaria viridis var. pachystachys (Franch. & Sav.) Makino & Nemoto | 2 |  |  |  | 54.8 | NA | 54.8 | 1.0 | 2 | N/R/S |
| Setaria viridis var. viridis (L.) P.Beauv. | 424 |  |  |  | 467.2 | 104179.6 | 7.8 | 59.6 | 11 | W/B/L |
| Setaria xpycnocoma (Steud.) Henrard ex Nakai | 20 |  |  |  | 277.9 | 31399.5 | 30.3 | 9.2 | 6 | N/B/L |
| Sicyos angulatus L. | 15 |  |  |  | 323.9 | 42270.0 | 23.5 | 13.8 | 7 | W/B/S |
| Sigesbeckia glabrescens Makino | 157 |  |  |  | 442.4 | 87380.8 | 11.6 | 38.1 | 10 | W/B/L |
| Sigesbeckia pubescens Makino | 127 |  |  |  | 422.0 | 88793.4 | 11.2 | 37.6 | 9 | W/B/L |
| Silene aprica var. apica Turcz. ex Fisch. & C.A.Mey. | 14 |  |  |  | 399.8 | 32316.7 | 36.6 | 10.9 | 7 | W/B/S |
| Silene aprica var. oldhamiana (Miq.) C.Y.Wu | 2 |  |  |  | 82.8 | NA | 82.8 | 1.0 | 1 | N/R/S |
| Silene armeria L. | 35 |  |  |  | 304.4 | 45357.9 | 18.4 | 16.6 | 7 | N/B/L |
| Silene firma for. pubescens (Makino) Makino | 3 |  |  |  | 258.7 | 369.9 | 90.7 | 2.9 | 3 | N/B/S |
| Silene firma Siebold & Zucc. | 265 |  |  |  | 481.2 | 92975.2 | 9.7 | 49.6 | 10 | W/B/L |
| Silene jenisseensis Willd. | 2 |  |  | VU | 85.4 | NA | 85.4 | 1.0 | 1 | N/R/S |
| Silene koreana Kom. | 7 |  |  | LC | 213.7 | 10520.8 | 22.9 | 9.3 | 3 | N/B/L |
| Silene seoulensis Nakai | 93 |  |  |  | 359.5 | 66231.6 | 11.8 | 30.4 | 8 | W/B/L |
| Sinomenium acutum (Thunb.) Rehder & E.H.Wilson | 5 |  |  |  | 414.1 | 10614.4 | 47.9 | 8.7 | 3 | W/R/S |
| Siphonostegia chinensis Benth. | 26 |  |  |  | 431.3 | 59654.5 | 21.5 | 20.0 | 5 | W/R/S |
| Sisymbrium luteum (Maxim.) O.E.Schulz | 35 |  |  |  | 415.1 | 43432.0 | 14.1 | 29.5 | 4 | W/R/L |
| Sisyrinchium atlanticum Bicknell | 3 |  |  |  | 218.6 | 2939.9 | 91.7 | 2.4 | 1 | N/R/S |
| Sium ninsi L. | 23 |  |  |  | 332.2 | 60339.5 | 30.4 | 10.9 | 7 | W/B/S |
| Sium suave Walter | 54 |  |  |  | 490.6 | 79008.5 | 15.7 | 31.3 | 8 | W/B/L |
| Smilacina japonica var. japonica A.Gray | 140 |  |  |  | 416.5 | 79788.0 | 10.0 | 41.6 | 6 | W/R/L |
| Smilax china L. | 580 |  |  |  | 485.4 | 106799.5 | 5.0 | 96.6 | 10 | W/B/L |
| Smilax china var. microphylla Nakai | 3 |  |  |  | 194.1 | 567.5 | 72.1 | 2.7 | 3 | N/B/S |
| Smilax nipponica Miq. | 308 |  |  |  | 480.5 | 96586.2 | 8.3 | 57.6 | 8 | W/B/L |
| Smilax riparia var. ussuriensis (Regel) Hara & T.Koyama | 195 |  |  |  | 490.2 | 96518.3 | 10.4 | 47.0 | 9 | W/B/L |
| Smilax sieboldii for. intermis (Nakai) Hara | 87 |  |  |  | 426.5 | 80753.9 | 14.1 | 30.3 | 10 | W/B/L |
| Smilax sieboldii for. sieboldii Miq. | 337 |  |  |  | 480.9 | 104726.8 | 7.4 | 65.1 | 11 | W/B/L |
| Solanum americanum Mill. | 18 |  |  |  | 384.4 | 36827.4 | 26.3 | 14.6 | 6 | W/R/S |
| Solanum carolinense L. | 5 |  |  |  | 298.1 | 22346.0 | 55.9 | 5.3 | 3 | N/B/S |
| Solanum japonense Nakai | 12 |  |  |  | 327.2 | 48692.0 | 42.5 | 7.7 | 3 | W/R/S |
| Solanum lyratum Thunb. ex Murray | 119 |  |  |  | 409.6 | 81396.0 | 11.8 | 34.6 | 7 | W/B/L |
| Solanum nigrum var. nigrum L. | 217 |  |  |  | 469.9 | 100752.6 | 11.0 | 42.7 | 10 | W/B/L |
| Solidago altissima L. | 6 |  |  |  | 347.2 | 25435.6 | 61.5 | 5.6 | 3 | W/R/S |
| Solidago serotina Aiton | 19 |  |  |  | 375.1 | 48899.8 | 25.5 | 14.7 | 6 | W/R/S |
| Solidago virg-aurea subsp. asiatica var. asiatica Kitam. ex Hara | 404 |  |  |  | 495.7 | 103349.0 | 8.1 | 60.8 | 10 | W/B/L |
| Sonchus asper (L.) Hill | 47 |  |  |  | 411.7 | 80523.9 | 19.4 | 21.2 | 7 | W/B/S |
| Sonchus brachyotus DC. | 28 |  |  |  | 431.8 | 73517.3 | 26.7 | 16.2 | 6 | W/R/S |
| Sonchus oleraceus L. | 91 |  |  |  | 442.1 | 96266.5 | 14.0 | 31.6 | 9 | W/B/L |
| Sophora flavescens Solander ex Aiton | 318 |  |  |  | 489.0 | 101743.6 | 8.5 | 57.7 | 11 | W/B/L |
| Sophora japonica L. | 8 |  |  |  | 294.1 | 34370.1 | 39.5 | 7.4 | 5 | N/B/L |
| Sorbaria sorbifolia for. incerta (C.K.Schneid.) Kitag. | 2 |  |  |  | 11.9 | NA | 11.9 | 1.0 | 1 | N/R/S |
| Sorbaria sorbifolia var. stellipila Maxim. | 49 |  |  |  | 285.2 | 42966.0 | 16.2 | 17.6 | 4 | N/B/L |
| Sorbus alnifolia (Siebold & Zucc.) K.Koch | 247 |  |  |  | 477.5 | 101808.6 | 8.6 | 55.5 | 11 | W/B/L |
| Sorbus amurensis Koehne | 4 |  |  |  | 141.5 | 2378.6 | 42.3 | 3.3 | 3 | N/B/S |
| Sorbus commixta Hedl. | 24 |  |  |  | 352.3 | 40691.6 | 22.0 | 16.0 | 4 | W/R/S |
| Sorghum bicolor (L.) Moench | 4 |  |  |  | 227.4 | 5705.6 | 73.2 | 3.1 | 4 | N/B/S |
| Spergularia marina (L.) Griseb. | 4 |  |  |  | 390.8 | 50633.2 | 183.9 | 2.1 | 3 | W/R/S |
| Spergularia rubra J.Presl & C.Presl | 2 |  |  |  | 239.5 | NA | 239.5 | 1.0 | 2 | N/R/S |
| Sphenomeris chinensis (L.) Maxon | 7 |  |  |  | 264.9 | 32728.2 | 67.7 | 3.9 | 4 | N/B/S |
| Spiraea blumei G.Don | 106 |  |  |  | 395.6 | 58788.1 | 7.9 | 50.2 | 11 | W/B/L |
| Spiraea cantoniensis Lour. | 3 |  |  |  | 328.4 | 13973.7 | 177.0 | 1.9 | 3 | W/R/S |
| Spiraea chamaedryfolia L. | 8 |  |  |  | 203.4 | 11360.6 | 35.3 | 5.8 | 5 | N/B/S |
| Spiraea chinensis Maxim. | 23 |  |  |  | 257.6 | 14463.0 | 6.9 | 37.1 | 4 | N/B/L |
| Spiraea fritschiana Schneid | 45 |  |  |  | 444.9 | 54264.3 | 15.0 | 29.7 | 6 | W/R/L |
| Spiraea japonica L.f. | 3 |  |  |  | 157.1 | 3617.8 | 82.1 | 1.9 | 2 | N/R/S |
| Spiraea miyabei Koidz. | 4 |  |  |  | 51.9 | 687.0 | 19.9 | 2.6 | 3 | N/B/S |
| Spiraea prunifolia for. simpliciflora Nakai | 347 |  |  |  | 449.9 | 90645.4 | 8.5 | 53.1 | 13 | W/B/L |
| Spiraea pubescens Turcz. | 23 |  |  |  | 292.8 | 27288.4 | 16.7 | 17.5 | 3 | N/B/L |
| Spiraea salicifolia L. | 85 |  |  |  | 277.7 | 38035.5 | 10.6 | 26.1 | 7 | N/B/L |
| Spiraea thunbergii Siebold ex Blume | 10 |  |  |  | 269.9 | 30188.0 | 38.6 | 7.0 | 4 | N/B/L |
| Spiraea trichocarpa Nakai | 24 |  |  |  | 232.5 | 8091.4 | 13.7 | 17.0 | 5 | N/B/L |
| Spiranthes sinensis (Pers.) Ames | 120 |  |  |  | 473.0 | 96669.6 | 14.7 | 32.2 | 10 | W/B/L |
| Spirodela polyrhiza (L.) Sch. | 24 |  |  |  | 365.5 | 54148.7 | 22.0 | 16.6 | 8 | W/B/S |
| Spodiopogon cotulifer (Thunb.) Hack. | 98 |  |  |  | 449.0 | 85969.1 | 13.5 | 33.3 | 8 | W/B/L |
| Spodiopogon sibiricus Trin. | 321 |  |  |  | 461.1 | 100988.0 | 9.0 | 51.5 | 10 | W/B/L |
| Sporobolus fertilis Clayton | 58 |  |  |  | 428.3 | 64655.8 | 14.5 | 29.5 | 8 | W/B/L |
| Sporobolus japonicus (Steud.) Maxim. ex Rendle | 22 |  |  |  | 364.4 | 39104.7 | 20.8 | 17.5 | 4 | W/R/S |
| Stachys japonica Miq. | 111 |  |  |  | 423.2 | 83167.1 | 14.7 | 28.8 | 9 | W/B/L |
| Stachys japonica var. hispidula Hara | 2 |  |  |  | 102.3 | NA | 102.3 | 1.0 | 2 | N/R/S |
| Staphylea bumalda DC. | 418 |  |  |  | 491.4 | 92247.7 | 6.3 | 77.7 | 11 | W/B/L |
| Stauntonia hexaphylla (Thunb.) Decne. | 6 |  |  |  | 148.9 | 1247.7 | 9.6 | 15.5 | 2 | N/R/L |
| Stegnogramma pozoi subsp. mollisima (Fisch. ex Kunze) K.Iwats. | 2 |  |  |  | 108.3 | NA | 108.3 | 1.0 | 1 | N/R/S |
| Stellaria alsine var. undulata (Thunb.) Ohwi | 188 |  |  |  | 459.8 | 98196.3 | 11.9 | 38.6 | 9 | W/B/L |
| Stellaria aquatica (L.) Scop. | 426 |  |  |  | 476.1 | 103714.8 | 7.2 | 66.4 | 11 | W/B/L |
| Stellaria media (L.) Vill. | 157 |  |  |  | 448.3 | 95730.4 | 12.6 | 35.6 | 8 | W/B/L |
| Stephanandra incisa var. incisa (Thunb.) Zabel | 649 |  |  |  | 495.2 | 105618.8 | 5.7 | 86.8 | 12 | W/B/L |
| Stephania japonica (Thunb.) Miers | 3 |  |  |  | 389.0 | 3045.0 | 184.8 | 2.1 | 2 | W/R/S |
| Stewartia koreana Nakai ex Rehder | 42 |  | V |  | 322.0 | 33052.6 | 13.0 | 24.8 | 6 | W/R/S |
| Stipa coreana Honda ex Nakai | 7 |  |  |  | 163.3 | 4193.2 | 24.1 | 6.8 | 4 | N/B/L |
| Stipa pekinensis Hance | 91 |  |  |  | 400.6 | 77431.3 | 13.4 | 29.8 | 8 | W/B/L |
| Streptolirion volubile Edgew. | 55 |  |  |  | 347.5 | 61610.1 | 18.0 | 19.3 | 6 | W/R/S |
| Streptopus amplexifolius var. papillatus Ohwi | 4 |  |  |  | 162.7 | 4988.0 | 53.3 | 3.1 | 2 | N/R/S |
| Streptopus ovalis var. ovalis (Ohwi) F.T.Wang & Y.C.Tang | 29 |  |  |  | 289.6 | 36147.2 | 18.9 | 15.4 | 3 | N/B/L |
| Styrax japonicus Siebold & Zucc. | 376 |  |  |  | 446.2 | 101765.8 | 6.3 | 71.3 | 10 | W/B/L |
| Styrax obassia Siebold & Zucc. | 303 |  |  |  | 476.1 | 89281.5 | 7.0 | 68.4 | 11 | W/B/L |
| Styrax shiraianus Makino | 2 |  |  |  | 137.3 | NA | 137.3 | 1.0 | 2 | N/R/S |
| Suaeda glauca (Bunge) Bunge | 8 |  |  |  | 336.6 | 21015.4 | 28.6 | 11.8 | 5 | W/R/S |
| Suaeda japonica Makino | 5 |  |  |  | 331.0 | 11329.6 | 52.0 | 6.4 | 3 | W/R/S |
| Suaeda maritima (L.) Dumort. | 2 |  |  |  | 88.9 | NA | 88.9 | 1.0 | 2 | N/R/S |
| Swertia diluta var. tosaensis (Makino) Toyok. | 7 |  |  | LC | 257.3 | 12065.5 | 51.1 | 5.0 | 4 | N/B/S |
| Swertia japonica (Schult.) Griseb. | 29 |  |  |  | 218.5 | 26738.7 | 20.4 | 10.7 | 5 | N/B/L |
| Swertia pseudochinensis (Hara) Toyok. | 46 |  |  |  | 406.6 | 74260.6 | 20.9 | 19.4 | 6 | W/R/S |
| Swertia tetrapetala (Pall.) Grossh. | 2 |  |  |  | 113.2 | NA | 113.2 | 1.0 | 2 | N/R/S |
| Swertia wilfordii J.Kern. | 8 |  |  | VU | 160.5 | 700.3 | 20.8 | 7.7 | 4 | N/B/L |
| Symphytum officinale L. | 11 |  |  |  | 265.3 | 14832.8 | 29.2 | 9.1 | 4 | N/B/L |
| Symplocarpus nipponicus Makino | 2 |  |  | LC | 244.0 | NA | 244.0 | 1.0 | 2 | N/R/S |
| Symplocarpus renifolius Schott ex Miq. | 14 |  |  |  | 144.6 | 4389.2 | 11.4 | 12.6 | 3 | N/B/L |
| Symplocos chinensis for. pilosa (Nakai) Ohwi | 478 |  |  |  | 484.0 | 100864.9 | 6.2 | 77.5 | 11 | W/B/L |
| Symplocos tanakana Nakai | 74 |  |  |  | 354.9 | 60311.8 | 11.3 | 31.4 | 8 | W/B/L |
| Syneilesis aconitifolia (Bunge) Maxim. | 4 |  |  |  | 80.1 | 320.4 | 23.5 | 3.4 | 3 | N/B/S |
| Syneilesis palmata (Thunb.) Maxim. | 319 |  |  |  | 470.1 | 98578.3 | 7.3 | 64.1 | 11 | W/B/L |
| Synurus deltoides (Aiton) Nakai | 110 |  |  |  | 405.9 | 69088.7 | 12.1 | 33.6 | 7 | W/B/L |
| Synurus excelsus (Makino) Kitam. | 5 |  |  |  | 194.4 | 6961.6 | 53.5 | 3.6 | 4 | N/B/S |
| Syringa oblata var. dilatata (Nakai) Rehder | 13 |  |  |  | 406.2 | 67386.3 | 49.9 | 8.1 | 5 | W/R/S |
| Syringa patula (Palib.) Nakai | 7 |  |  |  | 293.5 | 12553.3 | 53.2 | 5.5 | 3 | N/B/S |
| Syringa reticulata var. mandshurica (Maxim.) H.Hara | 27 |  |  |  | 253.3 | 19378.4 | 10.7 | 23.7 | 5 | N/B/L |
| Syringa reticulata var. reticulata (Blume) H.Hara | 2 |  |  |  | 68.9 | NA | 68.9 | 1.0 | 1 | N/R/S |
| Syringa vulgaris L. | 6 |  |  |  | 160.5 | 6231.7 | 40.1 | 4.0 | 5 | N/B/S |
| Syringa wolfii C.K.Schneid. | 14 |  |  |  | 246.9 | 21898.6 | 29.6 | 8.3 | 5 | N/B/L |
| Tagetes minuta L. | 39 |  |  |  | 358.1 | 45493.1 | 17.2 | 20.8 | 6 | W/R/S |
| Tagetes patula L. | 5 |  |  |  | 320.0 | 3687.2 | 63.6 | 5.0 | 3 | W/R/S |
| Taraxacum coreanum Nakai | 103 |  |  |  | 412.8 | 78793.6 | 14.2 | 29.1 | 7 | W/B/L |
| Taraxacum officinale Weber | 327 |  |  |  | 463.3 | 103965.9 | 8.7 | 53.0 | 12 | W/B/L |
| Taraxacum ohwianum Kitam. | 22 |  |  |  | 343.8 | 50411.0 | 26.5 | 13.0 | 4 | W/R/S |
| Taraxacum platycarpum Dahlst. | 171 |  |  |  | 454.4 | 94852.9 | 12.0 | 37.8 | 9 | W/B/L |
| Taxodium distichum (L.) Rich. | 4 |  |  |  | 215.9 | 6100.6 | 62.9 | 3.4 | 3 | N/B/S |
| Taxus cuspidata Siebold & Zucc. | 28 |  |  |  | 359.6 | 56979.6 | 28.6 | 12.6 | 3 | W/R/S |
| Tephroseris kirilowii (Turcz. ex DC.) Holub | 135 |  |  |  | 374.6 | 79868.8 | 12.4 | 30.2 | 9 | W/B/L |
| Tephroseris phaeantha (Nakai) C.Jeffrey & Y.L.Chen | 2 |  |  | LC | 1.2 | NA | 1.2 | 1.0 | 1 | N/R/S |
| Tephroseris pseudosonchus (Vaniot) C.Jeffrey & Y.L.Chen | 9 |  |  |  | 402.3 | 63676.0 | 74.4 | 5.4 | 5 | W/R/S |
| Tetragonia tetragonoides (Pall.) Kuntze | 7 |  |  |  | 294.7 | 10650.9 | 46.3 | 6.4 | 4 | N/B/L |
| Teucrium japonicum Houtt. | 32 |  |  |  | 311.1 | 51107.2 | 16.7 | 18.6 | 5 | N/B/L |
| Teucrium viscidum var. miquelianum (Maxim.) Hara | 41 |  |  |  | 404.6 | 68598.6 | 22.5 | 18.0 | 5 | W/R/S |
| Thalictrum actaefolium Siebold & Zucc. | 25 |  |  |  | 325.1 | 45851.6 | 13.2 | 24.6 | 5 | W/R/S |
| Thalictrum actaeifolium var. brevistylum Nakai | 77 |  | V |  | 439.9 | 79259.9 | 12.1 | 36.3 | 5 | W/R/L |
| Thalictrum aquilegifolium var. sibiricum Regel & Tiling | 161 |  |  |  | 440.8 | 88542.3 | 10.3 | 42.8 | 9 | W/B/L |
| Thalictrum coreanum H.Lev. | 2 | V |  | EN | 257.1 | NA | 257.1 | 1.0 | 2 | N/R/S |
| Thalictrum ichangense Lecoy. ex Oliv. | 4 |  |  | NT | 17.9 | 74.7 | 6.4 | 2.8 | 2 | N/R/S |
| Thalictrum kemense var. hypoleucum (Siebold & Zucc.) Kitag. | 106 |  |  |  | 467.1 | 82473.7 | 11.6 | 40.2 | 7 | W/B/L |
| Thalictrum rochebrunianum var. grandisepalum (H.Lev.) Nakai | 17 |  | V |  | 282.4 | 30202.5 | 25.5 | 11.1 | 5 | N/B/L |
| Thalictrum simlex var. brevipes Hara | 7 |  |  |  | 192.3 | 10662.0 | 27.7 | 6.9 | 3 | N/B/L |
| Thalictrum tuberiferum Maxim. | 116 |  |  |  | 480.3 | 74636.5 | 12.6 | 38.2 | 7 | W/B/L |
| Thalictrum uchiyamai Nakai | 35 |  | V |  | 365.7 | 56886.9 | 18.4 | 19.9 | 5 | W/R/S |
| Thelypteris beddomei (Baker) Ching | 3 |  |  |  | 370.1 | 15165.2 | 178.0 | 2.1 | 3 | W/R/S |
| Thelypteris glanduligera var. glanduligera (Kunze) Ching | 28 |  |  |  | 259.7 | 17672.5 | 11.4 | 22.9 | 4 | N/B/L |
| Thelypteris japonica var. japonica (Baker) Ching | 80 |  |  |  | 435.2 | 80729.5 | 14.6 | 29.7 | 7 | W/B/L |
| Thelypteris laxa (Franch. & Sav.) Ching | 9 |  |  |  | 451.5 | 19488.2 | 52.3 | 8.6 | 3 | W/R/S |
| Thelypteris palustris (Salisb.) Schott | 84 |  |  |  | 439.0 | 77461.3 | 13.0 | 33.8 | 8 | W/B/L |
| Thelypteris viridifrons Tagawa | 2 |  |  |  | 71.9 | NA | 71.9 | 1.0 | 1 | N/R/S |
| Themeda triandra var. japonica (Willd.) Makino | 230 |  |  |  | 486.0 | 98429.5 | 9.9 | 49.2 | 10 | W/B/L |
| Thesium chinense Turcz. | 147 |  |  |  | 488.7 | 101904.9 | 12.6 | 38.8 | 9 | W/B/L |
| Thesium refractum C.A.Mey. | 3 |  |  |  | 283.1 | 16168.4 | 169.9 | 1.7 | 2 | N/R/S |
| Thladiantha dubia Bunge | 2 |  |  |  | 9.7 | NA | 9.7 | 1.0 | 1 | N/R/S |
| Thlaspi arvense L. | 168 |  |  |  | 413.1 | 80810.3 | 11.4 | 36.1 | 8 | W/B/L |
| Thymus quinquecostatus Celak. | 8 |  |  |  | 284.3 | 17096.9 | 52.3 | 5.4 | 3 | N/B/S |
| Tilia amurensis Rupr. | 77 |  |  |  | 442.4 | 69563.8 | 13.1 | 33.8 | 6 | W/R/L |
| Tilia mandshurica Rupr. & Maxim. | 54 |  |  |  | 399.6 | 61603.1 | 16.8 | 23.7 | 3 | W/R/S |
| Tilia miqueliana Maxim. | 3 |  |  |  | 422.9 | 24870.4 | 215.7 | 2.0 | 2 | W/R/S |
| Tilia rufa Nakai | 20 |  |  |  | 421.6 | 59256.9 | 30.9 | 13.7 | 4 | W/R/S |
| Tilia taquetii C.K.Schneid. | 11 |  |  |  | 429.1 | 32541.9 | 48.7 | 8.8 | 4 | W/R/S |
| Torilis japonica (Houtt.) DC. | 217 |  |  |  | 447.3 | 98992.5 | 11.1 | 40.3 | 10 | W/B/L |
| Torilis scabra (Thunb.) DC. | 35 |  |  |  | 338.8 | 59377.2 | 23.4 | 14.5 | 5 | W/R/S |
| Torreya nucifera (L.) Siebold & Zucc. | 16 |  |  |  | 408.6 | 47185.3 | 37.1 | 11.0 | 5 | W/R/S |
| Toxicodendron succedaneum (L.) Kuntze | 13 |  |  |  | 300.2 | 35907.4 | 24.2 | 12.4 | 4 | N/B/L |
| Trachelospermum asiaticum var. asiaticum (Siebold & Zucc.) Nakai | 96 |  |  |  | 275.5 | 39209.9 | 7.9 | 34.7 | 8 | N/B/L |
| Trachelospermum asiaticum var. majus (Nakai) Ohwi | 4 |  |  |  | 152.1 | 1462.4 | 44.9 | 3.4 | 2 | N/R/S |
| Trachelospermum jasminoides var. pubescens Makino | 23 |  |  |  | 157.4 | 10891.8 | 9.6 | 16.4 | 5 | N/B/L |
| Tradescantia reflexa Raf. | 23 |  |  |  | 362.7 | 52558.5 | 31.4 | 11.5 | 5 | W/R/S |
| Tragopogon dubius Scop. | 3 |  |  |  | 140.2 | 635.1 | 58.8 | 2.4 | 2 | N/R/S |
| Trapa japonica Flerow | 39 |  |  |  | 366.2 | 65112.5 | 18.7 | 19.5 | 7 | W/B/S |
| Triadenum japonicum (Blume) Makino | 3 |  |  |  | 422.0 | 26731.7 | 238.9 | 1.8 | 2 | W/R/S |
| Trichosanthes kirilowii Maxim. | 52 |  |  |  | 357.0 | 71680.8 | 18.0 | 19.9 | 8 | W/B/S |
| Trichosanthes kirilowii var. japonica Kitam. | 6 |  |  |  | 282.3 | 15671.0 | 59.5 | 4.7 | 4 | N/B/S |
| Tricyrtis macropoda Miq. | 53 |  |  | LC | 385.4 | 53112.4 | 14.1 | 27.3 | 7 | W/B/S |
| Trientalis europaea var. arctica (Fisch.) Ledeb. | 2 | V |  |  | 64.3 | NA | 64.3 | 1.0 | 1 | N/R/S |
| Trifolium hybridum L. | 5 |  |  |  | 122.9 | 1329.8 | 30.4 | 4.0 | 4 | N/B/S |
| Trifolium pratense L. | 114 |  |  |  | 437.4 | 90962.0 | 13.4 | 32.6 | 10 | W/B/L |
| Trifolium repens L. | 311 |  |  |  | 475.5 | 100246.9 | 9.4 | 50.8 | 9 | W/B/L |
| Triglochin maritimum L. | 2 |  |  |  | 331.0 | NA | 331.0 | 1.0 | 2 | W/R/S |
| Trigonotis icumae (Maxim.) Makino | 37 |  |  | LC | 335.9 | 43296.0 | 17.9 | 18.8 | 5 | W/R/S |
| Trigonotis peduncularis (Trevir.) Benth. ex Hemsl. | 350 |  |  |  | 467.8 | 96366.6 | 9.1 | 51.6 | 12 | W/B/L |
| Trigonotis radicans var. sericea (Maxim.) H.Hara | 145 |  |  |  | 434.5 | 84012.1 | 12.5 | 34.7 | 11 | W/B/L |
| Trillium camschatcense Ker Gawl. | 2 |  |  | LC | 34.1 | NA | 34.1 | 1.0 | 1 | N/R/S |
| Trillium kamtschaticum Pall. ex Pursh | 14 |  |  |  | 144.8 | 4107.5 | 10.2 | 14.3 | 3 | N/B/L |
| Tripterygium regelii Sprague & Takeda | 157 |  |  |  | 413.9 | 78543.2 | 9.5 | 43.6 | 8 | W/B/L |
| Trisetum bifidum (Thunb.) Ohwi | 68 |  |  |  | 423.8 | 71872.4 | 19.5 | 21.7 | 6 | W/R/S |
| Trisetum sibiricum Rupr. | 2 |  |  |  | 58.5 | NA | 58.5 | 1.0 | 1 | N/R/S |
| Triumfetta japonica Makino | 20 |  |  |  | 317.2 | 36470.4 | 20.7 | 15.3 | 7 | W/B/S |
| Tulipa edulis (Miq.) Baker | 77 |  |  |  | 434.5 | 81250.3 | 14.6 | 29.8 | 5 | W/R/L |
| Tylophora floribunda Miq. | 12 |  |  |  | 252.8 | 27557.9 | 30.4 | 8.3 | 3 | N/B/L |
| Typha angustifolia L. | 27 |  |  |  | 462.5 | 85983.3 | 25.3 | 18.3 | 8 | W/B/S |
| Typha latifolia L. | 2 |  |  |  | 11.4 | NA | 11.4 | 1.0 | 1 | N/R/S |
| Typha orientalis C.Presl | 41 |  |  |  | 419.1 | 78440.2 | 22.5 | 18.6 | 10 | W/B/S |
| Ulmus davidiana for. suberosa Nakai | 31 |  |  |  | 304.4 | 35008.0 | 17.0 | 17.9 | 4 | N/B/L |
| Ulmus davidiana Planch. | 32 |  |  |  | 395.9 | 63793.2 | 21.2 | 18.6 | 6 | W/R/S |
| Ulmus davidiana var. japonica (Rehder) Nakai | 133 |  |  |  | 392.8 | 81253.5 | 11.2 | 35.2 | 8 | W/B/L |
| Ulmus laciniata (Trautv.) Mayr | 20 |  |  |  | 226.3 | 8996.6 | 13.0 | 17.3 | 4 | N/B/L |
| Ulmus macrocarpa Hance | 14 |  |  |  | 136.2 | 6744.7 | 15.7 | 8.7 | 4 | N/B/L |
| Ulmus parvifolia Jacq. | 54 |  |  |  | 463.3 | 87336.3 | 20.4 | 22.7 | 6 | W/R/S |
| Urtica angustifolia Fisch. ex Hornem. | 43 |  |  |  | 285.7 | 41552.4 | 11.6 | 24.7 | 6 | N/B/L |
| Urtica laetevirens Maxim. | 26 |  |  |  | 309.6 | 34420.9 | 19.8 | 15.6 | 6 | N/B/L |
| Urtica thunbergiana Siebold & Zucc. | 34 |  |  |  | 413.5 | 72790.2 | 24.3 | 17.0 | 5 | W/R/S |
| Utricularia bifida L. | 3 |  |  | LC | 254.1 | 34.6 | 84.9 | 3.0 | 1 | N/R/S |
| Utricularia japonica Makino | 6 |  |  |  | 252.1 | 21921.8 | 31.7 | 8.0 | 4 | N/B/L |
| Utricularia racemosa Wall. | 2 |  |  | LC | 159.4 | NA | 159.4 | 1.0 | 2 | N/R/S |
| Vaccaria vulgaris Host | 2 |  |  |  | 191.7 | NA | 191.7 | 1.0 | 2 | N/R/S |
| Vaccinium bracteatum Thunb. | 14 |  |  |  | 53.7 | 479.6 | 1.8 | 29.9 | 4 | N/B/L |
| Vaccinium hirtum var. koreanum (Nakai) Kitam. | 102 |  | V |  | 362.6 | 52998.0 | 9.1 | 39.7 | 5 | W/R/L |
| Vaccinium oldhamii Miq. | 117 |  |  |  | 343.6 | 41300.2 | 7.6 | 45.5 | 6 | W/R/L |
| Valeriana fauriei Briq. | 192 |  |  |  | 469.1 | 94233.5 | 9.6 | 48.8 | 11 | W/B/L |
| Veratrum maackii var. japonicum (Baker) T.Schmizu | 145 |  |  |  | 458.9 | 78664.3 | 11.3 | 40.6 | 8 | W/B/L |
| Veratrum maackii var. maackii Regel | 9 |  |  |  | 295.6 | 20720.8 | 48.1 | 6.1 | 3 | N/B/L |
| Veratrum maackii var. parviflorum (Maxim.) Hara | 31 |  |  |  | 384.9 | 52795.7 | 24.7 | 15.6 | 4 | W/R/S |
| Veratrum nigrum var. ussuriense Lose.f. | 3 |  |  |  | 98.2 | 396.6 | 45.1 | 2.2 | 2 | N/R/S |
| Veratrum patulum Loes. | 35 |  |  |  | 287.8 | 30525.5 | 15.6 | 18.5 | 5 | N/B/L |
| Veratrum versicolor for. viride Nakai | 6 |  |  |  | 285.3 | 28860.5 | 16.0 | 17.8 | 7 | N/B/L |
| Veratrum versicolor Nakai | 49 |  |  |  | 317.0 | 45330.5 | 72.6 | 4.4 | 3 | W/R/S |
| Verbesina alternifolia Britton | 8 |  |  |  | 358.9 | 31667.4 | 56.2 | 6.4 | 4 | W/R/S |
| Veronica anagallisaquatica L. | 4 |  |  |  | 275.7 | 20241.5 | 102.2 | 2.7 | 2 | N/R/S |
| Veronica arvensis L. | 119 |  |  |  | 442.3 | 88583.1 | 13.4 | 33.0 | 9 | W/B/L |
| Veronica didyma var. lilacina (H.Hara) T.Yamaz. | 78 |  |  |  | 347.3 | 68102.0 | 15.4 | 22.5 | 6 | W/R/S |
| Veronica kiusiana var. glabrifolia (Kitag.) Kitag. | 6 |  | V |  | 363.5 | 15438.8 | 56.1 | 6.5 | 2 | W/R/S |
| Veronica linariifolia for. lnariifolia Pall. ex Link | 15 |  |  |  | 254.7 | 31629.8 | 24.4 | 10.4 | 5 | N/B/L |
| Veronica longifolia L. | 11 |  |  |  | 321.2 | 18149.0 | 18.0 | 17.9 | 3 | W/R/S |
| Veronica peregrina L. | 5 |  |  |  | 276.1 | 25592.6 | 108.9 | 2.5 | 4 | N/B/S |
| Veronica persica Poir. | 134 |  |  |  | 411.9 | 88756.4 | 9.8 | 42.0 | 10 | W/B/L |
| Veronica rotunda var. subintegra (Nakai) T.Yamaz. | 18 |  |  |  | 360.0 | 44266.2 | 39.3 | 9.1 | 3 | W/R/S |
| Veronica undulata Wall. | 23 |  |  |  | 347.5 | 35663.9 | 18.1 | 19.2 | 6 | W/R/S |
| Veronicastrum sibiricum (L.) Pennell | 9 |  |  |  | 134.6 | 7166.7 | 24.7 | 5.4 | 3 | N/B/S |
| Viburnum burejaeticum Regel & Herder | 24 |  |  |  | 349.3 | 52321.8 | 10.4 | 33.5 | 6 | W/R/L |
| Viburnum carlesii Hemsl. | 80 |  |  |  | 414.7 | 82289.7 | 12.6 | 32.9 | 6 | W/R/L |
| Viburnum dilatatum Thunb. ex Murray | 211 |  |  |  | 462.5 | 84843.0 | 7.6 | 61.2 | 10 | W/B/L |
| Viburnum erosum Thunb. | 265 |  |  |  | 445.7 | 95950.9 | 6.0 | 73.8 | 11 | W/B/L |
| Viburnum odoratissimum var. awabuki (K.Koch) Zabel ex Rumpler | 3 |  |  |  | 68.1 | 152.5 | 25.6 | 2.7 | 3 | N/B/S |
| Viburnum opulus for. hydrangeoides (Nakai) Hara | 12 |  |  |  | 321.4 | 38955.1 | 50.5 | 6.4 | 4 | W/R/S |
| Viburnum opulus L. | 110 |  |  |  | 376.9 | 68929.5 | 12.2 | 30.8 | 8 | W/B/L |
| Viburnum wrightii Miq. | 81 |  |  |  | 371.1 | 73912.8 | 9.9 | 37.6 | 7 | W/B/L |
| Viburnum wrightii var. stipllatum Nakai | 3 |  |  |  | 59.9 | 194.3 | 26.4 | 2.3 | 2 | N/R/S |
| Vicia amoena Fisch. ex DC. | 134 |  |  |  | 433.5 | 90197.9 | 12.7 | 34.0 | 9 | W/B/L |
| Vicia amurensis Oett. | 111 |  |  |  | 455.1 | 77320.1 | 10.9 | 41.9 | 10 | W/B/L |
| Vicia angustifolia var. minor (Bertol.) Ohwi | 14 |  |  |  | 253.2 | 36519.5 | 29.5 | 8.6 | 4 | N/B/L |
| Vicia angustifolia var. segetilis (Thuill.) K.Koch. | 58 |  |  |  | 400.2 | 76776.7 | 16.8 | 23.8 | 6 | W/R/S |
| Vicia angustipinnata Nakai | 5 |  | V |  | 259.7 | 9050.2 | 61.3 | 4.2 | 3 | N/B/S |
| Vicia bungei Ohwi | 7 |  |  |  | 323.3 | 9320.2 | 45.6 | 7.1 | 5 | W/R/S |
| Vicia chosenensis Ohwi | 111 |  | V |  | 395.8 | 56869.4 | 10.0 | 39.6 | 6 | W/R/L |
| Vicia cracca L. | 35 |  |  |  | 440.7 | 70795.5 | 24.4 | 18.0 | 9 | W/B/S |
| Vicia hirsuta (L.) Gray | 27 |  |  |  | 247.0 | 26052.3 | 19.1 | 12.9 | 5 | N/B/L |
| Vicia hirticalycina Nakai | 7 |  |  |  | 186.0 | 4736.9 | 24.9 | 7.5 | 4 | N/B/L |
| Vicia japonica A.Gray | 30 |  |  |  | 389.2 | 62894.5 | 21.1 | 18.4 | 9 | W/B/S |
| Vicia nipponica Matsum. | 35 |  |  |  | 427.0 | 71416.4 | 24.7 | 17.3 | 7 | W/B/S |
| Vicia pseudoorobus Fisch. & C.A.Mey. | 24 |  |  |  | 284.7 | 37016.9 | 18.3 | 15.5 | 6 | N/B/L |
| Vicia tetrasperma (L.) Schreb. | 62 |  |  |  | 394.3 | 74906.5 | 15.6 | 25.3 | 8 | W/B/S |
| Vicia unijuga A.Braun | 300 |  |  |  | 470.3 | 92516.4 | 8.0 | 59.0 | 9 | W/B/L |
| Vicia venosa (Willd.) Maxim. | 12 |  |  |  | 263.9 | 19763.5 | 35.8 | 7.4 | 3 | N/B/L |
| Vicia venosa var. albiflora (Turcz.) Maxim. | 7 |  |  |  | 262.8 | 22604.9 | 57.3 | 4.6 | 4 | N/B/S |
| Vicia venosa var. cuspidata Maxim. | 118 |  |  |  | 420.4 | 72423.6 | 10.4 | 40.4 | 6 | W/R/L |
| Vicia villosa Roth | 8 |  |  |  | 199.8 | 8364.2 | 33.9 | 5.9 | 5 | N/B/S |
| Vigna angularis var. nipponensis (Ohwi) Ohwi & H.Ohashi | 169 |  |  |  | 453.2 | 89265.0 | 11.8 | 38.5 | 10 | W/B/L |
| Vigna radiata (L.) Wilczek | 8 |  |  |  | 265.7 | 30197.5 | 37.6 | 7.1 | 4 | N/B/L |
| Vigna umbellata (Thunb.) Ohwi & Ohashi | 13 |  |  |  | 221.7 | 24681.6 | 32.8 | 6.8 | 5 | N/B/L |
| Vigna vexillata var. tsusimensis Matsum. | 18 |  |  |  | 338.6 | 48463.0 | 30.0 | 11.3 | 4 | W/R/S |
| Viola acuminata Ledeb. | 455 |  |  |  | 488.0 | 96643.8 | 6.5 | 74.7 | 10 | W/B/L |
| Viola albida for. takahashii (Makino) W.T.Lee | 41 |  |  |  | 389.3 | 71979.6 | 20.8 | 18.7 | 4 | W/R/S |
| Viola albida Palib. | 99 |  |  |  | 412.0 | 73474.0 | 12.9 | 31.8 | 5 | W/R/L |
| Viola albida var. chaerophylloides (Regel) F.Maek. ex Hara | 468 |  |  |  | 482.6 | 104434.7 | 6.7 | 72.2 | 12 | W/B/L |
| Viola blandaeformis Nakai | 2 |  |  |  | 152.1 | NA | 152.1 | 1.0 | 2 | N/R/S |
| Viola brevistipulata var. minor Nakai | 3 |  |  |  | 184.6 | 1039.3 | 68.7 | 2.7 | 2 | N/R/S |
| Viola collina Besser | 153 |  |  |  | 447.1 | 86010.4 | 11.7 | 38.3 | 10 | W/B/L |
| Viola diamantiaca Nakai | 50 |  |  | LC | 337.3 | 58241.2 | 16.6 | 20.3 | 5 | W/R/S |
| Viola grypoceras A.Gray | 49 |  |  |  | 415.0 | 80255.0 | 19.3 | 21.4 | 8 | W/B/S |
| Viola hirtipes S.Moore | 19 |  |  |  | 382.9 | 55916.7 | 26.7 | 14.3 | 4 | W/R/S |
| Viola ibukiana Makino | 2 |  |  |  | 122.6 | NA | 122.6 | 1.0 | 2 | N/R/S |
| Viola japonica Langsd. ex Ging. | 105 |  |  |  | 427.0 | 81515.7 | 13.4 | 31.8 | 9 | W/B/L |
| Viola keiskei Miq. | 179 |  |  |  | 408.1 | 81961.4 | 9.8 | 41.7 | 8 | W/B/L |
| Viola lactiflora Nakai | 34 |  |  |  | 414.7 | 65407.7 | 20.2 | 20.5 | 6 | W/R/S |
| Viola mandshurica W.Becker | 500 |  |  |  | 464.2 | 100070.9 | 6.5 | 71.2 | 12 | W/B/L |
| Viola orientalis (Maxim.) W.Becker | 189 |  |  |  | 436.0 | 87879.2 | 8.8 | 49.3 | 6 | W/R/L |
| Viola ovato-oblonga (Miq.) Makino | 2 |  |  |  | 3.3 | NA | 3.3 | 1.0 | 2 | N/R/S |
| Viola papilionacea Pursh | 2 |  |  |  | 128.2 | NA | 128.2 | 1.0 | 2 | N/R/S |
| Viola patrinii DC. ex Ging. | 106 |  |  |  | 426.5 | 77809.3 | 12.9 | 33.0 | 8 | W/B/L |
| Viola phalacrocarpa for. glaberrima (W.Becker) F.Maek. ex H.Hara | 2 |  |  |  | 175.9 | NA | 175.9 | 1.0 | 2 | N/R/S |
| Viola phalacrocarpa Maxim. | 128 |  |  |  | 404.7 | 85791.8 | 11.2 | 36.0 | 9 | W/B/L |
| Viola rossii Hemsl. | 327 |  |  |  | 455.4 | 97220.9 | 7.3 | 62.0 | 12 | W/B/L |
| Viola sacchalinensis H.Boissieu | 3 |  |  |  | 277.5 | 19195.3 | 193.3 | 1.4 | 2 | N/R/S |
| Viola selkirkii for. selkirkii Pursh ex (Goldie) | 94 |  |  |  | 418.1 | 79215.2 | 14.5 | 28.8 | 5 | W/R/L |
| Viola seoulensis Nakai | 26 |  | V |  | 416.6 | 52377.1 | 23.9 | 17.5 | 7 | W/B/S |
| Viola tokubuchiana var. takedana (Makino) F.Maek. | 33 |  |  |  | 334.5 | 51349.5 | 14.0 | 23.9 | 5 | W/R/S |
| Viola tokubuchiana var. takedana for. variegata F.Maek. | 3 |  |  |  | 182.5 | 4167.9 | 89.7 | 2.0 | 3 | N/B/S |
| Viola variegata var. chinensis Bunge | 2 |  |  |  | 164.8 | NA | 164.8 | 1.0 | 1 | N/R/S |
| Viola variegata var. ircutiana Regel | 13 |  |  |  | 164.8 | 32870.1 | 164.8 | 1.0 | 1 | N/R/S |
| Viola variegata var. variegata Fisch. ex Link | 225 |  |  |  | 372.2 | 73106.1 | 8.3 | 44.9 | 8 | W/B/L |
| Viola verecunda var. verecunda A.Gray | 210 |  |  |  | 480.2 | 96353.9 | 9.9 | 48.7 | 12 | W/B/L |
| Viola violacea Makino | 2 |  |  |  | 83.6 | NA | 83.6 | 1.0 | 2 | N/R/S |
| Viola yedoensis Makino | 96 |  |  |  | 426.3 | 86821.6 | 15.9 | 26.8 | 8 | W/B/S |
| Viscum album var. coloratum (Kom.) Ohwi | 24 |  |  |  | 266.8 | 22586.9 | 15.4 | 17.3 | 4 | N/B/L |
| Vitex negundo var. cannabifolia (Siebold & Zucc.) Hand.-Mazz. | 3 |  |  |  | 202.0 | 2305.5 | 90.6 | 2.2 | 3 | N/B/S |
| Vitex negundo var. heterophylla L. (Franch.) Rehder | 4 |  |  |  | 227.8 | 19274.2 | 119.1 | 1.9 | 3 | N/B/S |
| Vitex rotundifolia L.f. | 7 |  |  |  | 411.6 | 30848.6 | 54.3 | 7.6 | 3 | W/R/S |
| Vitis amurensis Rupr. | 108 |  |  |  | 445.9 | 89833.3 | 13.9 | 32.1 | 7 | W/B/L |
| Vitis coignetiae Pulliat ex Planch. | 58 |  |  |  | 403.5 | 72312.8 | 14.1 | 28.6 | 6 | W/R/L |
| Vitis ficifolia for. glabrata (Nakai) W.T.Lee | 4 |  | V |  | 0.1 | 0.0 | 0.0 | 4.5 | 1 | N/R/S |
| Vitis ficifolia var. sinuata (Regel) H.Hara | 93 |  |  |  | 427.0 | 93465.4 | 14.9 | 28.6 | 7 | W/B/L |
| Vitis flexuosa Thunb. | 198 |  |  |  | 425.8 | 85629.0 | 8.9 | 47.7 | 10 | W/B/L |
| Vitis flexuosa var. choii T.B.Lee | 2 |  |  |  | 195.3 | NA | 195.3 | 1.0 | 1 | N/R/S |
| Vitis vinifera L. | 2 |  |  |  | 20.0 | NA | 20.0 | 1.0 | 2 | N/R/S |
| Vulpia myuros var. megalura (Nutt.) Auquier | 5 |  |  |  | 287.6 | 13715.3 | 71.0 | 4.1 | 3 | N/B/S |
| Vulpia myuros var. myuros (L.) C.C.Gmelin | 17 |  |  |  | 276.3 | 7621.8 | 10.1 | 27.3 | 5 | N/B/L |
| Waldsteinia ternata (Stephan) Fritsch | 9 |  |  |  | 306.6 | 39583.3 | 63.9 | 4.8 | 4 | N/B/S |
| Weigela florida (Bunge) A.DC. | 157 |  |  |  | 413.3 | 80287.0 | 10.7 | 38.8 | 8 | W/B/L |
| Weigela hortensis (Siebold & Zucc.) K.Koch | 7 |  |  |  | 298.3 | 20705.8 | 62.7 | 4.8 | 3 | N/B/S |
| Weigela subsessilis L.H.Bailey | 452 |  | V |  | 471.8 | 94443.2 | 5.8 | 81.5 | 11 | W/B/L |
| Wikstroemia trichotoma (Thunb.) Makino | 2 |  |  | NT | 193.4 | NA | 193.4 | 1.0 | 2 | N/R/S |
| Wisteria floribunda for. floribunda (Willd.) DC. | 42 |  |  |  | 427.1 | 86000.9 | 25.1 | 17.0 | 7 | W/B/S |
| Wisteria japonica Siebold & Zucc. | 2 |  |  | LC | 45.1 | NA | 45.1 | 1.0 | 1 | N/R/S |
| Woodsia ilvensis (L.) R.Br. | 5 |  |  |  | 269.8 | 20321.5 | 94.6 | 2.9 | 3 | N/B/S |
| Woodsia macrochlaena Mett. ex Kuhn | 9 |  |  |  | 258.2 | 17386.8 | 24.1 | 10.7 | 3 | N/B/L |
| Woodsia manchuriensis Hook. | 68 |  |  |  | 385.3 | 74675.5 | 17.1 | 22.5 | 4 | W/R/S |
| Woodsia polystichoides D.C.Eaton | 97 |  |  |  | 454.2 | 84427.7 | 12.2 | 37.3 | 8 | W/B/L |
| Woodsia subcordata Turcz. | 8 |  |  |  | 246.9 | 29209.4 | 56.8 | 4.3 | 6 | N/B/S |
| Xanthium canadense Mill. | 8 |  |  |  | 338.6 | 34219.3 | 79.3 | 4.3 | 5 | W/R/S |
| Xanthium italicum Moore | 2 |  |  |  | 58.3 | NA | 58.3 | 1.0 | 2 | N/R/S |
| Xanthium strumarium L. | 42 |  |  |  | 439.6 | 83361.3 | 25.6 | 17.2 | 9 | W/B/S |
| Youngia japonica (L.) DC. | 334 |  |  |  | 463.1 | 98078.0 | 8.5 | 54.2 | 9 | W/B/L |
| Yucca filamentosa L. | 2 |  |  |  | 221.4 | NA | 221.4 | 1.0 | 2 | N/R/S |
| Zabelia biflora (Turcz.) Makino | 23 |  |  |  | 95.9 | 3381.3 | 6.9 | 13.8 | 5 | N/B/L |
| Zanthoxylum piperitum (L.) DC. | 221 |  |  |  | 488.2 | 100110.4 | 8.3 | 58.6 | 9 | W/B/L |
| Zanthoxylum planispinum Siebold & Zucc. | 24 |  |  |  | 408.0 | 38088.8 | 10.3 | 39.5 | 5 | W/R/L |
| Zanthoxylum schinifolium Siebold & Zucc. | 701 |  |  |  | 491.6 | 106825.8 | 5.2 | 95.2 | 12 | W/B/L |
| Zanthoxylum schinifolium var. inermis (Nakai) T.B.Lee | 18 |  | V |  | 408.3 | 56609.4 | 28.2 | 14.5 | 5 | W/R/S |
| Zea mays L. | 4 |  |  |  | 70.0 | 470.2 | 22.2 | 3.2 | 4 | N/B/S |
| Zelkova serrata (Thunb.) Makino | 209 |  |  |  | 428.7 | 90293.7 | 9.9 | 43.1 | 7 | W/B/L |
| Zingiber mioga (Thunb.) Roscoe | 5 |  |  |  | 145.5 | 3045.2 | 38.0 | 3.8 | 3 | N/B/S |
| Zingiber officinale Roscoe | 4 |  |  |  | 307.1 | 562.5 | 77.5 | 4.0 | 2 | N/R/S |
| Zizania latifolia (Griseb.) Turcz. ex Stapf | 25 |  |  |  | 380.4 | 52482.0 | 29.0 | 13.1 | 6 | W/R/S |
| Zizyphus jujuba var. inermis (Bunge) Rehder | 19 |  |  |  | 334.0 | 47117.3 | 37.4 | 8.9 | 8 | W/B/S |
| Zizyphus jujuba var. jujuba Mill. | 4 |  |  |  | 98.6 | 529.6 | 9.4 | 10.5 | 2 | N/R/L |
| Zoysia japonica Steud. | 79 |  |  |  | 422.0 | 82404.2 | 15.0 | 28.2 | 8 | W/B/S |
| Zoysia sinica Hance | 2 |  |  |  | 131.2 | NA | 131.2 | 1.0 | 1 | N/R/S |
